# Fast Ultra‐Selective ^1^H‐^15^N 1D NMR Spectroscopy Unlocks Atom‐Resolved Dynamics of Low‐Complexity Protein Regions

**DOI:** 10.1002/anie.202519206

**Published:** 2026-02-17

**Authors:** Wiktor Adamski, Geraldine R. Levy, François‐Xavier Cantrelle, Davy Sinnaeve

**Affiliations:** ^1^ CNRS, Univ. Lille, Institut Pasteur de Lille UMR 9031 – Integrative Structural Biology Lille France

**Keywords:** ^15^N spin‐relaxation, huntingtin, NMR spectroscopy, protein dynamics, selective experiments

## Abstract

Insight into the conformational dynamics of proteins is essential toward understanding their function at a molecular level. The motions experienced by individual atoms in the protein can be precisely quantified through NMR relaxation rates, but their measurement requires well‐resolved spectral responses. Two‐dimensional ^1^H‐^15^N correlation spectra are the standard approach to resolve amide signals in protein NMR, but come with an excessive cost in experimental time when spectra are heavily congested due to limited ^15^N chemical shift dispersions. This limitation often thwarts the characterization of dynamics for intrinsically disordered proteins, especially when they feature low‐complexity or homopolymer regions, or short sample life‐times. Here, we introduce a fast, ultra‐selective ^1^H‐^15^N 1D NMR method that allows high‐quality measurement of individual ^15^N spin‐relaxation constants, even when ^15^N resonances are merely 6–8 Hz apart. We demonstrate the new experiment by characterizing, for the first time, pico‐ to nanosecond dynamics along a 16‐residue polyglutamine stretch within the protein huntingtin, the causal agent of Huntington's disease, as well as millisecond conformational exchange in the SH3GL3 protein. The new experiment will find wide application in the study of conformational dynamics of intrinsically disordered proteins or any other biomacromolecule that features highly dense ^1^H‐^15^N 2D spectra.

## Graphical abstract

NMR ^15^N spin‐relaxation rates are a primary source of information for deciphering protein conformational dynamics, but they require unreasonable experimental times when their ^1^H‐^15^N 2D spectra are highly congested. We propose an ultra‐selective method, SNIPER, that greatly accelerates data collection of individual residues. This allows characterization of protein dynamics even for low‐complexity or homorepeat amino‐acid sequences.

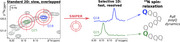

Conformational dynamics are key for protein function. To quantify the conformational motions sensed by individual nuclei, NMR relaxation rates are a primary information source [1, 2, 3]. For this, it is essential that NMR spectral responses are fully resolved, which is usually achieved with multidimensional heteronuclear experiments. Unfortunately, multidimensional acquisition comes at the price of increased minimal experimental time. The smaller the chemical shift differences in the indirect dimension, the more time‐consuming the experiment becomes. The time‐cost is exacerbated for relaxation measurements, as these necessitate recording 2D experiments about ten times with variable delays, radio‐frequency (*rf*) amplitudes or magnetic field strength [1, 2]. When spectra are congested and chemical shift differences are very small — rampant for intrinsically disordered proteins (IDPs) with low sequence‐complexity — complete high‐quality quantification of conformational dynamics would require an excessive experimental time.

Characterization of dynamics across the whole IDP sequence is rarely essential. Often, just a subset of amino acid residues is of functional interest, such as those found near sites for intermolecular interaction or post‐translational modifications [4], or when they need to be monitored under variable experimental conditions such as temperature or the presence of interacting partners [5, 6]. A significant time‐gain can thus be achieved using selective experiments that yield one ^1^H‐^15^N correlation at a time, allowing the use of fast 1D experiments instead of multidimensional acquisition. This also allows a residue‐specific optimization of the experimental setup, such as the number of scans, or the sampling of relaxation‐weighting delays or of *rf*‐filed strengths [5, 6]. This is especially true for experiments measuring conformational exchange using weak *rf*‐fields such as off‐resonance R_1ρ_ relaxation dispersion and Chemical Exchange Saturation Transfer (CEST) [6, 7, 8, 9], where the ^15^N irradiation frequencies must be tailored for each residue, making broadband 2D acquisition very time‐inefficient. Selective methods for such experiments have been proposed for folded proteins with well‐dispersed spectra [6, 7], but do not achieve sufficient selectivity to be applicable to heavily overlapped regions as often encountered in IDPs. Here, we propose a new experiment that achieves a selectivity approaching the ^15^N natural line width while retaining good sensitivity, making even extremely dense spectral regions accessible.

The exon‐1 fragment of the protein huntingtin (htt) presents a clear illustration of the spectral crowding encountered in IDPs. It features a 16‐residue polyglutamine (polyQ) homorepeat, the abnormal expansion of which triggers Huntington's disease [10]. A gradual change of secondary structure adopted along the polyQ has been proposed as central toward understanding pathogenicity [11, 12], implying that characterization of conformational dynamics along this tract using NMR relaxation analysis would be a logical pursuit. Although relaxation rates have been reported for other polyglutamine‐containing proteins using 2D NMR [13, 14], the profound density of the 2D ^1^H‐^15^N HSQC of htt (Figure 1a) has until now obstructed relaxation measurements on individual glutamines [12]. Note that the 2D HSQC in Figure 1a was recorded with a digital resolution along the ^15^N dimension similar to the natural line‐widths, yet much overlap remains. Baseline‐resolved correlations can be achieved using resolution‐enhancing apodization, but at a significant cost in signal‐to‐noise ratio (see Supporting Information). An existing method to facilitate resolving individual residues in polyQ sequences uses additional ^13^C isotope labelling and non‐uniformly sampled (NUS) high‐dimensional experiments [12, 13], but spectra reconstructed from NUS data result in significantly lower fidelity for relaxation analysis [15, 16]. Another strategy involves residue‐specific ^15^N isotopic labeling [11, 17, 18], but these methods have insufficient protein yields for relaxation measurements and require separate protein productions per residue. The ideal approach would cleanly isolate an individual correlation from crowded signal densities at a similar sensitivity and resolution achievable by the standard 2D method, but with a much shorter minimal experimental time and without resorting to sparse data sampling or elaborate isotope labeling.

**FIGURE 1:**
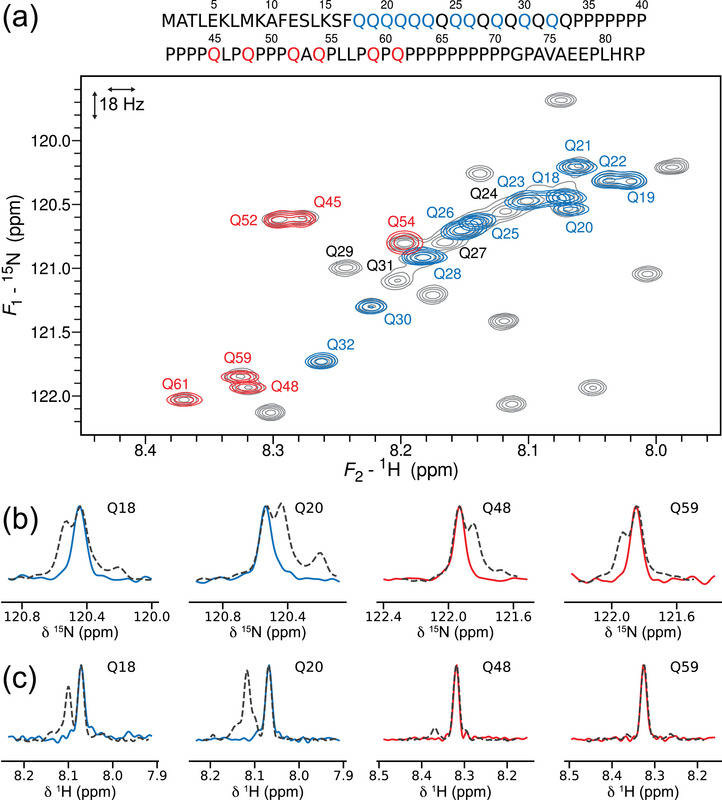
(a) Amino acid sequence of htt exon‐1 polyQ‐16 and a standard ^1^H‐ ^15^N 2D HSQC (900 MHz, 340 µM, pH 6.8, 283 K) (grey), overlayed with several 2D SNIPERs targeting individual glutamine correlations from the polyQ (blue) or the proline‐rich region (red). (b) *F*_1_‐cross‐sections taken from the 2D SNIPER spectra (full line) and the standard 2D HSQC (dashed line) for a number of glutamines. (c) 1D SNIPER spectra (full line) and *F*_2_‐cross‐sections from the standard 2D HSQC (dashed line) for a number of glutamines. The Supporting Information contains more examples. [Correction added on February 25, 2026, after first online publication: Figure 1 has been updated.]

The new NMR approach introduced here, coined SNIPER (*Selected Nuclei Irradiation for Precise and Expedited Relaxation measurements*) meets these requirements. It cleanly delivers individual ^1^H‐^15^N correlations even when they are separated by very small ^15^N frequency differences. Several examples yielding individual glutamine correlations from htt exon‐1 are shown overlayed on the standard ^1^H‐^15^N HSQC in Figure 1a, including from within the polyQ signal density and some that were not cleanly resolved in the standard 2D HSQC without resolution‐enhancing apodization (Q18/Q20 and Q48/Q59). The spectral purity is highly satisfactory (Figure 1b), clearing the way to measure relaxation via fast 1D acquisition (Figure 1c).

Using 1D SNIPER, we obtained a full ^15^N R_1_, R_2_ and {^1^H}‐^15^N nOe dataset for each residue. Figure 2 shows ^15^N relaxation parameters extracted from a broad selection of glutamine signals using SNIPER. The fast 1D acquisition permitted sufficient signal averaging to attain good signal‐to‐noise ratios throughout the entire relaxation decay curves, resulting in high‐quality mono‐exponential fittings (Figures S18 and S19). Within the polyQ, the R_2_ rates appear to be steady and high between Q18 and Q22, substantiating previous work that revealed Q20 and Q21 participate in bifurcated hydrogen bonding interactions with S16 and F17, respectively, stabilizing an α‐helical secondary structure [18, 19]. The subsequent monotonic descent of R_2_ rates toward the C‐terminal end of the polyQ implies a gradual increase in the relative importance of motions occurring on single nanosecond time‐scales [20, 21, 22]. This agrees with previous chemical shift analysis studies indicating that the α‐helical propensity gradually decreases along the polyQ [11, 12, 17, 18]. The R_1_ rates fluctuate along the polyQ, suggesting a diversity of motions on single nanosecond timescales. The {^1^H}‐^15^N nOes drop markedly beyond Q26, pointing toward a relative increase in the importance of motions occurring on the order of hundreds of picosecond time‐scale [20, 21, 22]. This indicates a loss of cooperative motions in the C‐terminal segment of the polyQ and thus lack of stable secondary structures. This is consistent with previous conclusions based on chemical shifts that suggested the helical structure is almost entirely vanished at Q28 for the htt polyQ‐16 sequence [18]. For the glutamines in the proline‐rich region (Q45‐Q61), a remarkably non‐uniform picture emerges for all relaxation parameters. This suggests a diversity of motional behaviors that could be due to yet unidentified specific interresidue contacts and local secondary structures.

**FIGURE 2:**
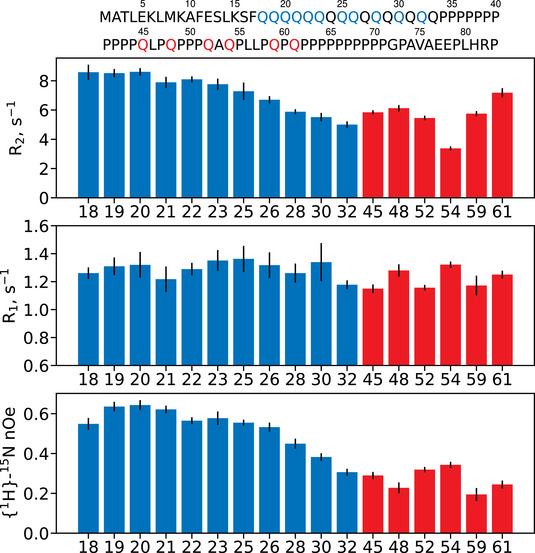
^15^N R_1_, R_2_ rates and {^1^H}‐^15^N nOes (with 95% uncertainties) extracted from htt exon‐1 glutamines using 1D SNIPER.

Figure 3 shows the SNIPER pulse sequences for ^15^N R_1_ and R_2_ measurements (the {^1^H}‐^15^N nOe sequence is found in Figure S12). It applies selective in‐phase ^1^H‐^15^N polarization transfers using matched ^1^H and ^15^N irradiation, either applied simultaneously (Hartmann‐Hahn, HaHa) or consecutively (refocused single field polarization transfer, rSFPT) [4, 7, 24, 25, 26, 27]. This already provides a selectivity of ca. 35‐45 Hz in both ^1^H and ^15^N (see Supporting Information), similar to previous methods [7] but insufficient for very dense spectra such as htt. We therefore implemented a ^15^N chemical‐shift‐selective filter (CSSF), consisting out of a 90°_y_—ζ – 90°_‐y_ sequence applied on longitudinal ^15^N magnetization during ^1^H continuous wave (CW) decoupling, followed by a purge gradient. Previous selective methods used the ζ‐delay to remove ^15^N signals found (4ζ)^−1^ Hz off‐resonance [7], but this results in unacceptably high relaxation losses for very small frequency differences. SNIPER rather achieves its ultra‐high ^15^N selectivity by replacing the first ^15^N 90° pulse in the CSSF by a selective excitation pulse. To cleanly differentiate close ^15^N frequencies, this pulse should feature a sharp excitation profile, without off‐resonance excitation side‐bands, while being as efficient as possible in terms of relaxation losses. We found the classical Half‐Gauss shape [28, 29] to be well‐suited in many cases. For very small ^15^N chemical shift differences (< ca. 12 Hz), however, as found in htt‐exon‐1, we could only achieve sufficient selectivity and sensitivity using a novel pulse shape derived using optimal control theory [30], which we coin GOLEM (*Gradient Optimized Line‐width Exact Matching*) (see Supporting Information). The key feature of GOLEM is that the excitation profile and ^15^N relaxation‐weighting for long pulses (>100 ms) conspire [31] to yield a sharper excitation transition band. The improved ^15^N CSSF results in a satisfying overall ^15^N selectivity of SNIPER that can go as low as 6‐8 Hz, approaching the natural line‐width. This is far superior to what was achieved previously for 1D selective R_1ρ_ experiments [7]. The cost in sensitivity is modest, as relaxation‐weighting during the long GOLEM pulse is largely determined by ^15^N R_1_ rather than R_2_, as demonstrated experimentally in the Supporting Information. For instance, for Q18, one of the fasted relaxing glutamines and just 8 Hz away from Q20, 1D SNIPER was found to deliver a similar signal‐to‐noise ratio than a high‐resolution 2D spectrum, but with an experimental time that is four times less. Importantly, the minimal experimental time of the 2D approach to resolve individual resonances is limited by the sampling of time‐domain points along the indirect dimension. 1D SNIPER does not have this limitation, meaning it can further trade‐in signal‐to‐noise for speed. This is partly compensated for by using a residue‐specific optimized sampling of the relaxation‐weighting delays, delivering reliable relaxation rates even at lower signal‐to‐noise ratios (see Supporting Information) [5].

**FIGURE 3:**
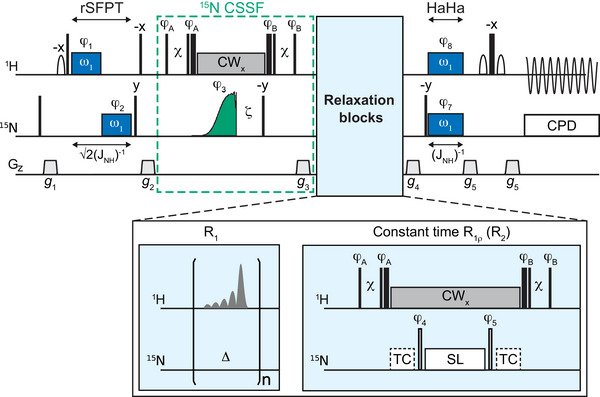
SNIPER pulse sequences for measuring ^15^N R_1_ and R_2_ (via constant‐time R_1ρ_) [7] rates. Thin and wide filled black rectangles are hard 90° and 180° pulses, respectively. The ^15^N selective 90° shaped pulse is colored green. White sine‐bells are ^1^H selective 90° pulses set to the water signal. Dark blue boxes are ^1^H and ^15^N irradiations at matched *rf*‐field strength (ω_1_  =  0.5×*J*_NH_) for selective polarization transfer. Grey rectangles represent CW irradiation of the amide proton. The delay ζ is 0‐5 ms and Δ is 5‐40 ms. χ is a delay matched to the ^1^H CW power so the amide proton and water magnetizations are simultaneously aligned with the CW effective B_1_‐field [23]. ^1^H^N^ selective inversion pulses are shown as grey shapes. The ^15^N R_1ρ_ spin‐lock (SL) is applied on‐resonance and temperature compensation spin‐locks (TC) far off‐resonance. The ^15^N open‐bar rectangles are pulses with flip angles matched to the spin‐lock's offset from the ^15^N resonance of interest [7]. Grey trapezoids are pulsed field gradients. CPD denotes composite pulse decoupling. The Supporting Information contains phase cycling, gradient amplitudes and further technical details.

Fast 1D selective methods are equally beneficial for quantifying millisecond conformational exchange. Standard 2D *zz*‐exchange experiments feature additional ‘cross‐peaks’ between the correlations of the slowly exchanging states (‘diagonal peaks’) [32], often delivering congested spectra even in the case of folded proteins. When the ^15^N chemical shifts of the two exchanging states are close, quantification of the exchange rates via such cross‐peaks requires long 2D data acquisitions [32]. SNIPER can separate the two states using fast 1D spectra and thus also the cross‐peaks between them. The Supporting Information contains an illustration of this principle on the folded SH3 domain of the SH3GL3 protein [33]. Another *zz*‐exchange approach that reported improved ^15^N selectivity is the *F*_1_ *F*_2_‐selective experiment, which combines selective HaHa with 2D acquisition to resolve close ^15^N chemical shifts [34, 35]. However, this results in profoundly longer experimental times and turned out unsuccessful in resolving exchange cross‐peaks that were readily obtained using SNIPER (see Supporting Information).

SNIPER delivers high‐quality, high‐precision atom‐resolved information on conformational dynamics of individual residues in a fraction of the time needed by 2D approaches. It provides access to key functional residues in crowded spectral regions when an exhaustive full spectral analysis is not required or when sample lifetimes are short, such as for aggregation‐prone proteins. This makes SNIPER a powerful complement to recently proposed selective methods for fast spectral assignment, such as FOSY [4]. Importantly, in contrast to these methods, it does not rely on ^13^C labeling. We foresee it to be especially beneficial for experiments whose optimal setup is very residue‐specific, such as off‐resonance R_1ρ_ relaxation dispersion or CEST [6, 7, 8]. Even in cases where ^15^N R_1_, R_2_ and {^1^H}‐^15^N nOe relaxation data from a maximum of residues is required, the most time‐efficient strategy will be to first use the 2D approach for well‐resolved correlations and then apply complementary SNIPER experiments on key residues located in dense spectral regions. For IDPs, which commonly feature very crowded spectra, it will thus provide rapid access to conformational dynamics that previously would have been challenging to collect. The opportunities created by the ability to selectively excite an individual residue with a ^15^N resolution approaching the natural line‐width can be seen as similar to the recent breakthrough of ultra‐selective fast 1D methods in small molecule ^1^H NMR [36, 37, 38, 39]. We expect SNIPER will find wide application in protein dynamics studies, as well as for other biomacromolecules such as RNA.

## Conflicts of Interest

The authors declare no conflicts of interest.

## Supporting information



The authors have cited additional references within the Supporting Information [1–30]. **Supporting File**: anie71519‐sup‐0001‐SuppMat.pdf

## Data Availability

NMR raw data, Bruker pulse sequences and pulse shapes can be downloaded from https://doi.org/10.5281/zenodo.16489356.
