# Supplementary material for: Fast Ultra‐Selective 1H‐15N 1D NMR Spectroscopy Unlocks Atom‐Resolved Dynamics of Low‐Complexity Protein Regions
Source: Angew Chem Int Ed Engl. 2026 Feb 17;65(13):e19206. doi: 10.1002/anie.202519206 (PMC13007572; doi:10.1002/anie.202519206)
Supplement: Supplementary file 1 — The authors have cited additional references within the Supporting Information [1–30]. Supporting File: anie71519‐sup‐0001‐SuppMat.pdf [file ANIE-65-e19206-s001.pdf]

Fast Ultra-Selective ^1H-^{15}N 1D NMR Spectroscopy Unlocks Atom-Resolved Dynamics of Low-Complexity Protein Regions

Wiktor Adamski,*[a] Geraldine R. Levy,[a] François-Xavier Cantrelle[a] and Davy Sinnaeve*[a]

[a] Dr. W. Adamski, Dr. G. R. Levy, Dr. F.-X. Cantrelle and Dr. D. Sinnaeve
CNRS, Univ. Lille, Institut Pasteur de Lille UMR 9031 – Integrative Structural Biology
F-59000, Lille, France
E-mail: wiktor.adamski1992@gmail.com, davy.sinnaeve@univ-lille.fr

Supporting Information

Table of Contents

1. EXPERIMENTAL 4

1.1. HUNTINGTIN EXON-1 SAMPLE PREPARATION 4
1.2. SH3GL3-SH3 SAMPLE PREPARATION 4
1.3. NMR EXPERIMENTAL 5
1.4. PROTEIN ASSIGNMENTS 7
1.5. NMR SIMULATIONS 7
1.6. CREATION OF GOLEM PULSE USING THE GRAPE ALGORITHM 7

2. FURTHER DETAILS OF THE SNIPER PULSE SEQUENCES 9

2.1. SELECTIVE IN-PHASE POLARIZATION TRANSFER 9
2.2. ^{15}N CHEMICAL SHIFT SELECTIVE FILTER 12
2.2.1. GENERAL PRINCIPLES 12
2.2.2. CHOICE OF A 90° SELECTIVE PULSE SHAPE 13
2.3. OPTIONAL ^{1}H SELECTIVE FILTER 19
2.4. R_1 RELAXATION AND *ZZ*-EXCHANGE BLOCK 19
2.5. R_{1P} RELAXATION BLOCK 19
2.6. ^{1}H-^{15}N NOE SNIPER SEQUENCE 20
2.7. PHASE CYCLE AND GRADIENT STRENGTHS 21

3. SIGNAL-TO-NOISE RATIO AND TIME-GAIN OF 1D SNIPER 23

4. EXPERIMENTAL RESULTS ON HUNTINGTIN EXON-1 28

4.1. OVERVIEW OF SNIPER EXPERIMENTS 28
4.2. 1D SNIPER SPECTRA 30
4.3. RELAXATION DATA 31

5. EXPERIMENTAL RESULTS ON THE SH3 DOMAIN OF SH3GL3 35

5.1. SH3GL3-SH3 2D SPECTRA AND ASSIGNMENT 35
5.2. OVERVIEW OF *ZZ*-EXCHANGE EXPERIMENTS 36
5.3. EXCHANGE DATA FITTING PROCEDURE 37
5.4. COMPARISON OF 1D SNIPER, THE STANDARD 2D METHOD AND F_1F_2-SELECTIVE *ZZ*-EXCHANGE EXPERIMENTS 38

6. PRACTICAL SNIPER SETUP 53

6.1. SETUP GUIDELINES 53
6.2. PULSE SEQUENCE BRUKER CODES 55
6.2.1. SNIPER_N15SELE 56

6.2.2. SNIPER_N15R1 66
6.2.3. SNIPER_N15R1RHO 76
6.2.4. SNIPER_N15REX 89
6.2.5. SNIPER_N15NOE 99

7. REFERENCES 106

1. Experimental

1.1. Huntingtin exon-1 sample preparation

For the huntingtin exon-1 protein fragment, the following construct was used with ^{15}N isotope labelling:

```
       10         20         30         40         50         60
MATLEKLMKA FESLKSFQQQ QQQQQQQQQQ QQQPPPPPPP PPPPQLPQPP PQAQPLLPQP
       70         80         90
QPPPPPPPPP PGPAVAEEPL HRPGSSHHHH HH
```

Transformed *E. coli* BL-21(DE3) bacterial cells were grown in M9 minimal media and salts (12.8 g/L of Na_2HPO_4, 3 g/L of KH_2PO_4, 0.5 g of NaCl, 1 mM $MgSO_4$, 50 µM $CaCl_2$, in glass-distilled, autoclaved H_2O containing ampicillin and salts with [^{15}N] ammonium chloride (1 g/l) and [^{12}C] D-glucose (2 g/l), as sole nitrogen and carbon sources. Thereafter, expression was induced with 1 mM isopropyl-beta-D-thiogalactoside (IPTG) for 4h at 37 °C. Cells were harvested by spinning at 4000 rpm for 20 min at 4 °C; pellets were washed with Phosphate Buffered Saline (PBS), spun again, flash frozen in liquid nitrogen, and stored at −80 °C. Pellets were resuspended in 50 mM K-Hepes/pH 7.4, 20 mM Imidazole, 8 M urea, sonicated 3x (30% output, 1 s on/off, 1 min, on ice), and incubated at room temperature for 30 min. Lysates were centrifuged at 12000 rpm for 45 min. Ni^{2+} affinity chromatography was performed using an ÄKTA pure fast protein liquid chromatography (FPLC) system with UV-detection at 215, 260 and 280 nm. The supernatant was loaded onto the column previously equilibrated with 20 mM sodium phosphate Na_2HPO_4, 20 mM Imidazole, 500 mM NaCl, pH 7.4, 0 M urea, filter-sterilized, and was then washed with 10 Column Volumes (CV) of this buffer to remove non-specific binding and urea. The protein was eluted with a gradient of imidazole (20-200 mM Imidazole, 20 mM sodium phosphate Na_2HPO_4, 500 mM NaCl, pH 7.4, 0 M urea). Huntingtin fragment-containing fractions were identified by sodium dodecyl sulfate polyacrylamide gel electrophoresis (SDS-PAGE). Relevant fractions were then exchanged with 50 mM NH_4HCO_3 through dialysis, flash frozen, and lyophilised. Sample was concentrated with a VivaSpin 4 (cutoff 3 kDa, Sartorius Stedim Biotech). Due to the lack of tryptophan residues, the protein concentration was assessed with OD_{214nm}, with molecular weight 10231.57 Da and an estimated absorption coefficient of 219551 $g^{-1}mol.cm^{-1}$.

NMR samples were redissolved in 5 mm standard tubes (600 µl) in milli-Q water and 10% D_2O. The pH was 6.8.

1.2. SH3GL3-SH3 sample preparation

For the SH3 domain of the SH3GL3 protein (also known as endophilin-A3), the following construct was used with ^{15}N and ^{13}C isotope labelling:

```
       10         20         30         40         50         60         70
MDQPCCRGLY DFEPENQGEL GFKEGDIITL TNQIDENWYE GMIHGESGFF PINYVEVIVP LPQGSSHHHH HH
```

Following transformation with a pET15b plasmid, SH3GL3-SH3 was overexpressed in *E. coli* BL21 DE3 strain. Transformed BL-21(DE3) bacterial cells were grown in M9 minimal media and salts (12.8 g/L of Na_2HPO_4, 3 g/L of KH_2PO_4, 0.5 g of NaCl, 1 mM $MgSO_4$, 50 µM $CaCl_2$, in glass-distilled, autoclaved H2O containing ampicillin and salts with [^{15}N] ammonium chloride (1 g/l) and [^{13}C] D-glucose (2 g/l), as sole nitrogen and carbon sources. Thereafter, expression was induced with 1 mM IPTG for 4h at 37 °C. Cells were harvested by spinning at 4000 rpm for

20 min at 4 °C; pellets were washed with PBS, spun again, flash frozen in liquid nitrogen, and stored at −80 °C. Pellets were resuspended in 50 mM K-Hepes/pH 7.4, 20 mM Imidazole, 2-3 passes through cell disruptor on ice. Lysates were centrifuged at 12000 rpm for 45 min. Ni^{2+} affinity chromatography was performed using an ÄKTA pure FPLC system with UV-detection at 215, 260 and 280 nm with the column charged with Ni^{2+}. The supernatant was loaded onto the column previously equilibrated with 20 mM sodium phosphate Na_2HPO_4, 20 mM Imidazole, 500 mM NaCl, pH 7.4, filter-sterilized. The protein was eluted with a gradient of imidazole (20-200 mM Imidazole, 20 mM sodium phosphate Na_2HPO_4, 500 mM NaCl, pH 7.4). SH3GL3-SH3-containing fractions were identified by SDS-PAGE. The selected fractions were pooled and dialyzed twice at 4°C against 2L of 50 mM Na_2HPO_4, 150 mM NaCl, 1 mM DTT, pH 7.4, filter-sterilized and concentrated down to a volume below 5 mL. Size exclusion chromatography was performed using a 120 mL superdex S75 column using 50 mM Na_2HPO_4, 150 mM NaCl, pH 7.4, filter-sterilized buffer. SH3GL3-SH3-containing fractions were identified by SDS-PAGE. Relevant fractions were then exchanged with 50 mM NH_4HCO_3 through dialysis, flash frozen, and lyophilised. The protein concentration was assessed with OD_{280nm} with a molecular weight of 8292.13 Da and an extinction coefficient of 10095 $g^{-1}mol.cm^{-1}$.

Samples for NMR measurements consisted of 300 µM in standard 3 mm tubes (200 µl) using 50 mM phosphate buffer (pH 6.8), 150 mM filter sterilized NaCl, 10% D_2O, and TMSP.

1.3. NMR experimental

NMR experiments on the SH3GL3-SH3 protein were performed on a Bruker Avance III HD spectrometer operating at a ^1H and ^{15}N frequencies of 600.13 MHz and 60.81 MHz, respectively, equipped with a CP-QCI-F cryoprobe and running Topspin 3.6.2. All experiments were performed at 283.0 K. ^1H-^{15}N HSQC experiments with sensitivity improved scheme were performed using the standard pulse sequence from the Bruker library. Spectral windows were 14.02 ppm in F_2 and 30 ppm in F_1, and the total number of time domain points was 2048 in F_2 and 80 in F_1. The number of transients used was 8. For the INEPT-based 2D *zz*-exchange experiments,[1] spectral windows were 13.33 ppm in F_2 and 30 ppm in F_1, and the total number time domain points was 2048 in F_2 and 255 in F_1. The number of transients used was 64. For the F_1F_2-selective 2D *zz*-exchange experiments,[2] spectral windows were 13.33 ppm in F_2 and 200 Hz in F_1, and the total number of time domain points was 1024 in F_2 and 4 or 8 in F_1. The number of transients used was 408 or 512. For the 1D SNIPER *zz*-exchange experiments, the spectral window was set to 13.304 ppm and the total number of time domain points was 1024. The number of transients used was 256 or 512. A recycle delay of 1.6 s was used. The delay ζ in the ^{15}N CSSF was set to 5 ms, while the selective ^{15}N 90° pulse had a single lobe Sinc shape (Sinc1.1000) and was 7.5-11 ms long.

For SNIPER zz-exchange on SH3GL3, the $^1H^N$ inversion pulses applied during the longitudinal relaxation block were selective IBurp2 pulses with a duration of 1.6 ms on-resonance to the amide protons applied at intervals of 5 ms. The pair of ^{13}C hard π pulses had pulse lengths of 47.4 µs, one set to the $^{13}C^{\alpha}$ and the other to the $^{13}C'$ region. This pair of ^{13}C pulses was applied at intervals of 5 ms.

All of the *zz*-exchange experiments on SH3GL3 were conducted with the same delay list: 12 delays in range of 0 to 750 ms. The total experimental times for the entire dataset of the standard INEPT-based 2D experiments, the F_1F_2-selective 2D experiments, and the 1D

SNIPER *zz*-exchange experiments (on 7 chosen residues) was 125 hours, 214 hours and 44.6 hours, respectively.

NMR experiments on the huntingtin exon-1 protein were performed on a Bruker Avance NEO spectrometer operating at a ^{1}H and ^{15}N frequencies of 900.13 MHz and 91.22 MHz, respectively, equipped with a CP-TCI cryoprobe and running Topspin 4.4.0. All experiments were performed at 283.0 K. ^{1}H-^{15}N HSQC experiments with sensitivity improved scheme were performed using standard pulse sequence from the Bruker library. Spectral windows were 12.62 ppm in F_2 and 14 ppm in F_1, and the total number of time domain points was 3072 in F_2 and 512 in F_1. The number of transients used was 4. For the 1D SNIPER R_1, $R_{1\rho}$ and $\{^{1}H\}$-^{15}N nOe experiments, the spectral window was set to 12.62 ppm and the total number of time domain points was between 1024 and 3072. The number of transients used was between 512 and 1920. The delay ζ in the ^{15}N CSSF was set to 0-5 ms. The selective ^{15}N 90° pulse either had a single lobe Sinc shape (Sinc1.1000) and was 22-35 ms long, had a HalfGauss shape and was 10-80 ms long, or had a GOLEM shape and was 100-200 ms long.

For SNIPER R_1 (huntingtin), the $^{1}H^{N}$ inversion pulses applied during the longitudinal relaxation block were selective IBurp2 pulses applied with a duration of 1.066 ms on-resonance to the amide protons. Pulses were applied with an interval delay Δ of 5-40 ms.

For SNIPER $R_{1\rho}$ (huntingtin), the ^{15}N $\{R_{1\rho}$-$R_1\}$ spin-lock power level was set to 500 Hz and was applied on-resonance, which should be sufficient to quench most of the potential exchange contributions.

For SNIPER R_1 and $\{R_{1\rho}$-$R_1\}$ (huntingtin), relaxation-encoding delays were chosen to satisfy a 0.37 (e^{-1}) signal intensity ratio between the longest and the shortest relaxation delay. One delay value (approximately one-third of the longest delay value) was repeated. The total number of delay increments was 10. A recycle delay of 1.6 s was used. For the SNIPER nOe experiments, a saturation/recovery period of 5 s was used and a recycle delay of 2 s.

For huntingtin, the acquisition of one complete set of ^{15}N-backbone relaxation rates (1D SNIPER R_1, $R_{1\rho}$ and nOe) took 7.5-33 hours (16.2 hours on average) per residue, depending on the ^{15}N R_2 and on whether a long selective excitation pulses on ^{15}N was needed.

The WATERGATE element[3] at the end of all SNIPER sequences used single-lobed sinc-bell shaped pulses (Sinc1.1000) of 1.5 ms at 600.13 MHz and 1.0 ms at 900.13 MHz and pulsed field gradients (g_6) of 500 µs.

2D variants of SNIPER to illustrate the ^{15}N selectivity (applied on huntingtin, as in Figure 1 of the main paper) were created by removing the relaxation block and inserting a t_1/2-180°(^{1}H)-t_1/2 sequence just after the first ^{15}N 90° pulse in the back-transfer block, with t_1 an incremented delay. Quadrature detection was achieved using the TTPI scheme with phase shifting performed on the preceding 90° ^{15}N pulse. All other pulses, pulse phases and gradient strengths were identical to those of 1D SNIPER. Spectral windows were 12.62 ppm in F_2 and 0.88 ppm in F_1, and the total number of time domain points was 3072 in F_2 and 32 in F_1. The number of transients used was 128.

All relaxation experiments were processed using nmrPipe and NMRBox[4, 5] and visualized using either Sparky[6] (2D experiments) or the nmrGlue module in Python[7] (1D experiments). Typically, for all 2D spectra, prior to Fourier transform, squared cosine bell window functions were applied in F_2 and Lorenz-to-Gauss resolution enhancement functions in F_1, as well as zero filling until a 4096×2048 or a 4096×4096 real data matrix size was obtained.

1.4. Protein assignments

The backbone of the huntingtin exon-1 protein fragment was assigned at 900.13 MHz using standard biomolecular NMR procedures (2D ^{1}H-^{15}N HSQC or HMQC, and 3D BEST-HNCO, -HNCACB, -HN(CO)CACB, -HN(CA)CO, and HACAN spectra) and comparison with previous assignments of the polyQ backbone.[8-10]

The backbone of the SH3GL3-SH3 domain was assigned at 600.13 MHz using standard biomolecular NMR procedures domain based on 2D ^{1}H-^{15}N HSQC or HMQC, and 3D HNCO, HNCACB, HN(CO)CACB, HN(CA)CO, and standard (H)CC(CO)NH, (H)N(CA)NNH, H(NCA)NNH, (H)N(COCA)NH, H(NCOCA)NH, and comparison with an existing assignment available in the Biomolecular Magnetic Resonance Bank (BMRB), entry 16813.

1.5. NMR simulations

The NMR simulations were carried out using a home-written python script based on the homogeneous Master Equation for a heteronuclear two-spin system (^{1}H-^{15}N) by Allard *et al*.[11] The spin density matrix was evolved using the Liouville-von-Neumann equation considering the effects of chemical shift evolution, heteronuclear *J*-evolution and relaxation.[11] The J_{HN} coupling value was assumed to be –94 Hz, ^{15}N R_2 and R_1 were 10.0 and 2.0 s^{-1}, respectively, and $^{1}H^{N}$ R_2 and R_1 were 46.6 and 2.0 s^{-1}, respectively. For HaHa, the spin density matrix evolution was conducted by applying a weak B_1 field (45 Hz) on both nuclei simultaneously for a duration $(J_{NH})^{-1}$. For rSFPT, the spin density matrix evolution was conducted by applying a weak B_1 field (45 Hz) first on ^{1}H for a duration of $(\sqrt{2}J_{NH})^{-1}$ and then on ^{15}N for a duration of $(\sqrt{2}J_{NH})^{-1}$. When the effect of offset was simulated, this was always done for values ranging from ±200 Hz in both of the ^{1}H and ^{15}N dimensions and with a step size of 2 Hz. Everything was repeated with B_1-field phases of 0° and 180° and averaged to take into account the effects of phase cycling. Also the following ^{15}N selective pulse shapes were used in the simulation: Sinc,[12] $eSNOB_{270}$,[13] HalfGauss[14] and GOLEM. Since experimentally these pulses are always applied under ^{1}H continuous wave decoupling, the Liouville-von-Neumann equation during evolution under these pulses included only the effects from chemical shift evolution and relaxation, but not *J*-coupling evolution.

1.6. Creation of GOLEM pulse using the GRAPE algorithm

The GOLEM pulse shape was obtained using optimal control theory (*GRadient Ascent Pulse Engineering*, GRAPE).[15] The optimization was carried out using an in-house python script to propagate the spin density matrix using Liouville-von-Neumann equation adapted from Cui *et al.*,[16] and followed the procedure as outlined by Skinner *et al*.[15] The initial state was a ^{15}N magnetization vector (M_x, M_y, M_z) set to (0, 0, 1). The target state was the excitation profile (0 to 200 Hz) of a 50 ms Sinc pulse with a maximum B_1 field of 5 Hz, but where for all offsets beyond 8 Hz with the M_x and M_y components were zeroed and the M_z component set to one. Starting from a random series of *rf*-step amplitudes, the pulse shape was optimized, with convergence reached within 2000 iterations. During the optimization, phases of each *rf*-step were left constant and the effects of *J*-coupling and relaxation were neglected to speed up the convergence. All calculations were performed on a machine equipped with 8 core Intel(R) Xeon(R) W-2225 4.1 GHz CPU using 200 offsets, a pulse shape discretized in 1000 points with 2000 control iterations. The optimization was iterated separately and interchangeably for M_x and M_y, 1000 iterations each to foster competition and to avoid averaging between M_x and M_y

term optimization. In the optimization, for the backward propagation from the target (λ), a 50 ms pulse was used, while for the forward propagation from (0, 0, 1) magnetization vector, a 5 ms pulse was used. We found this setup to be the most robust for convergence to the 'optimal' profile. The total optimization time was ca. 12 hours.

2. Further details of the SNIPER pulse sequences

2.1. Selective in-phase polarization transfer

In order to achieve selective in-phase polarization transfer between ^{1}H and ^{15}N (from H_y to N_x magnetizations), we decided to first explore the use of two schemes. The first is a **selective Hartmann-Hahn (HaHa)** scheme, which is a simultaneous irradiation on ^{15}N and ^{1}H for a duration of $(J_{NH})^{-1}$ at matched *rf*-field strength amplitudes of $0.5(J_{NH})$.[17-19] The second is a scheme first described by Pelupessy and Chiarparin,[18] but never put into practice nor extensively analyzed. It applies first a ^{1}H single-field polarization transfer (SFPT) element[20-22] (a weak continuous wave (CW) ^{1}H rf-field of duration $(\sqrt{2}J_{NH})^{-1}$ that transfers a chosen proton's H_y magnetization into $2H_zN_z$ longitudinal two-spin order), immediately followed by a ^{15}N SFPT element (a weak CW ^{15}N rf-field also of duration $(\sqrt{2}J_{NH})^{-1}$ converting $2H_zN_z$ into N_x magnetization). Both ^{1}H and ^{15}N SFPT elements are applied at *rf*-field strength amplitudes of $0.5(J_{NH})$. We refer to this scheme as a **refocused SFPT (rSFPT)** (in analogy to refocused INEPT), which thus has a total duration of $\sqrt{2}(J_{NH})^{-1}$. Note that we decided to use the same *rf*-field strength amplitude for rSFPT as for HaHa, which is slightly lower as what was proposed by Pelupessy and Chiarparin. This was to further gain on sensitivity, as optimized empirically. Here, we compare the properties of selective HaHa and rSFPT using spin density matrix simulations.

Figure S1 shows the resulting N_x magnetization yielded by the selective HaHa or rSFPT schemes as a function of ^{15}N and ^{1}H offsets. For both, the magnetization is at ca. 10% of the on-resonance magnetization at ca. 45 Hz ^{1}H or ^{15}N offsets, and show negative side-bands at ca. 75-80 Hz.

For SNIPER ^{15}N R_1 and $R_{1\rho}$ experiments, the magnetization is transferred back to ^{1}H at the end of the pulse sequence using another selective polarization transfer element, which also adds to the selectivity. Figure S2 shows the multiplicative effect of two HaHa elements or of an rSFPT element combined with HaHa. Both combinations give similar improvements in terms of selectivity: the final magnetization is at ca. 10% of the on-resonance magnetization now at ca. 35 Hz ^{1}H or ^{15}N offsets. The negative excitation side-bands have nearly fully vanished.

In conclusion, both schemes show comparable performance in terms of in selectivity. For slowly tumbling proteins (rotational correlation times > 10 ns), we found there is a slight preference for the rSFPT in terms of sensitivity despite the $\sqrt{2}$ longer duration of the element (Figure S3). This is explained by the ^{15}N magnetization passing through longitudinal two-spin order, meaning the contribution of longitudinal relaxation is higher for rSFPT than HaHa (Figure S4).[18]

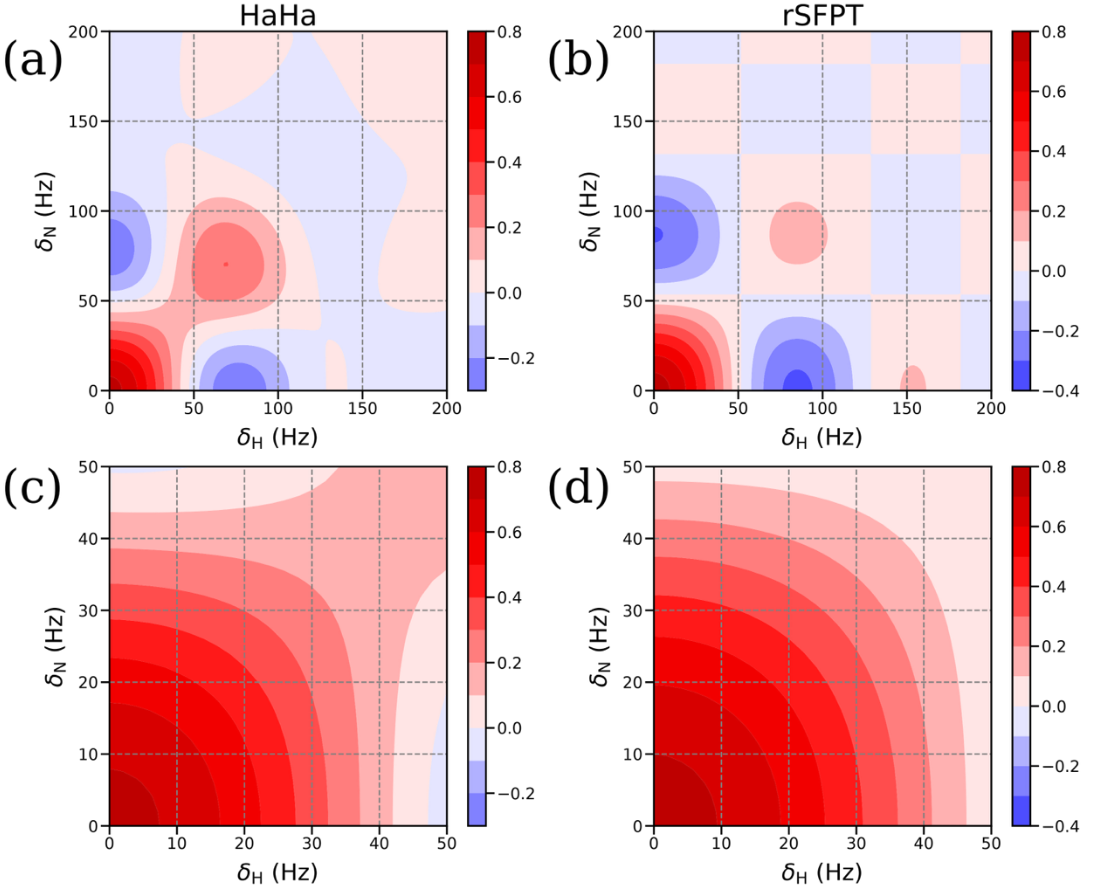

Figure S1. Simulated N_x magnetization as a function of ^{1}H and ^{15}N offset after ^{1}H→^{15}N selective HaHa (a,c) and rSFPT (b,d), starting from H_x magnetization. J_{NH} is assumed –94 Hz. Offsets in the range of 0 to 200 Hz are shown in panels a and b, while panels c and d show the range 0 to 50 Hz. The effects of relaxation were taken into account in this simulation, with ^{15}N R_2 = 10 s^{-1}, $^1H^N$ R_2 = 46.6 s^{-1}, while ^{15}N and $^1H^N$ R_1 = 2 s^{-1}.

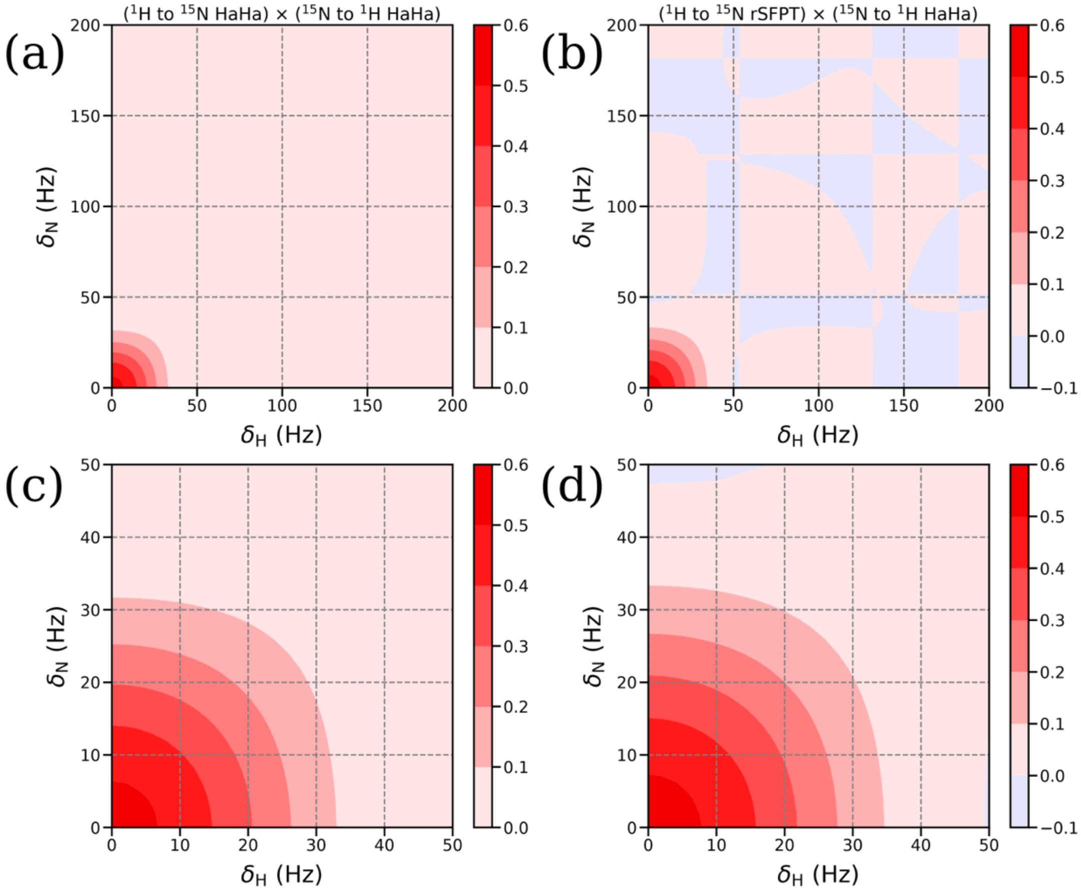

Figure S2. Simulated H_x magnetization as a function of ^{1}H and ^{15}N offset after ^{1}H→^{15}N selective HaHa (a,c) and rSFPT (b,d) followed by a ^{15}N→^{1}H selective HaHa, starting from H_x magnetization. J_{NH} is assumed –94 Hz. Offsets in the range of 0 to 200 Hz are shown in panels C and D show the range 0 to 50 Hz. The effects of relaxation were taken into account in this simulation, with ^{15}N R_2 = 10 s^{-1}, $^1H^N$ R_2 = 46.6 s^{-1}, while ^{15}N and $^1H^N$ R_1 = 2 s^{-1}.

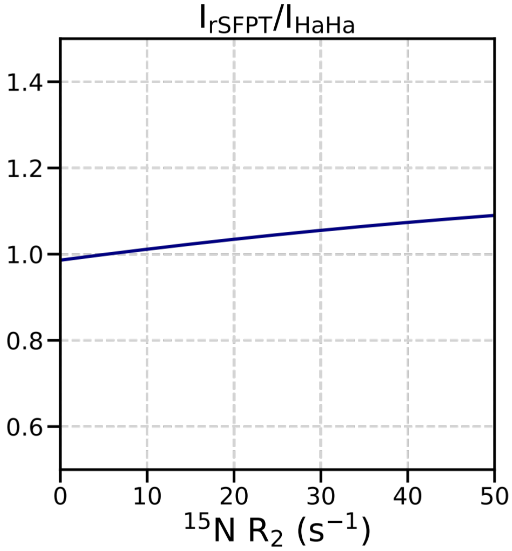

Figure S3. Ratio between simulated output on-resonance magnetizations N_x of rSFPT and HaHa elements as a function of ^{15}N R_2 rates. The assumed other relaxation rates are: $^1H^N$ R_2 = 4.66 × ^{15}N R_2, ^{15}N and $^1H^N$ R_1 = 2 s^{-1}.

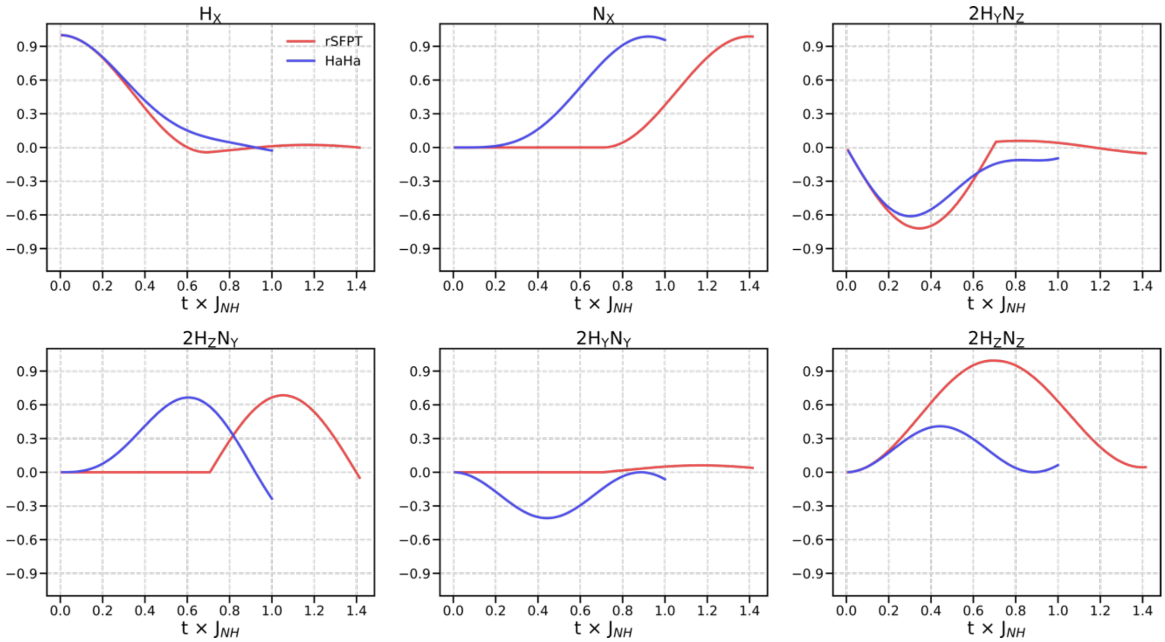

Figure S4. Simulated evolution of product operator terms during ^{1}H SFPT and HaHa, starting from H_x, using a B_1 field of 45 Hz on ^{1}H and [15]N (*rf*-field phases: –x) as a function of time expressed as a fraction of $^1J_{NH}$ (assumed to be –94 Hz). The total duration of rSFPT is $\sqrt{2}(J_{NH})^{-1}$ and the one of HaHa is $(J_{NH})^{-1}$. Terms that for both rSFPT and HaHa remain near-zero (< 0.01) during the whole sequence are not shown. The effects of relaxation were not taken into account.

2.2. ^{15}N Chemical Shift Selective Filter

2.2.1. General principles

The ^{15}N selectivity of the selective in-phase polarization transfer remains insufficient in many cases. We therefore implemented a ^{15}N chemical shift selective filter (CSSF).

To avoid the consequences of $^1J_{HN}$ phase evolution and the splitting of the ^{15}N frequency during this filter, ^{1}H decoupling (spin-lock) is applied during the CSSF using a simple continuous wave (CW) irradiation of 8 kHz. The ^{1}H magnetization before and after the ^{1}H spin-lock is first aligned using a pulse-delay scheme (see Figure 4 in the main manuscript), with the χ delay set to $(1/\omega_{SL})$-$(4p_{90}/\pi)$), with ω_{SL} the spin-lock *rf*-field strength and p_{90} the duration of the ^{1}H 90° hard pulse. This scheme allows for the magnetizations of both the selected on-resonance amide proton and the off-resonance water protons to be aligned with their respective effective B_1 fields experienced during the spin-lock. This should mitigate the complications from radiation damping coming from the water signal.[23, 24]

The ^{15}N CSSF is based on a $90°_y$ - ζ - $90°_{-y}$ scheme, as previously proposed by Korzhnev *et al.*[17] Whereas on-resonance ^{15}N *z*-magnetization will perfectly survive this scheme, responses that are 1/(4ζ) Hz off-resonance should be fully suppressed thanks to a pulsed field gradient (g_3) immediately following the $90°_{-y}$ pulse. However, application of ζ-delay longer than 5 ms (corresponding to 50 Hz off-resonance) leads to significant ^{15}N transverse relaxation losses. Furthermore, any nearby responses closer or farther than 1/(4ζ) Hz will not be perfectly removed, making this basic strategy insufficient for extremely crowded spectra, as observed previously.[25]

As an alternative strategy, we resorted to replacing the first ^{15}N 90° pulse by a selective pulse, as outlined in the following section.

2.2.2. Choice of a 90° selective pulse shape

We explored four different pulse shapes: Sinc,[12] eSNOB$_{270}$,[13] HalfGauss,[14, 26] and the new GOLEM shape (Figure S5).

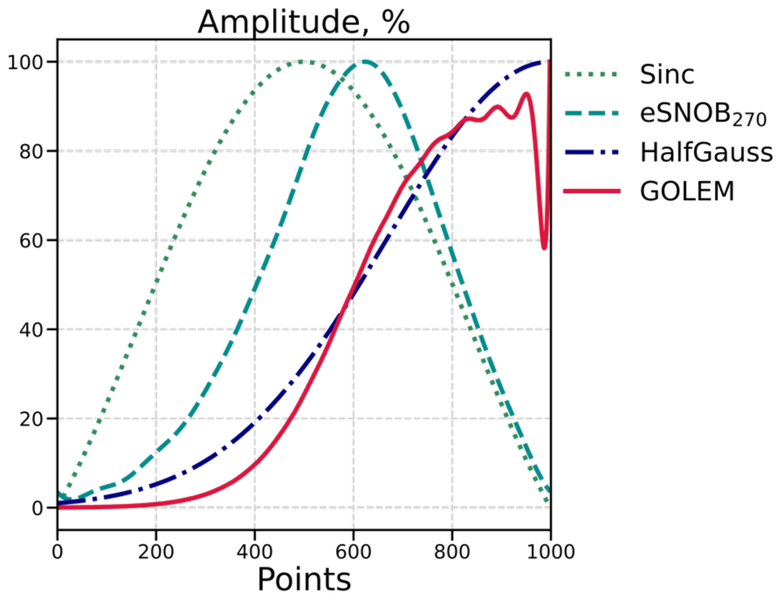

Figure S5. Overlay of the amplitudes of Sinc, eSNOB$_{270}$, HalfGauss and GOLEM pulse shapes. The phase stays constant for each pulse.

Figure S6 compares the resulting magnetization components (M_x, M_y and M_z) after 90° excitation (100 ms pulse length with y-phase) as a function of offset of the applied pulse. Within the context of the CSSF, only the profile of the M_x component is relevant, as M_y will be purged.

The Sinc pulse (here implemented as the standard Bruker Sinc1.1000 shape only featuring the central bell-shaped portion) features very significant negative side-bands in M_x, which would create undesirable off-resonance excitation. For Sinc pulses longer than ca. 15 ms, these negative side-bands fall within the HaHa or rSFPT ^{15}N excitation bandwidth and thus will interfere with the overall SNIPER selectivity.

The eSNOB$_{270}$ shape does yield a clean positive excitation profile in M_x. Unfortunately, it requires much longer pulse lengths than the Sinc shape in order to obtain a similar excitation bandwidth. The penalty on sensitivity from T_2 relaxation weighting is therefore severe.

The HalfGauss pulse equally provides a clean positive excitation profile in M_x. Because the rf-amplitude only gradually increases during the pulse with the maximum reached at the end, on-resonance magnetization gains its transverse character mostly at a late stage. This translates into a much-reduced T_2 loss compared to Sinc or eSNOB$_{270}$ pulses of equal length. The M_x excitation profile is sharper than that of the eSNOB$_{270}$ shape at the expense of a much broader, dispersive M_y component.[14, 26] Since only the M_x component survives the CSSF, the broad M_y component is of no consequence, meaning the HalfGauss shape is always a better choice than eSNOB$_{270}$ in this context.

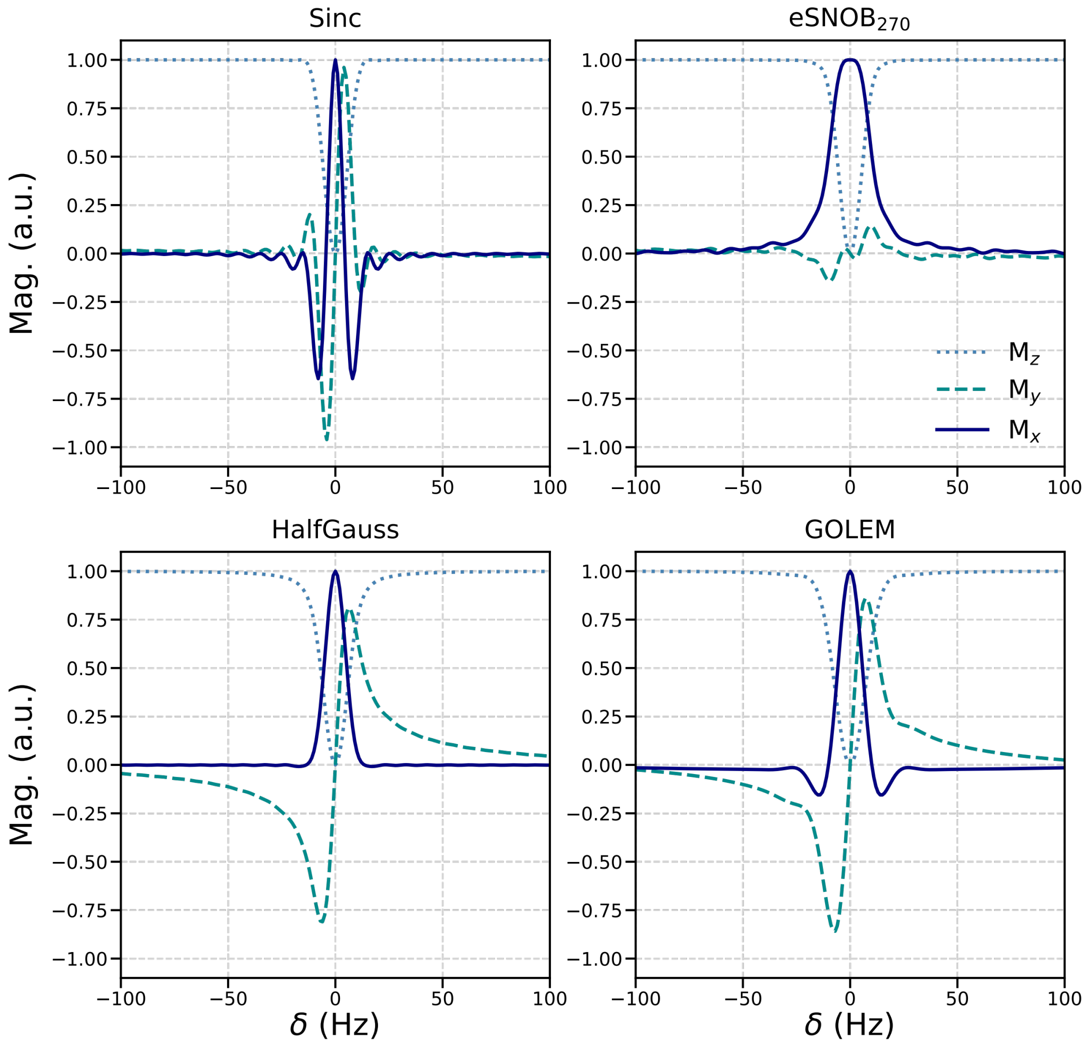

Figure S6. Excitation profiles of 100 ms Sinc, eSNOB$_{270}$, HalfGauss and GOLEM selective pulses with y-phase.

We found the HalfGauss shape to be a better choice than the single-lobed Sinc pulse when a selectivity lower 27 Hz was needed. However, the tails of its Gaussian-shaped excitation profile are an important point of attention. If this hits a nearby off-resonance ^{15}N resonance, it will contribute to the detected SNIPER ^{1}H signal. Even if this accounts for just 10% of the total signal intensity, it can be detrimental for the accuracy and precision of the relaxation measurement. For the huntingtin exon-1 protein, this became problematic when ^{15}N frequency differences were less than ca. 12 Hz. The HalfGauss pulse durations needed to avoid contributions from off-resonance correlations resulted in unacceptably high R_2 relaxation losses. The Q18 and Q20 correlations present an example of such problematic case: they have nearly identical ^{1}H chemical shifts, while their peak centers are merely 8 Hz apart along the ^{15}N dimension. In addition, they also feature the highest ^{15}N R_2 values of the whole polyQ stretch, meaning frugality is needed for selective pulse lengths. A HalfGauss pulse duration of 150 ms set to Q20 was found to be the longest that could be afforded in terms of signal-to-noise ratio, but unfortunately this still yielded roughly 15% of the Q18 signal (see below, Figure S9).

Using optimal control, we searched for a pulse shape with reduced tails in the excitation profile. As target excitation profile, we chose the one from the Sinc shape but with zeroed intensity at and beyond the off-resonance negative side-lobes of the M_x component. The resulting GOLEM shape turned out very similar to the HalfGauss shape, albeit with a somewhat steeper rise in

rf-amplitude and some sharp features towards the end. GOLEM yields an M_x excitation profile that still contains some residual negative side-lobes, though with much reduced intensity compared to the Sinc one.

For very long selective pulses and when $R_2 >> R_1$, the effects of relaxation during the pulse on the resulting M_x excitation profile are very significant and should be taken into account.[27] Simulations show that when R_2 rates of ca. 10 s^{-1} are assumed for pulses longer than 150 ms, GOLEM's negative side-lobe intensities in the M_x excitation profile are strongly diminished relative to the on-resonance intensity (Figure S7), whereas the tails of the HalfGauss profile become more pronounced. Overall, at such long pulse lengths, GOLEM yields acceptable relative intensities for the side-lobes and with much reduced contributions at the tail of the excitation profile compared to the HalfGauss shape (Figure S8).

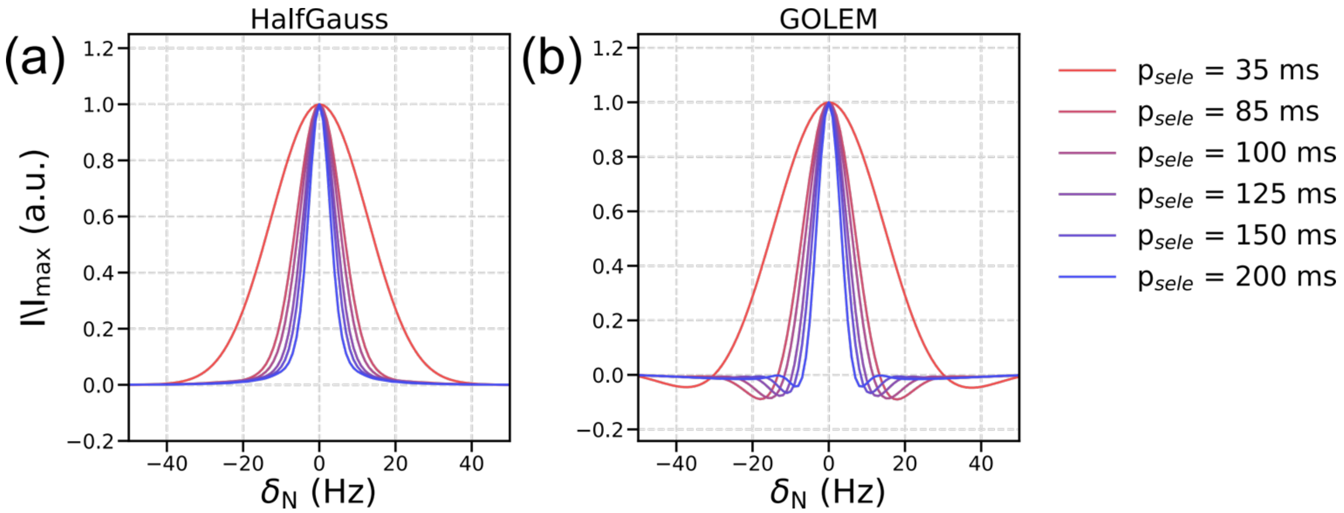

Figure S7. Simulated SNIPER intensities as a function of ^{15}N frequency offset, resulting from the multiplied excitation profiles of a rSFPT $^{1}H \rightarrow ^{15}N$ polarization transfer and the ^{15}N CSSF using either a HalfGauss (a) or a GOLEM (b) shaped pulse. Intensity profiles for various shaped pulse lengths (p_{sele}) are shown. The effects of relaxation were taken into account in this simulation, with R_2 = 10 s^{-1} and R_1 = 2 s^{-1}. The ^{15}N on-resonance intensity was normalized to 1.0, meaning the relaxation weighting on-resonance is not visible in this figure.

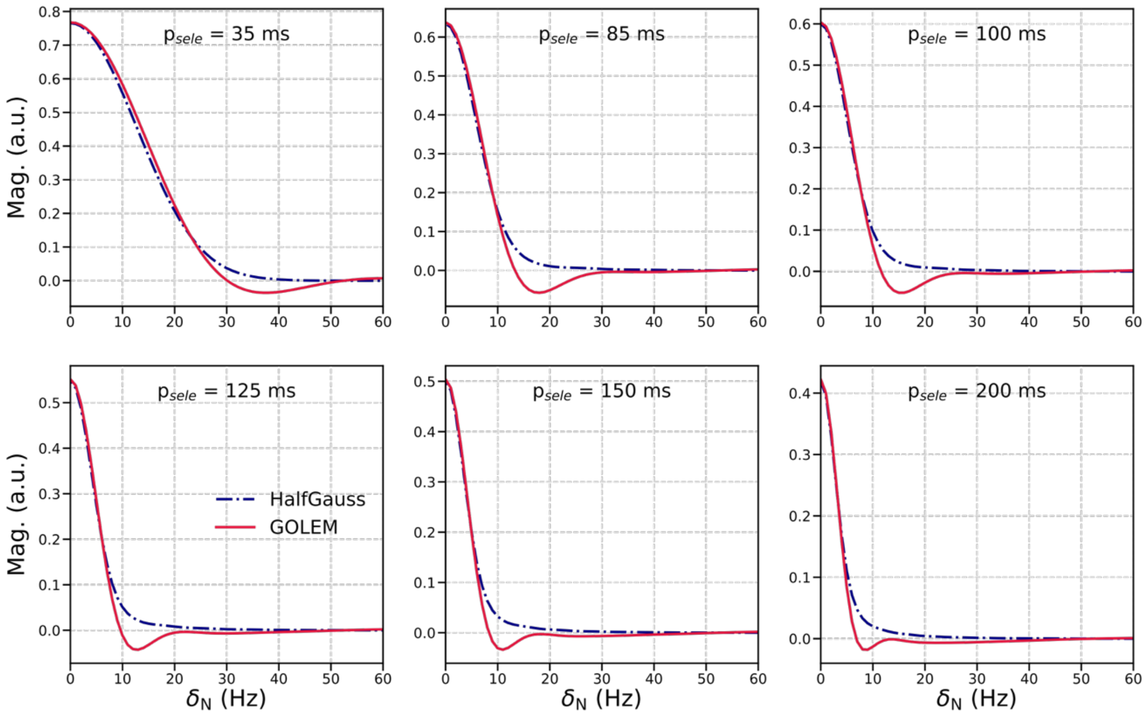

Figure S8. Same as Figure S7, but with the HalfGauss (blue) and GOLEM (red) intensity profiles overlayed for each pulse length. On-resonance, the HalfGauss and GOLEM shapes showed nearly identical relaxation losses, with obtained on-resonance intensity ratios HalfGauss:GOLEM of 0.997 (35 ms), 0.994 (85 ms), 0.994 (100 ms), 0.993 (125 ms), 0.993 (150 ms) and 0.993 (200 ms).

The GOLEM shape becomes most useful in cases when ^{1}H-^{15}N correlations have both extremely small ^{15}N frequency differences and similar ^{1}H chemical shift. This is illustrated experimentally using a 2D SNIPER experiment, selecting for Q20 in htt-exon1 and using either a 150 ms HalfGauss or 150 ms GOLEM shaped pulse. The 1D spectrum resulting from the first t_1-increment (which is equivalent to 1D SNIPER) shows a 15% higher maximum intensity for the HalfGauss experiment relative to the GOLEM experiment (Figure S9). Yet, our simulations (Figure S8) showed the on-resonance relaxation weighting to be identical for both pulses. Taking the ^{15}N cross-sections along the 2D ^{1}H-^{15}N correlation in the 2D SNIPER spectra reveals that the maximum intensities in HalfGauss and GOLEM are similar. However, in the HalfGauss experiment, the signal shows a slight shoulder towards the Q18 ^{15}N chemical shift. This is absent in the GOLEM experiment. This explains why the first increment of the SNIPER experiment has a slightly higher intensity when using HalfGauss: it has a small but significant residual contribution from Q18, which would be harmful for relaxation analysis.

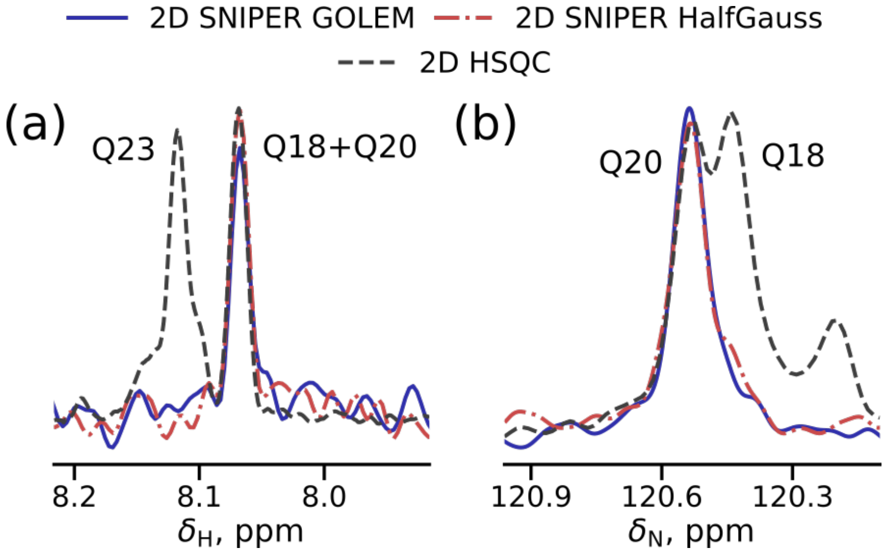

Figure S9. Results from 2D SNIPER experiments applied on Q20 of htt-exon-1, using either a 150 ms HalfGauss or a 150 ms GOLEM shaped pulse in the ^{15}N CSSF. (a) Overlayed 1D spectra resulting from the first t_1-increments of the 2D SNIPER experiments. (b) ^{15}N cross-sections taken at the Q20 ^{1}H frequency. The grey dashed lines are the ^{1}H or ^{15}N cross-sections taken from the standard ^{1}H-^{15}N HSQC for comparison, with intensities scaled to match the cross-section of SNIPER using HalfGauss.

In summary, the optimal choice of the selective pulse shape depends on the required overall ^{15}N selectivity. Figure S10 simulates the ^{15}N excitation profiles as a function of selective pulse length for all four shapes assuming the combined effects of the HaHa or rSFPT element and the CSSF (ζ = 0) and including the impact of relaxation. When correlations are separated by more than 27-50 Hz, the single-lobed Sinc pulse can be used as the negative side-lobes are mostly suppressed by the rSFPT element. Generally, the Sinc pulse achieves similar excitation bandwidths (excluding the negative slide-lobes) for a significantly shorter pulse length than the HalfGauss; meaning less relaxation losses. When selective bandwidths between 12 and 27 Hz are needed, the negative side-bands of the Sinc pulse do come through, and the HalfGauss shape needs to be used. For less than 12 Hz peak separation, the use of the GOLEM pulse shape is advised (see section 6.1).

To review the overall ^{1}H and ^{15}N SNIPER selectivity when using very long ^{15}N selective pulses, Figure S11 simulates the excitation profiles for a SNIPER sequence using rSFPT for ^{1}H→^{15}N transfer, a CSSF (ζ = 0) using a GOLEM 100 ms selective pulse, and a selective HaHa element for ^{15}N→^{1}H transfer. It can be observed that the ^{15}N selection bandwidth is significantly narrower than the ^{1}H bandwidth, the latter only determined by the selective polarization transfer.

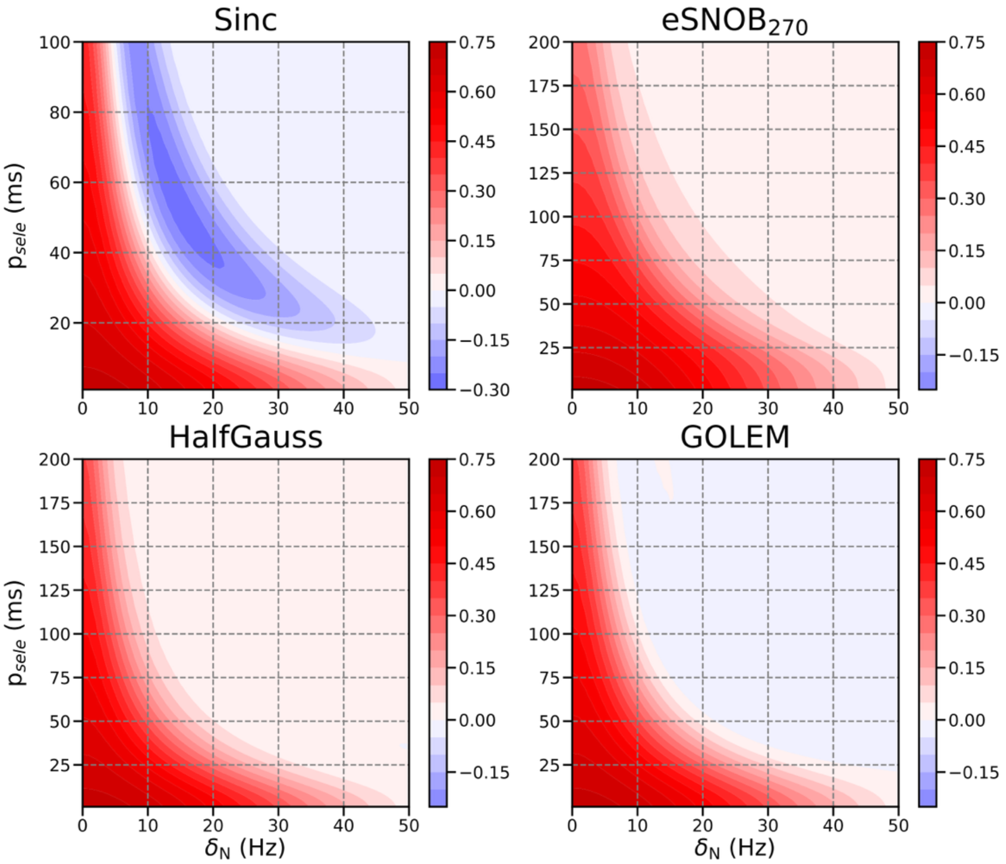

Figure S10. Magnetization intensity as a function of ^{15}N offset and shaped pulse length, obtained from multiplying the profiles simulated for $^{1}H \rightarrow ^{15}N$ rSFPT, the ^{15}N CSSF ($\zeta = 0$) using either Sinc, eSNOB270, HalfGauss or GOLEM pulse shapes, and a $^{15}N \rightarrow ^{1}H$ selective HaHa. J_{NH} is assumed –94 Hz. The effects of relaxation were taken into account in this simulation, with ^{15}N $R_2 = 10$ s^{-1}, $^{1}H^{N}$ $R_2 = 46.6$ s^{-1}, while ^{15}N and $^{1}H^{N}$ $R_1 = 2$ s^{-1}.

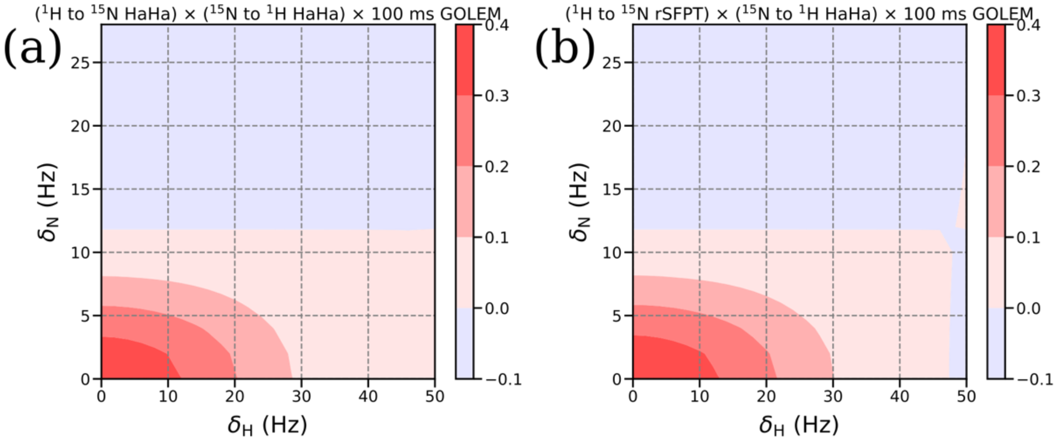

Figure S11. (A) Magnetization intensity as a function of ^{1}H and ^{15}N offset, obtained from multiplying the profiles simulated for $^{1}H \rightarrow ^{15}N$ HaHa, the ^{15}N CSSF ($\zeta = 0$) using a 100ms GOLEM shape, and $^{15}N \rightarrow ^{1}H$ selective HaHa. (B) Same as in (A), but using a $^{1}H \rightarrow ^{15}N$ rSFPT instead. J_{NH} is assumed –94 Hz. The effects of relaxation were taken into account in this simulation, with ^{15}N $R_2 = 10$ s^{-1}, $^{1}H^{N}$ $R_2 = 46.6$ s^{-1}, while ^{15}N and $^{1}H^{N}$ $R_1 = 2$ s^{-1}.

2.3. Optional ^{1}H selective filter

An optional ^{1}H chemical shift selective filter (^{1}H CSSF) is included in the SNIPER pulse sequence. It may aid in some cases to remove nearby spurious ^{1}H resonances coming from ^{1}H-^{15}N correlations with similar ^{15}N chemical shift, although in practice it does not add significantly to the ^{1}H selectivity of the selective polarization transfer steps. A scheme combining ^{1}H 90° hard pulses, a ^{1}H 90° selective pulse and a pulsed field gradient is applied to dephase all off-resonance ^{1}H magnetization. During this, ^{15}N CW_x-decoupling is applied (N_z magnetization is first converted to N_x before the CW_x-decoupling and afterwards converted back to N_z). Such scheme is either applied as initial ^{1}H excitation in case of the SNIPER R_1, $R_{1\rho}$ and *zz*-exchange experiment, or just before the WATERGATE element in case of the SNIPER nOe experiment. Although this scheme was applied in some cases for R_1, $R_{1\rho}$ measurements of the huntingtin exon-1 protein, in hindsight, it was not crucial.

2.4. R_1 relaxation and *zz*-exchange block

For SNIPER R_1 and *zz*-exchange experiments (Figure S12A), the ^{15}N R_1 relaxation encoding block contains selective $^1H^N$ inversion pulses applied on-resonance in such a way that there is no excitation of the water resonance in order to suppress the effects of ^{15}N chemical shift anisotropy – ^{1}H-^{15}N dipole-dipole (CSA-DD) cross-correlated relaxation. Similarly, if the protein is ^{13}C labelled, hard π pulses with offsets on ^{13}Cα or ^{13}C' are applied to suppress ^{15}N - ^{13}C CSA-DD cross-correlated relaxation (involving either ^{13}Cα or ^{13}C').

Note that only one selective polarization transfer (^{1}H→^{15}N) is applied in the *zz*-exchange experiment, meaning the ^{1}H selectivity is as shown in Figure S1 rather than as in Figure S2. The ^{15}N selectivity is determined mostly by the ^{15}N CSSF when using long selective pulses.

2.5. $R_{1\rho}$ relaxation block

In the SNIPER $R_{1\rho}$ experiment, a constant time ^{15}N {$R_{1\rho}$-R_1} relaxation encoding block is used. A ^{1}H spin-lock (CW_x, 8 kHz) is applied on-resonance to the target $^1H^N$ resonance, using the same scheme as used in the ^{15}N CSSF (*vide supra*) to avoid radiation damping by the water signal.[23, 24] As a single ^{15}N frequency is selected, the ^{15}N magnetization is readily aligned with the off-resonance spin-lock (SL) using ^{15}N 90° hard pulses (with phases φ_4 and φ_5), as has been proposed earlier.[17]

Temperature compensation is an absolute requirement in $R_{1\rho}$ experiments. Temperature compensation spin-locks are usually implemented during the interscan delay.[28] On our spectrometers, we found a very significant improvement in temperature stability when we instead applied the temperature compensation spin-locks within the constant-time relaxation blocks, flanking the main ^{15}N spin-lock. These additional temperature compensation spin-locks (T) are applied at the same power level as the main ^{15}N spin-lock, but far off-resonance, at approximately -600 to -900 ppm away from the resonance of interest.

2.6. ^{1}H-^{15}N nOe SNIPER sequence

The SNIPER {^{1}H}-^{15}N heteronuclear nOe pulse sequence is shown in Figure S12B. The initial ^{1}H magnetization is either saturated by a train of n ^{1}H π hard pulses interspersed by delays $2\delta = 2(J_{NH})^{-1}$ or left at equilibrium. The required duration of ^{1}H saturation has to be long enough to achieve complete steady-state ^{15}N N_z magnetization and depends on the ^{15}N R_1 rates. It is controlled by the loop counter n, which in our work is in the range of 230-370 (total saturation/recovery delay of 5 to 8 seconds). After ^{15}N excitation, the ^{15}N CSSF is applied, and the ^{15}N→^{1}H polarization transfer is achieved using a rSFPT element. An optional ^{1}H CSSF and the WATERGATE[3] solvent suppression scheme is applied before readout.

Note that only one selective polarization transfer (^{15}N→^{1}H) is applied in this experiment, meaning the ^{1}H selectivity is as shown in Figure S1 rather than as in Figure S2. The ^{15}N selectivity is determined mostly by the ^{15}N CSSF when using long selective pulses.

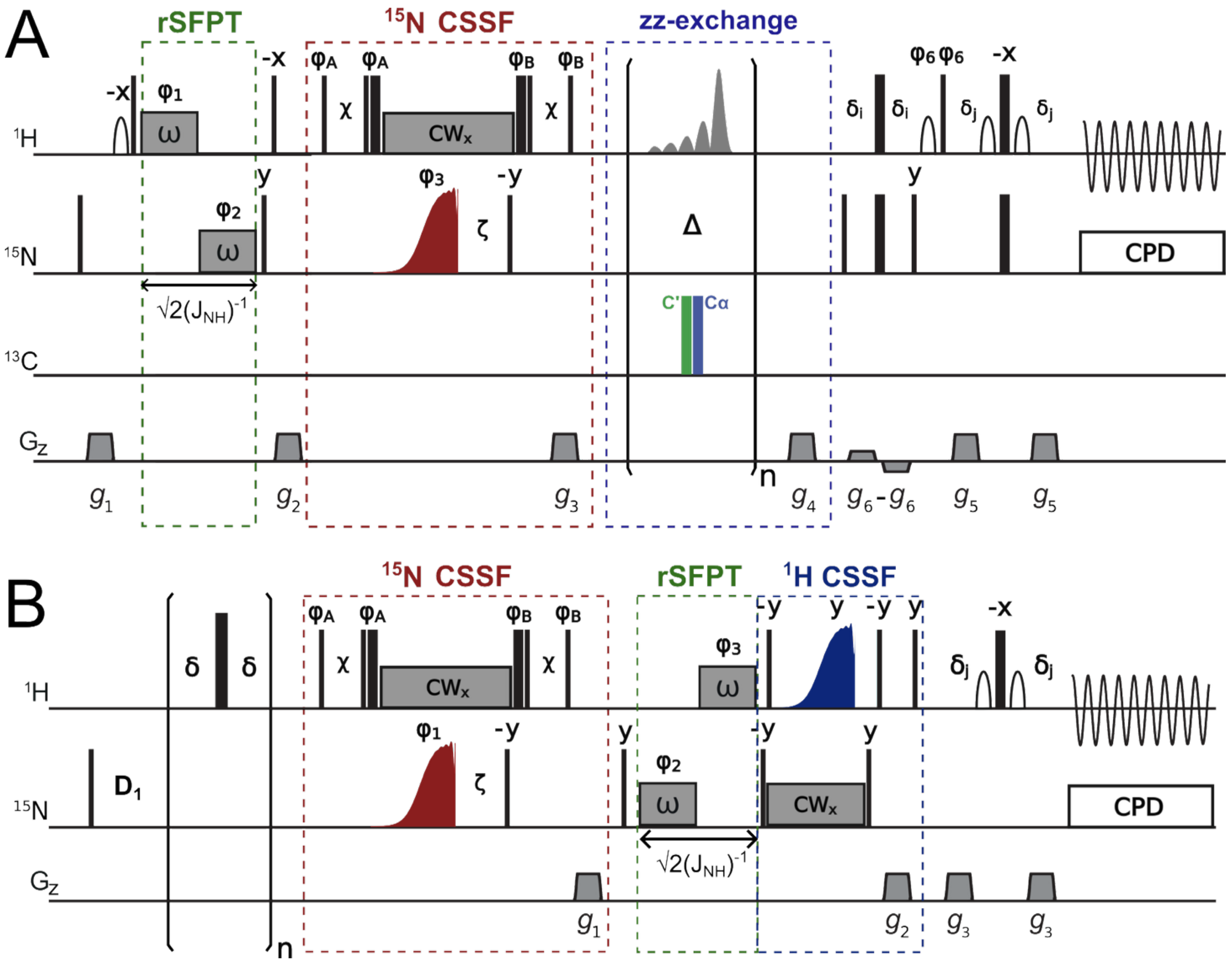

Figure S12. Pulse sequences for (A) *zz*-exchange SNIPER and (B) SNIPER {^{1}H}-^{15}N nOe. δ_i is $(4J_{NH})^{-1}$ (2.65 ms), δ_j is 2.35 ms. The $^{13}C^{\alpha}$ and ^{13}C' pulses are 180° pulses.

2.7. Phase cycle and gradient strengths

Table S1: Phase cycle for SNIPER R_1 and $R_{1\rho}$

Phase label	Phase cycle (written as multipliers of 90°)
φ_1	13
φ_2	$2_2 0_2$
φ_3	$0_4 2_4$
φ_4	1 for positive SL-offsets, 3 for negative SL-offsets
φ_5	3 for positive SL-offsets, 1 for negative SL-offsets
φ_7	$0_8 2_8$
φ_8	$0_{16} 2_{16}$
φ_A	$1_2 3_2$
φ_B	$3_2 1_2$
φ_R	$\varphi_1 + \varphi_2 + \varphi_3 + \varphi_7 + \varphi_8$

Table S2: Phase cycle for SNIPER *zz*-exchange

Phase label	Phase cycle (written as multipliers of 90°)
φ_1	13
φ_2	$2_2 0_2$
φ_3	$0_4 2_4$
φ_6	$0_8 2_8$
φ_A	$1_2 3_2$
φ_B	$3_2 1_2$
φ_R	$\varphi_1 + \varphi_2 + \varphi_3 + \varphi_6$

Table S3: Phase cycle for SNIPER nOe

Phase label	Phase cycle (written as multipliers of 90°)
φ_1	$0_4 2_4$
φ_2	$0_2 2_2$
φ_3	02
φ_A	$1_2 3_2$
φ_B	$3_2 1_2$
φ_R	$\varphi_1 + \varphi_2 + \varphi_3$

Table S4: Pulsed Field Gradient amplitudes and durations used in SNIPER R_1, $R_{1\rho}$ and *zz*-exchange

Gradient label	Amplitude ($G.cm^{-1}$)	Duration (ms)
g_1	34.3	1.0
g_2	–39.8	1.0
g_3	–49.6	1.0
g_4	28	1.0
g_5	1.6	2.65
g_6	45.3	0.5

Table S5: Pulsed Field Gradient amplitudes and durations used in SNIPER nOe

Gradient label	Amplitude ($G.cm^{-1}$)	Duration (ms)
g_1	39	1.0
g_2	32	0.5
g_3	25	0.5

3. Signal-to-noise ratio and time-gain of 1D SNIPER

We compare the signal-to-noise ratios (SNR) of 1D SNIPER and the standard 2D approach using the huntingtin exon-1 protein. Residue Q18 is merely 8 Hz away from Q20, its nearest neighbor along F_1 and with a near identical ^{1}H chemical shift (see Figure 1 of the main paper). A 2D ^{1}H-^{15}N HSQC for R_1 measurement[28] with a 0 ms relaxation encoding delay was recorded 14 ppm ^{15}N spectral window, 4 transients and 1.2 s recycle delay. The first 512 total t_1 time domain points took 54 minutes. This results in a digital resolution of 2.50 Hz along the ^{15}N dimension. From the F_1-cross-section shown in Figure S13, such high digital resolution – combined with a resolution-enhancing Lorenz-to-Gauss apodization, at the cost of SNR – is critical to resolve the Q18 and Q20 signals (a similar illustration is made for Q48/Q59 in the Figure). The SNR measured on the ^{1}H F_2-cross-section taken at this Q18 peak was 68.2.

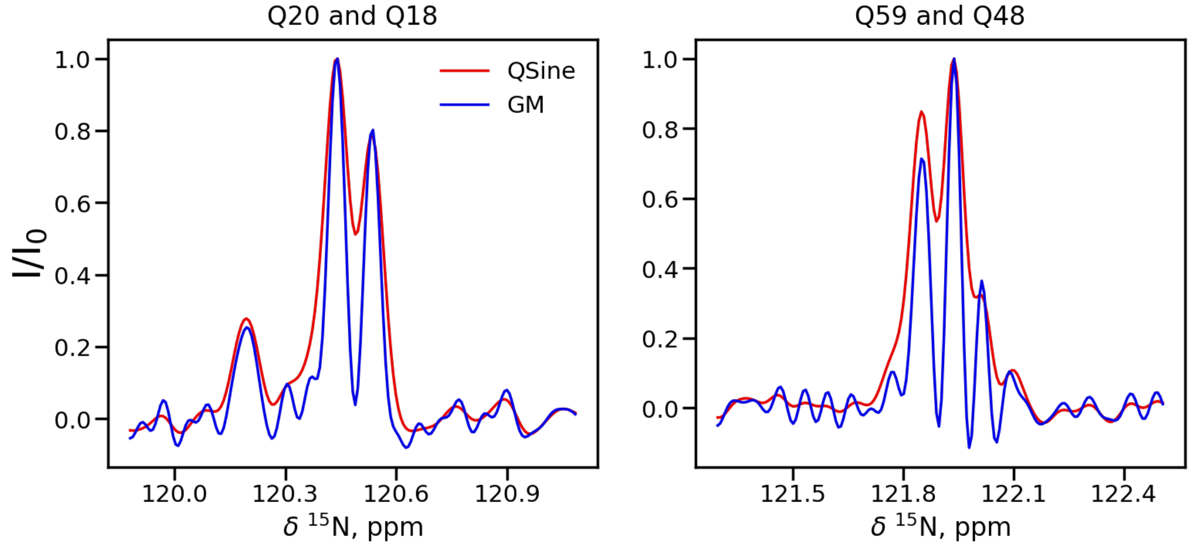

Figure S13. ^{15}N cross-sections at the ^{1}H chemical shift of Q20/Q18 (left) and Q59/Q48 (right) of a 2D R_1 experiments with 0 ms relaxation encoding delay at the ^{1}H offset of overlapped resonances. Spectra were processed using 512 total t_1 time domain points, resulting in digital resolutions of 2.50 Hz in F_1. Squared cosine bell (QSINE, red) or Lorentz-to-Gauss apodization (blue) has been used in F_1, with the latter using –5 Hz line broadening factor and Gaussian maximum at 0.6. Zero filling in F_1 was used to reach 4096 real time domain points.

Q18's proximity to Q20 means SNIPER requires a long ^{15}N selective pulse in the ^{15}N CSSF for this residue (GOLEM 150 ms). It is also one of the fastest relaxing ones in the polyQ (^{15}N R_2 = 8.6 s^{-1}). A 1D SNIPER R_1 set to Q18 was recorded with a 0 ms relaxation encoding delay, 512 transients and 1.0 s recycle delay, taking 12.58 minutes. The SNR was 56.1.Thus, despite the significant relaxation losses during the rSFPT and ^{15}N CSSF elements, SNIPER achieves a cleanly resolved signal of this individual residue in just a fraction of the time that would be needed by the standard 2D experiment and at a very reasonable cost in SNR. Of course, for correlations that are well-resolved in the standard 2D experiment with reasonable minimal experimental times, the non-selective 2D approach remains the most time-efficient method, as it delivers all such correlations in one experiment. SNIPER should be used as a complementary experiment on the heavily overlapped correlations.

The main advantage of selective 1D measurements is the shorter minimal experimental time: one can compromise in SNR by reducing the number of transients while retaining the same resolving power. The standard 2D approach is limited by the number of time-increments required to attain sufficient digital resolution in the ^{15}N dimension and the minimum transients needed for phase cycling (typically 4). We therefore wished to verify whether 1D SNIPER can

reliably extract relaxation rates at significantly lower SNRs than those achieved in the initial set of experiments from which the rates presented in the main manuscript were obtained (see Table S8 in section 4 for those SNRs). For this, a second huntingtin sample was prepared with different conditions (340 μM, pH = 7.6). We re-measured some R_1 and $R_{1\rho}$ rates using 1D SNIPER for a selected number of glutamines with a reduced number of transients, leading to significantly lower SNRs (Table S6).

To partly compensate for the loss in SNR, we decided to use an alternative sampling of the intensity attenuation curve that is optimized per-residue. This is something that cannot be achieved using the non-selective 2D approach, which has to accommodate a range of relaxation rates. Jones *et al.* have shown that, when considering just a single exponential decay with decay rate R, the sampling scheme leading to the narrowest confidence intervals requires sampling at just two time-points: at t = 0 and at t = 1.30/R, the latter corresponding to a signal attenuation of ca. 0.27 compared to the former.[29] However, four times more measurements should be performed at the latter time-point than at the former. The only information that is lost compared to an evenly spread sampling across the decay is that it is no longer possible to observe that the intensity decay indeed adheres to a single exponential, but this is an assumption that is usually met in absence of signal overlap and correct experimental setup. Thanks to the fast 1D measurement, the relaxation weighting delay needed for a signal attenuation of 0.27 can be very quickly screened in ca. 5-10 minutes using a minimal number of scans.

In this way, 20 1D SNIPER R_1 and/or $R_{1\rho}$ spectra were measured per residue: 4 spectra at t = 0 and 16 spectra at t = *ca.* 1.30/R, each with a number of transients sufficient to obtain a signal-to-noise ratio between 15 and 30 at t = 0. Data fitting was performed using all 20 data points, as well as on two subsets formed by splitting the data into two groups of 10 spectra (2 at t = 0 and 8 at t = *ca.* 1.30/R). The results are shown in Table S7, while the fitted curves are shown in Figures S14 and S15.

The error estimates of the relaxation values (obtained using a Monte Carlo procedure[30]) are very reasonable, corresponding to relative errors of at most 5-7%. The rates obtained using the two subsets of 10 points have reasonable confidence intervals and do not differ more from each other than the confidence intervals predict. The obtained relaxation rates do slightly differ from those obtained from the first huntingtin sample (Table S9), which can be attributed to the higher pH. These results show that even with lower SNRs, SNIPER R_1 and $R_{1\rho}$ experiments can deliver data of sufficient quality when using optimal sampling schemes.

If one assumes a minimum of 4 transients for phase cycling in the 2D approach, a single data points would take about 1 hour, but in practice more transients would likely be needed to compensate for the low signal-to-noise ratio and suboptimal data sampling. The data points for the 1D SNIPERs acquired here took between 10 and 30 minutes depending on the residue.

Table S6. 1D SNIPER datasets recorded on huntingtin exon-1, pH = 7.5, using Jones' time-sampling method.

Residue	Type	^{1}H CSSF pulse		^{15}N CSSF			N° of transients	Exp. time for a single time-point (h)	Signal-to-noise Ratio*
		Pulse Shape	Sel. Pulse Length (ms)	Pulse Shape	Sel. Pulse Length (ms)	ζ-delay (ms)			
Q19	$R_{1\rho}$	HalfGauss	0.1	GOLEM	100	0	480	0.22	28.04
Q19	R_1	HalfGauss	0.1	GOLEM	100	0	256	0.17	30.53
Q20	$R_{1\rho}$	HalfGauss	0.1	GOLEM	150	0	640	0.32	17.19
Q20	R_1	HalfGauss	0.1	GOLEM	150	0	352	0.24	17.33
Q25	$R_{1\rho}$	HalfGauss	0.1	GOLEM	150	0	640	0.32	16.32
Q25	R_1	HalfGauss	0.1	GOLEM	150	0	352	0.24	22.25
Q30	$R_{1\rho}$	HalfGauss	0.1	HalfGauss	80	0	480	0.25	15.93
Q30	R_1	HalfGauss	0.1	HalfGauss	80	0	256	0.17	24.06
Q32	$R_{1\rho}$	HalfGauss	0.1	HalfGauss	30	0	480	0.24	17.51
Q32	R_1	HalfGauss	0.1	HalfGauss	30	0	256	0.17	28.13
Q54	$R_{1\rho}$	HalfGauss	0.1	GOLEM	100	0	320	0.20	21.42
Q54	$R_{1\rho}$	HalfGauss	0.1	GOLEM	100	0	160	0.11	51.5
Q59	R_1	HalfGauss	0.1	GOLEM	150	0	352	0.19	24.89
Q59	$R_{1\rho}$	HalfGauss	0.1	GOLEM	150	0	160	0.11	28.26
Q61	R_1	HalfGauss	0.1	HalfGauss	1	0	192	0.09	32.85
Q61	$R_{1\rho}$	HalfGauss	0.1	HalfGauss	1	0	128	0.08	41.52

* SNRs are taken from the 1D SNIPER R_1 and $R_{1\rho}$ experiments with zero relaxation encoding delay.

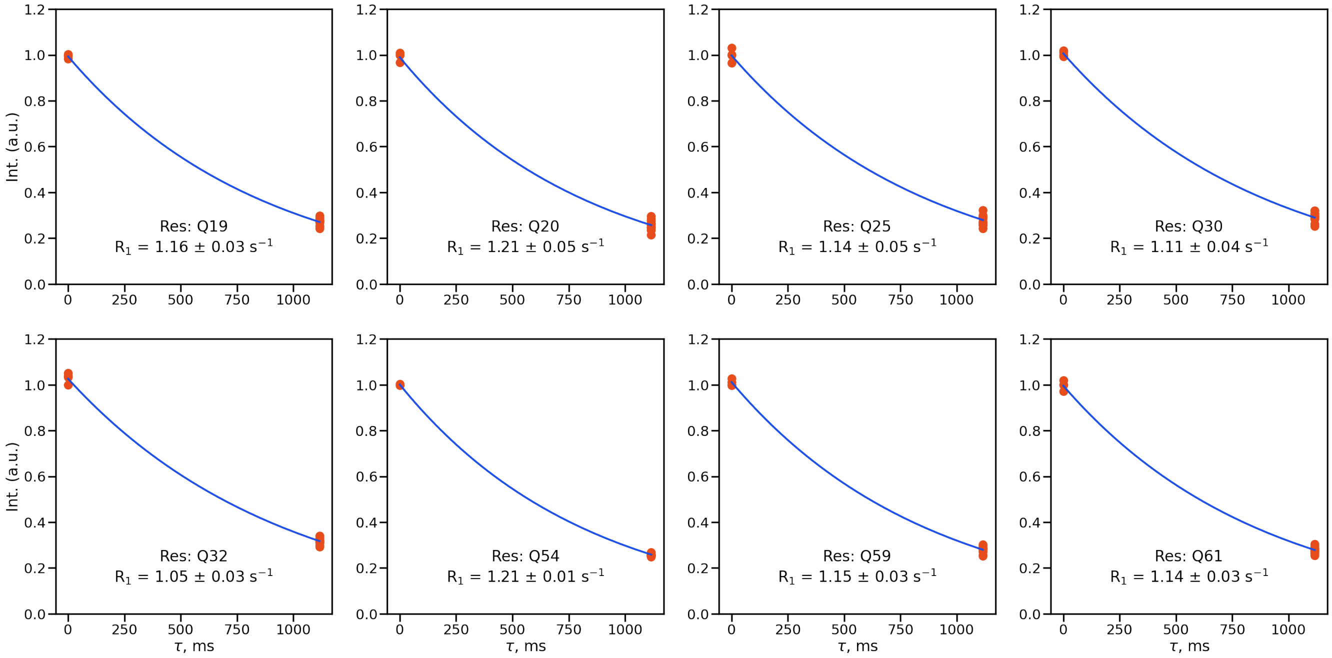

Figure S14. Experimental (red points, using all 20 acquired data points) and fitted (blue line) intensity attenuations from 1D SNIPER R_1 data sets acquired on huntingtin exon 1 (pH 7.5), using Jones' optimized sampling scheme.

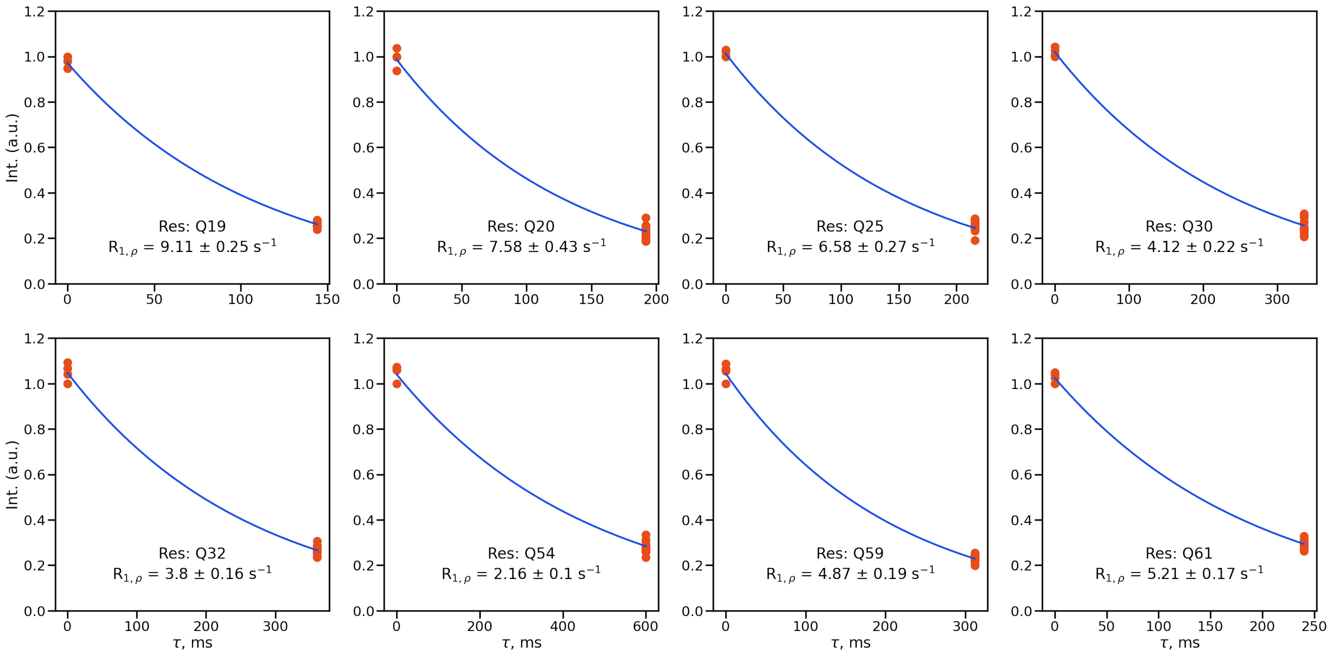

Figure S15. Experimental (red points, using all 20 acquired data points) and fitted (blue line) intensity attenuations from 1D SNIPER on-resonance (constant-time) $R_{1\rho}$ data sets acquired on huntingtin exon 1 (pH 7.5), using Jones' optimized sampling scheme.

Table S7. Fitted ^{15}N R_1 and ^{15}N $R_{1\rho}$ values and 95% error of fitting estimates for huntingtin exon-1, pH = 7.5, using Jones' time-sampling method.

Residue	Type	R (s^{-1}) 20 points	ΔR (s^{-1}) 20 points	R (s^{-1}) 10 points (subset 1)	ΔR (s^{-1}) 10 points (subset 1)	R (s^{-1}) 10 points (subset 2)	ΔR (s^{-1}) 10 points (subset 2)
Q19	$R_{1\rho}$	9.111	0.250	9.155	0.294	9.249	0.326
Q19	R_1	1.165	0.031	1.164	0.046	1.157	0.038
Q20	$R_{1\rho}$	7.580	0.434	7.513	0.426	7.301	0.592
Q20	R_1	1.208	0.045	1.175	0.069	1.220	0.070
Q25	$R_{1\rho}$	6.579	0.266	6.599	0.444	6.362	0.205
Q25	R_1	1.141	0.047	1.163	0.054	1.163	0.047
Q30	$R_{1\rho}$	4.119	0.215	4.069	0.282	4.088	0.339
Q30	R_1	1.115	0.035	1.127	0.063	1.111	0.042
Q32	$R_{1\rho}$	3.803	0.163	3.914	0.179	3.843	0.173
Q32	R_1	1.051	0.030	1.038	0.045	1.082	0.029
Q54	$R_{1\rho}$	2.164	0.102	2.158	0.132	2.164	0.173
Q54	R_1	1.212	0.011	1.220	0.009	1.224	0.011
Q59	$R_{1\rho}$	4.868	0.194	5.059	0.198	4.910	0.169
Q59	R_1	1.152	0.029	1.128	0.031	1.121	0.026
Q61	$R_{1\rho}$	5.211	0.167	5.179	0.212	5.200	0.299
Q61	R_1	1.141	0.030	1.137	0.036	1.151	0.044

Finally, we experimentally assessed the loss in signal intensity upon increasing ^{15}N CSSF selectivity (*i.e.*, the selective 90° pulse duration). We chose Q20 and Q54, which display very different transverse but similar longitudinal relaxation rates (see Table S9). 2D SNIPER experiments were used to resolve the selected correlations from neighboring ones also at short selective pulse lengths. The peak intensity relative to a 1 ms selective (GOLEM) pulse is shown in Figure S16. The loss in signal intensity corresponds to an apparent exponential decay rate of 2.83 s^{-1} for Q54 and 3.87 s^{-1} for Q20. For Q54, this corresponds to about the average in R_1 and R_2, while for Q20, which has a much faster transverse relaxation rate, the apparent decay rate is closer to R_1 than R_2. Future theoretical work will have to reveal the details of R_1 and R_2 weighting during the selective excitation pulse. This empirical result confirms, however, that the selectivity of the ^{15}N CSSF is mostly weighted by the more favorable ^{15}N R_1 relaxation.

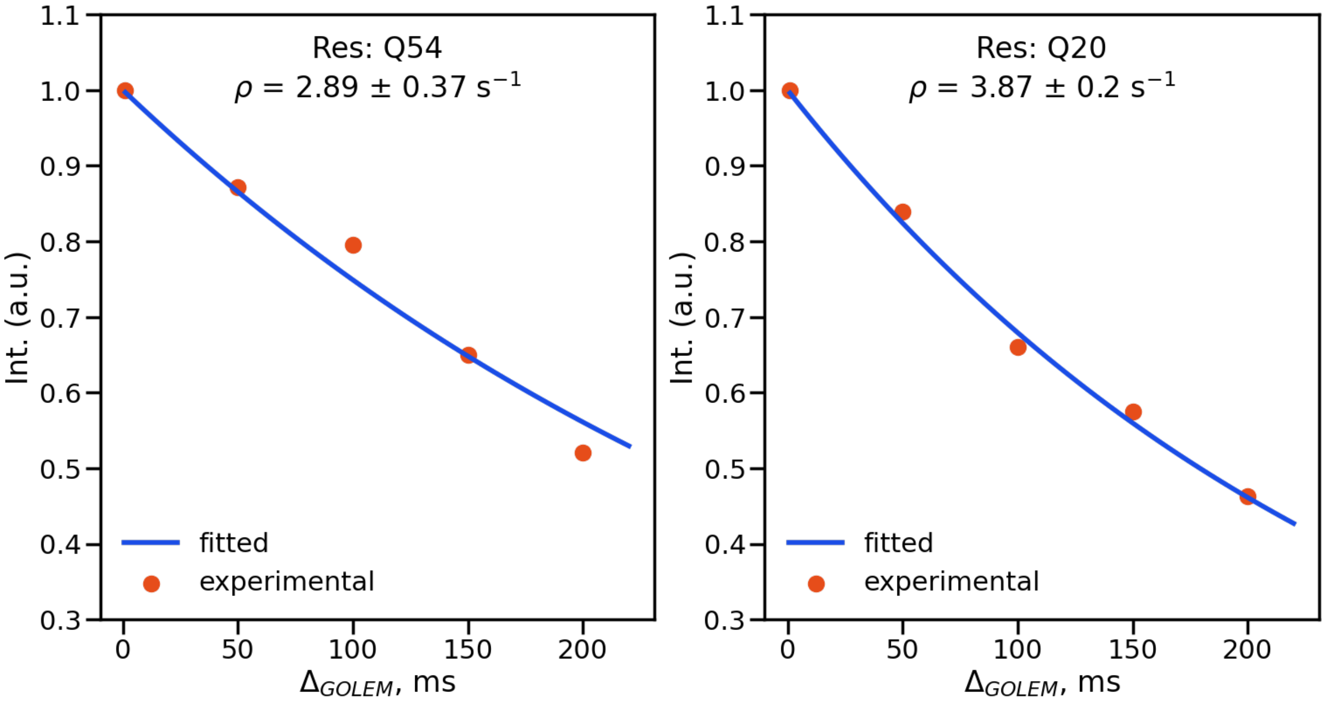

Figure S16. Experimental relative signal intensities (red data points) for Q54 and Q20 upon increasing the ^{15}N CSSF selectivity (GOLEM shaped pulse duration), and an exponential decay function fitted through them (blue line). Data is obtained using a 2D SNIPER R_1 experiment with zero relaxation-encoding delay, NS = 4, SW = 14 ppm, TD1 = 512, Lorentz-to-Gauss apodization in F_1 (LB = -5 Hz, GB = 0.6).

4. Experimental results on Huntingtin exon-1

4.1. Overview of SNIPER experiments

All 1D SNIPER experiments and their variable parameters and total experimental times (including the full series of variable delays for relaxation encoding) are listed in Table S8. Relaxation encoding delays were chosen as described in the experimental section. All SNIPER R_1 and $R_{1\rho}$ experiments used a $^1H \rightarrow ^{15}N$ rSFPT and a $^{15}N \rightarrow ^1H$ HaHa, while the SNIPER nOe experiments used a $^{15}N \rightarrow ^1H$ rSFPT. All experiments used a ^1H CSSF as indicated in the Table, though it was not critical for the overall ^1H selectivity.

There are two cases where a pair of ^1H-^{15}N correlations showed almost identical ^{15}N chemical shifts and the difference in ^1H chemical shift was too small for the selective polarization transfer elements to distinguish them: Q19 and Q22, and Q45 and Q52. In each pair, both residues were therefore selected together in one set of SNIPER experiments. (This did not have a visible impact on their intensity decay profile). Also, the heteronuclear nOes for residues Q18 and Q23 were recorded in the same experiment.

The Q48 and Q59 relaxation data was recorded on a different sample from the rest of the data. ^{15}N $R_{1\rho}$ relaxation on Q61 was measured on each sample, yielding values that were different by merely 4.3%, confirming reproducibility was independent on sample preparation.

In the case of Q59, rather than using a more selective ^{15}N pulse in the CSSF, it was applied 7 Hz off-resonance to avoid excitation of Q48.

The total experimental time for all relaxation data on all residues taken together was 11.46 days, amounting to an average total experimental time per residue of 16.2 hours.

Table S8. 1D SNIPER datasets recorded on huntingtin exon-1.

Residue	Type	^1H CSSF pulse		^{15}N CSSF			N° of transients	Total Exp. time (hours)	Signal-to-noise Ratio*
		Pulse Shape	Sel. Pulse Length (ms)	Pulse Shape	Sel. Pulse Length (ms)	ζ-delay (ms)			
Q18	$R_{1\rho}$	HalfGauss	20	GOLEM	150	0	1760	10.47	104.3
Q18	R_1	HalfGauss	20	GOLEM	150	0	1152	7.48	116.8
Q18&Q23	nOe	Sinc	1	GOLEM	200	0	2816	11.94	34.5 & 34.5
Q19&Q22	$R_{1\rho}$	Sinc	1	GOLEM	100	0	1024	6	74.9 & 74.7
Q19&Q22	R_1	Sinc	1	GOLEM	100	0	800	5.1	83.5 & 83.4
Q19&Q22	nOe	Sinc	1	GOLEM	100	0	2048	8.64	51.2 & 51.1
Q20	$R_{1\rho}$	HalfGauss	10	GOLEM	150	0	1600	9.48	103.3
Q20	R_1	HalfGauss	10	GOLEM	150	0	1024	6.63	101.9
Q20	nOe	Sinc	1	GOLEM	150	0	1536	6.58	31.3
Q21	$R_{1\rho}$	HalfGauss	20	GOLEM	125	0	1600	9.37	102.3
Q21	R_1	HalfGauss	20	GOLEM	125	0	1024	6.50	73.5
Q21	nOe	HalfGauss	1	GOLEM	125	0	2048	8.67	41.4
Q23	$R_{1\rho}$	HalfGauss	10	GOLEM	150	0	1280	7.65	74.7
Q23	R_1	HalfGauss	10	GOLEM	150	0	800	5.12	83.4

Q25	$R_{1\rho}$	Sinc	1	GOLEM	150	0	1024	6.10	97.5
Q25	R_1	Sinc	1	GOLEM	150	0	640	4.12	105.0
Q25	nOe	Sinc	1	GOLEM	150	0	2048	8.70	46.0
Q26	$R_{1\rho}$	Sinc	1	GOLEM	150	0	1024	6.23	86.1
Q26	R_1	Sinc	1	GOLEM	150	0	640	4.08	106.2
Q26	nOe	Sinc	1	GOLEM	150	0	1536	6.60	40.8
Q28	$R_{1\rho}$	Sinc	1	GOLEM	200	0	1920	11.97	90.5
Q28	R_1	Sinc	1	GOLEM	200	0	1280	8.48	108.0
Q28	nOe	Sinc	1	GOLEM	200	0	3072	13.00	44.0
Q30	$R_{1\rho}$	HalfGauss	1	HalfGauss	80	0	800	4.83	83.1
Q30	R_1	HalfGauss	1	HalfGauss	80	0	512	3.22	115.0
Q30	nOe	Sinc	1	HalfGauss	80	0	1536	6.52	40.4
Q32	$R_{1\rho}$	HalfGauss	1	HalfGauss	50	0	800	4.85	91.1
Q32	R_1	HalfGauss	1	HalfGauss	50	0	512	3.22	111.7
Q32	nOe	Sinc	1	HalfGauss	50	0	1536	6.50	39.5
Q45&Q52	$R_{1\rho}$	Sinc	1	HalfGauss	40	0	768	4.48	93.8 & 103.6
Q45&Q52	R_1	Sinc	1	HalfGauss	40	0	512	3.2	112.5 & 124.4
Q45&Q52	nOe	Sinc	1	HalfGauss	40	0	1536	6.48	41.8 & 46.2
Q48	$R_{1\rho}$	Sinc	1	Sinc	35	5	1024	5.85	119.0
Q48	R_1	Sinc	1	Sinc	35	5	512	3.23	113.3
Q48	nOe	Sinc	1	Sinc	35	5	1024	4.30	29.9
Q54	$R_{1\rho}$	HalfGauss	40	GOLEM	100	0	768	5.20	101.1
Q54	R_1	HalfGauss	40	GOLEM	100	0	512	3.32	176.6
Q54	nOe	Sinc	1	GOLEM	100	0	1024	4.45	46.3
Q59	$R_{1\rho}$	Sinc	22	Sinc	22	5	512	4.18	92.6
Q59	R_1	Sinc	22	Sinc	22	5	512	4.38	88.2
Q59	nOe	Sinc	22	Sinc	22	5	1024	6.03	16.3
Q61	$R_{1\rho}$	HalfGauss	10	HalfGauss	10	0	768	4.42	151.5
Q61	R_1	HalfGauss	10	HalfGauss	10	0	512	3.23	149.9
Q61	nOe	Sinc	1	HalfGauss	10	0	1024	4.38	35.9

* SNRs are taken from the 1D SNIPER R_1 and $R_{1\rho}$ experiments with zero relaxation encoding delay or from the SNIPER nOe with recovery delay.

4.2. 1D SNIPER spectra

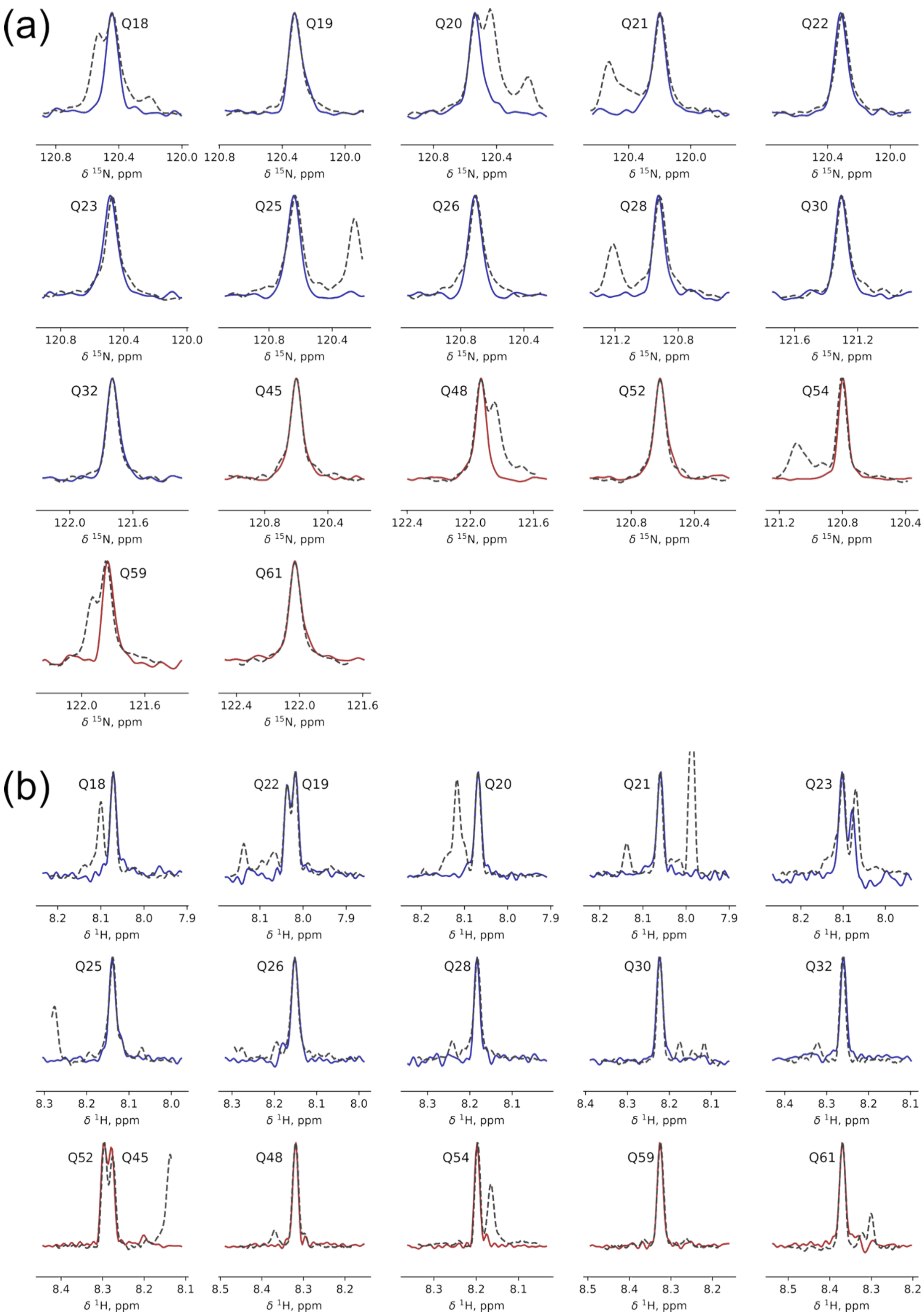

Figure S17. (a) Comparison of F_1-slices taken from all SNIPER 2D spectra (full line) and the standard 2D HSQC (dotted line) shown in Figure 1 of the main manuscript. (b) Comparison of all measured 1D SNIPER ^{1}H spectra (full line) and F_2-slices from the standard 2D HSQC (dotted line) shown in Figure 1 of the main manuscript. (Q23 shows a small change in ^{1}H chemical shift between the 2D HSQC and 1D SNIPER, attributed to sample ripening between both measurements.)

4.3. Relaxation data

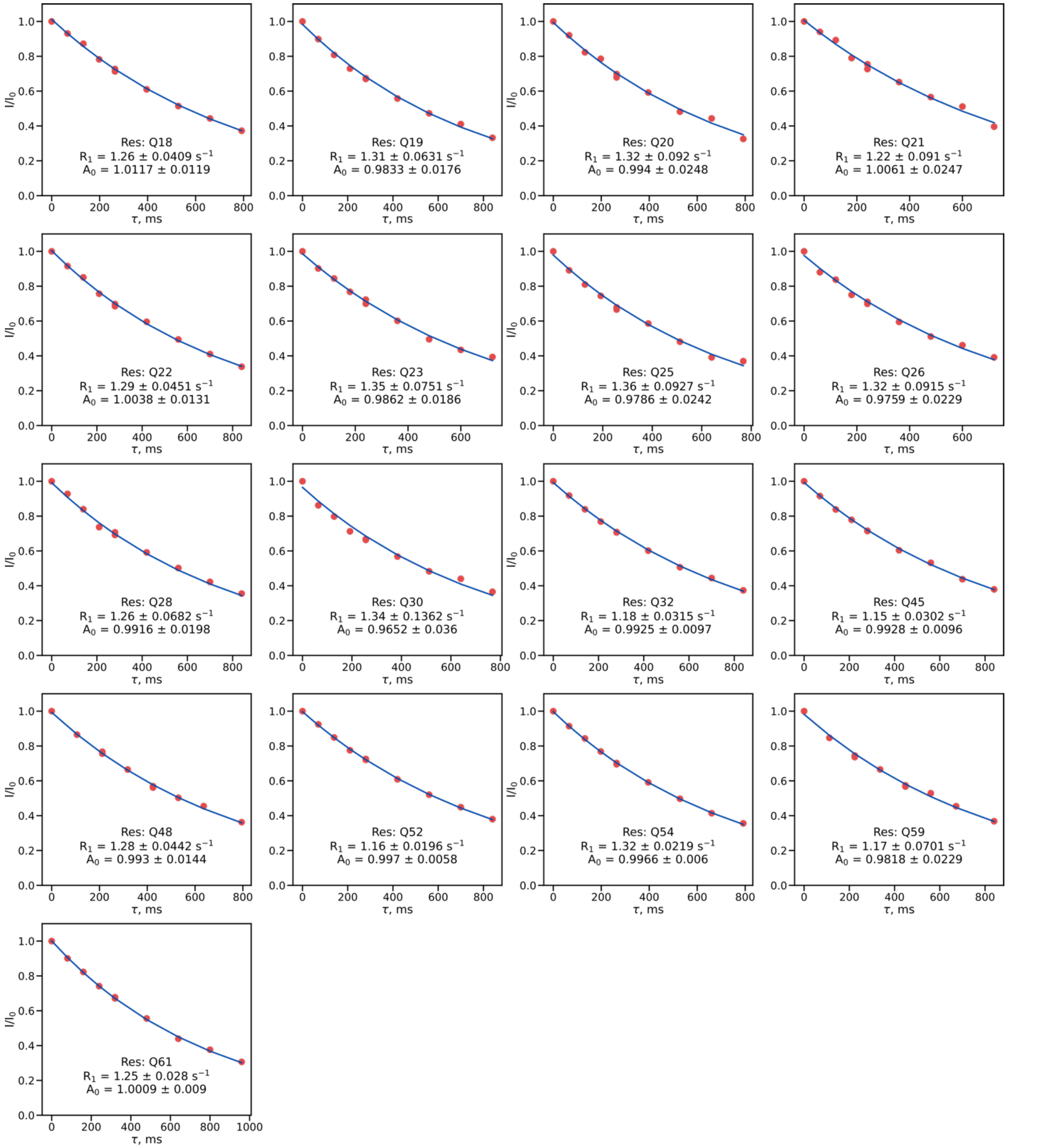

Figure S18. Experimental (red data points) and fitted (blue line) intensity attenuations from 1D SNIPER R_1 data sets acquired on huntingtin exon 1.

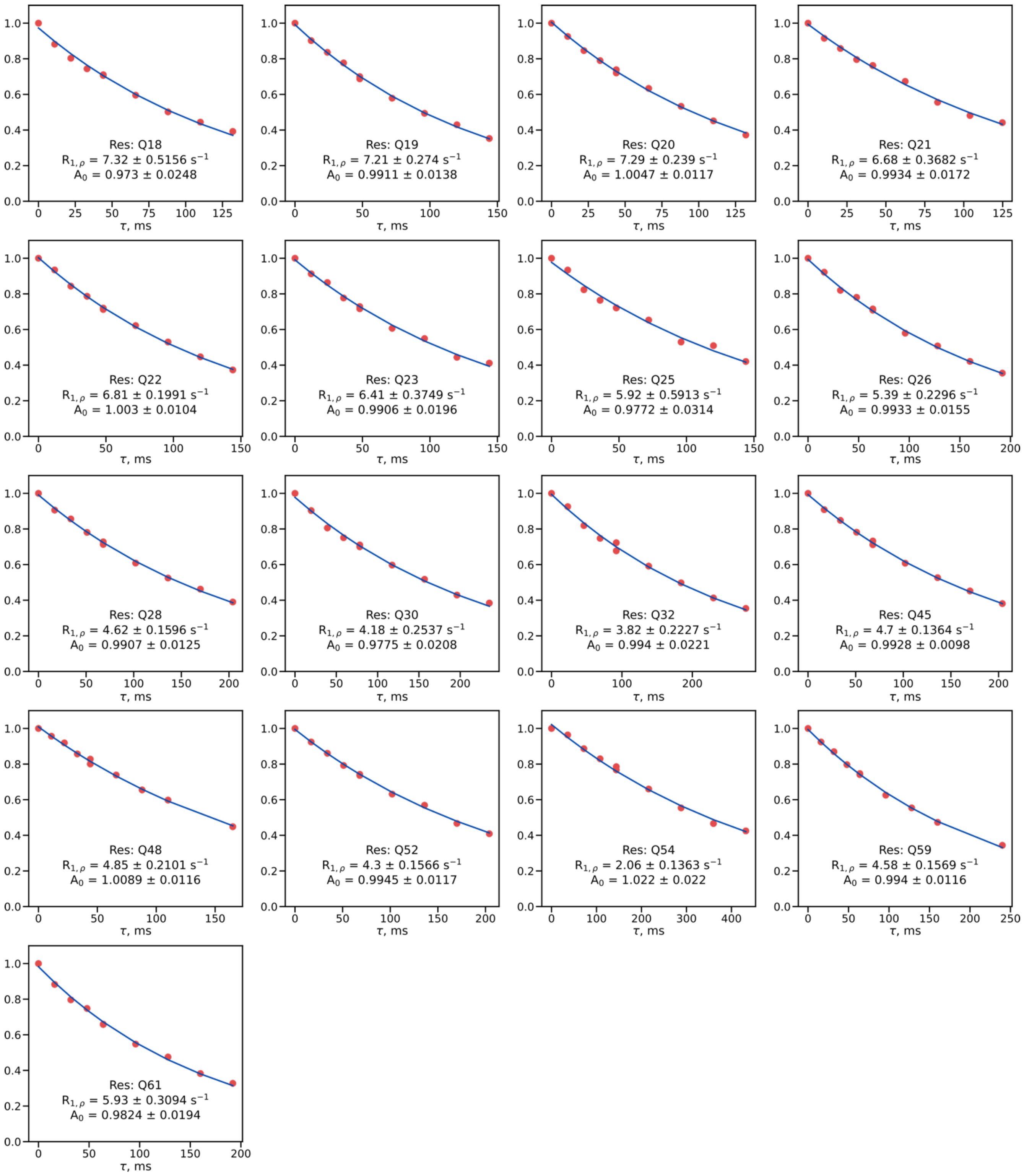

Figure S19. Experimental (red data points) and fitted (blue line) intensity attenuations from 1D SNIPER on-resonance (constant-time) $R_{1\rho}$ data sets acquired on huntingtin exon 1.

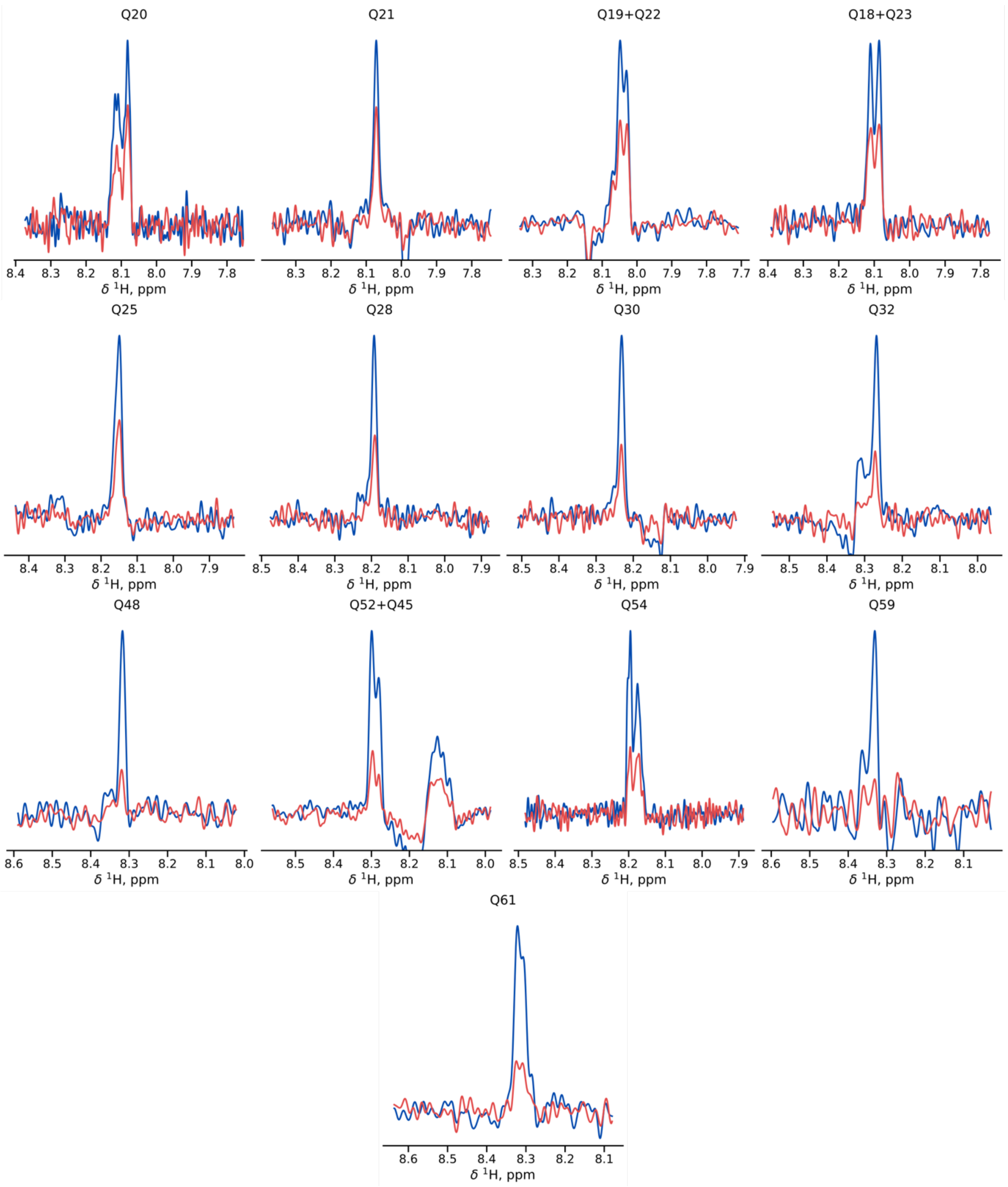

Figure S20. 1D SNIPER {^{1}H}-^{15}N nOe data acquired on huntingtin exon 1. Red spectra are with ^{1}H saturation, blue spectra without saturation but a recovery delay instead. In some of the cases, negative or positive excitation side bands are visible in ^{1}H dimension. This is in contrast to the R_1 and $R_{1\rho}$ datasets and follows from not benefitting only from the rSFPT element rather than both rSFPT and HaHa elements (see section 2.1)

Table S9. Fitted ^{15}N R_1, ^{15}N $R_{1\rho}$ and $\{^1H\}$-^{15}N nOe values and 95% error of fitting estimates for huntingtin exon-1.

Residue	R_1 (s^{-1})	ΔR_1 (s^{-1})	$R_{1\rho}$ (s^{-1})	$\Delta R_{1\rho}$ (s^{-1})	R_2 (s^{-1})	ΔR_2 (s^{-1})	het-nOe	Δhet-nOe
Q18	1.261	0.041	7.323	0.516	8.584	0.517	0.55	0.03
Q19	1.310	0.063	7.215	0.274	8.525	0.281	0.64	0.02
Q20	1.320	0.092	7.292	0.239	8.612	0.256	0.64	0.03
Q21	1.218	0.091	6.676	0.368	7.894	0.379	0.62	0.02
Q22	1.290	0.045	6.810	0.199	8.100	0.204	0.56	0.02
Q23	1.351	0.075	6.412	0.375	7.764	0.382	0.58	0.03
Q25	1.363	0.093	5.921	0.591	7.284	0.598	0.55	0.02
Q26	1.318	0.092	5.385	0.230	6.703	0.247	0.53	0.02
Q28	1.261	0.068	4.622	0.160	5.883	0.174	0.45	0.03
Q30	1.339	0.136	4.176	0.254	5.515	0.288	0.38	0.02
Q32	1.178	0.032	3.824	0.223	5.002	0.225	0.31	0.02
Q45	1.150	0.030	4.695	0.136	5.845	0.140	0.29	0.02
Q48	1.280	0.044	4.847	0.210	6.127	0.215	0.23	0.03
Q52	1.157	0.020	4.300	0.157	5.456	0.158	0.32	0.02
Q54	1.322	0.022	2.055	0.136	3.377	0.138	0.34	0.01
Q59	1.172	0.070	4.581	0.157	5.753	0.172	0.19	0.03
Q61	1.250	0.028	5.931	0.309	7.182	0.311	0.24	0.02

R_2 values were calculated as $R_2 = R_1 + R_{1\rho}$ values were obtained. The R_1 and $R_{1\rho}$ 95% uncertainties were obtained using Monte Carlo error analysis.[30] The reported nOe uncertainties were calculated from the standard deviation of the spectral noise found in an empty region of the spectrum.

5. Experimental results on the SH3 domain of SH3GL3

5.1. SH3GL3-SH3 2D spectra and assignment

An overlay of two standard 2D ^{1}H-^{15}N *zz*-exchange spectra[1] with 0 and 750 ms mixing times is shown in Figure S21. For several residues (G18, G64 and G48) diagonal and cross-peaks overlap with each other or with other cross-peaks in either the ^{15}N or ^{1}H dimensions.

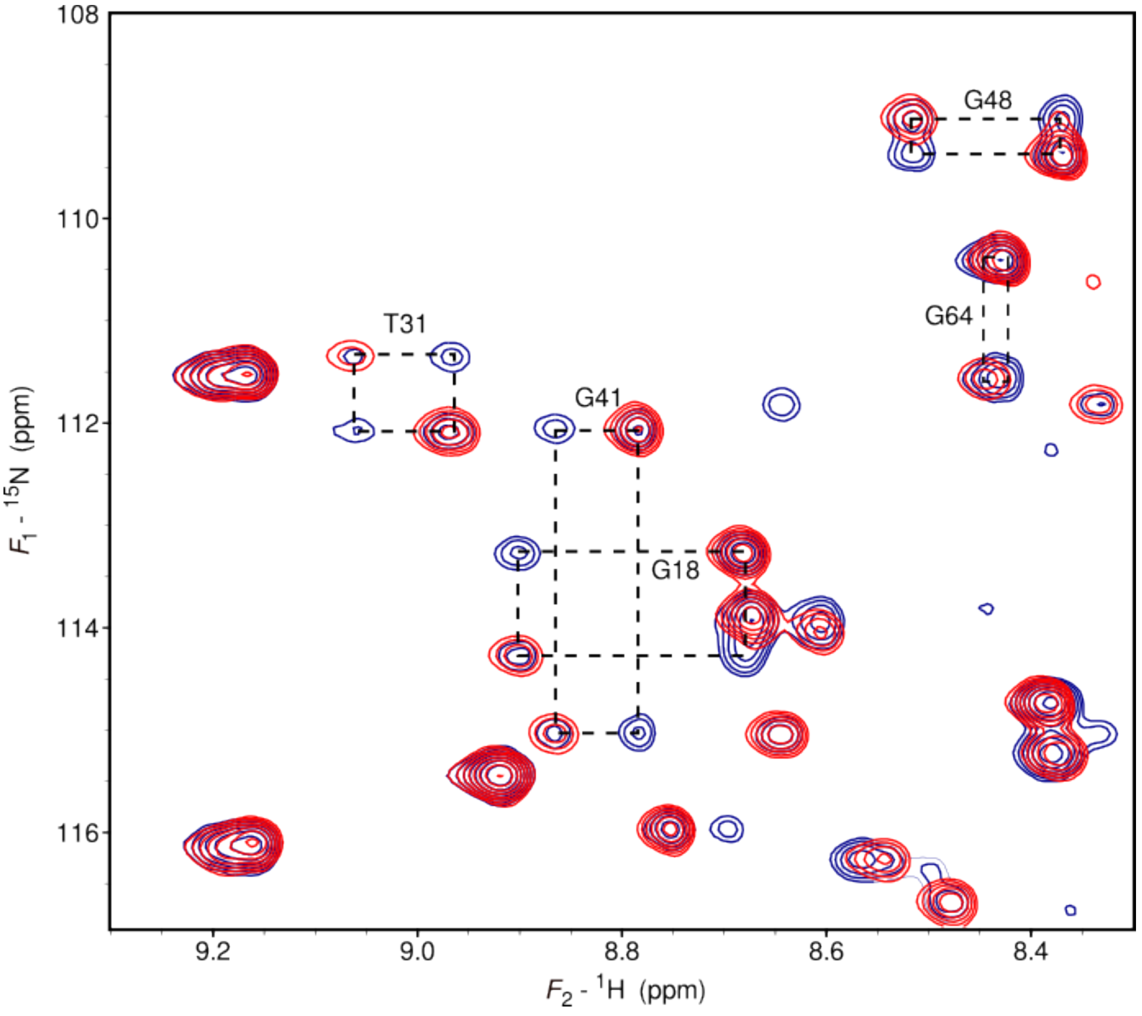

Figure S21. Part of ^{1}H-^{15}N 2D *zz*-exchange spectra of SH3GL3 SH3 domain. Spectra with exchange mixing times of 0 ms (red) and 750 ms (blue) are overlayed. Some examples of residues showing slow exchange between two forms are indicated. Dotted rectangles connect the diagonal peaks and exchange cross-peaks for a selected number of residues.

5.2. Overview of *zz*-exchange experiments

A standard 2D zz-exchange experiment was measured.[1] On a selected number of residues, selective *zz*-exchange experiments were measured: 1D SNIPER *zz*-exchange and the 2D F_1F_2-selective approach.[2, 25] An overview of these selective experiments is shown in Table S10. All SNIPER experiments used a 5 ms ζ-delay in the ^{15}N CSSF.

For all *zz*-exchange experiments, 12 mixing times in the range of 0 to 750 ms were chosen. The total experimental time using the standard 2D zz-exchange experiment, was 125 hours, while for the selective experiments the total experimental time for each residue is reported in Table S10.

Table S10. 1D SNIPER and F_1F_2-selective *zz*-exchange datasets recorded on SH3GL3-SH3.

Residue	Form	1D SNIPER *zz*-exchange				F_1F_2-selective *zz*-exchange	
		^{15}N CSSF selective pulse shape	^{15}N CSSF selective pulse length (ms)	Number of transients	Exp. Time (hours)	Number of transients	Exp. Time (hours)
C5	Major	Sinc	11.11	512	3.43	512	27.44
C5	minor	Sinc	11.11	512	3.43	512	27.44
N16	Major	Sinc	11.11	256	1.72	512	13.72
N16	minor	Sinc	11.11	256	1.72	512	13.72
Q33	Major	Sinc	7.85	512	3.42	512	13.72
Q33	minor	Sinc	7.85	512	3.43	512	13.72
W38	Major	Sinc	7.85	512	3.43	512	13.72
W38	minor	Sinc	7.85	512	3.43	512	13.72
E40	Major	Sinc	7.85	512	3.43	512	13.72
E40	minor	Sinc	7.85	512	3.42	512	13.72
G48	Major	Sinc	7.85	512	3.43	512	13.72
G48	minor	Sinc	7.85	512	3.43	512	13.72
S66	Major	Sinc	7.85	512	3.43	408	10.93
S66	minor	Sinc	7.85	512	3.43	408	10.93

5.3. Exchange data fitting procedure

Diagonal and cross-peak intensities depend on the mixing time τ according to the following equations:[1]

$$I_{EE}(\tau) = I_{EE}(0)\left(-(\lambda_2 - a_{11})e^{-\lambda_1\tau} + (\lambda_1 - a_{11})e^{-\lambda_2\tau}\right)/(\lambda_1 - \lambda_2)$$

$$I_{GG}(\tau) = I_{GG}(0)\left(-(\lambda_2 - a_{22})e^{-\lambda_1\tau} + (\lambda_1 - a_{22})e^{-\lambda_2\tau}\right)/(\lambda_1 - \lambda_2)$$

$$I_{GE}(\tau) = \kappa_{GE} I_{GG}(0)\left(a_{12}e^{-\lambda_1\tau} + a_{12}e^{-\lambda_2\tau}\right)/(\lambda_1 - \lambda_2)$$

$$I_{EG}(\tau) = \kappa_{EG} I_{EE}(0)\left(a_{21}e^{-\lambda_1\tau} + a_{21}e^{-\lambda_2\tau}\right)/(\lambda_1 - \lambda_2)$$

$$\lambda_{1,2} = \frac{1}{2}\left((a_{11} + a_{22}) \pm \left((a_{11} - a_{22})^2 + 4k_{GE}k_{EG}\right)^{0.5}\right)$$

$$a_{11} = R_E + k_{EG};\ a_{22} = R_G + k_{GE};\ a_{12} = -k_{GE};\ a_{21} = -k_{EG}$$

with I_{GG}, I_{EE} the intensities of ground and excited state diagonal peaks, I_{GE} and I_{EG} the intensities of ground and excited state cross-peaks, k_{GE} the ground to excited exchange rate, k_{EG} the excited to ground exchange rate, and R_G and R_E the ^{15}N longitudinal auto-relaxation rates of ground and excited states, respectively. $I_{GG}(0)$ and $I_{EE}(0)$, are the initial intensities of ground and excited states. κ_{GE} and κ_{EG} are scaling factors that account for differential relaxation weighting of the ground and excited states during the *zz*-exchange pulse sequences before and after the mixing time. The parameters $I_{GG}(0)$, $I_{EE}(0)$, κ_{GE}, κ_{EG}, R_G, R_E and k_{GE} and k_{GE} were optimized during data fitting in the same way for each type of *zz*-exchange experiment. This large number of unknowns implies a large degree of covariance between them, meaning high 95% uncertainty values. The estimated parameter values should therefore be regarded with skepticism. Attempts at restraining the k_{GE} and k_{EG} values based on intensity ratios between Major and minor forms in the HSQC, as suggested before,[1] did not lead to a good model fitting, suggesting the intensities experience significantly different relaxation weighting. Preliminary ^{15}N and $^1H^N$ R_2 measurements indeed confirmed a consistent factor of ca. 1.5 difference, not shown. Contributions from more complex exchange phenomena than simple two-site exchange can also not be excluded. Further experimental work would be needed to further characterize the exchange process and improve on the reliability of data fitting, which was not pursued here. Results from the data fitting are shown for illustrative purposes only, to show that intensity profile curves are well-behaved.

5.4. Comparison of 1D SNIPER, the standard 2D method and F_1F_2-selective *zz*-exchange experiments

For accurate data fitting, it is key to resolve the individual diagonal and cross-peaks. The standard INEPT-based 2D zz-exchange method[1] can do so in principle, but at the cost of high experimental times. On SH3 domain of SH3GL3, it was applied with 255 total t_1 time domain points using a 30 ppm spectral window (at 600.13 MHz), resulting in an F_1 spectral resolution of 3.52 Hz. This was sufficient to resolve the diagonal and cross-peaks for residues C5, S66, E40, G48 and W38 (Figures S22-S29, S32 and S33), but not for N16 and Q33, leading to inaccurate intensity curves as a function of mixing time (Figures S30, S31, S34 and S35).

An alternative method is the F_1F_2-selective experiment.[2] It uses a single HaHa polarization transfer to select one of the diagonal ^{1}H-^{15}N correlations. This results in a far lower ^{15}N selectivity than the SNIPER *zz*-exchange experiment. The F_1F_2-selective experiment compensates for this by using a t_1 incrementation to disperse any nearby ^{15}N responses along a second dimension just as in the standard 2D method, albeit with a narrower spectral window and thus still a gain in minimal experimental time. A key difference to both the standard 2D method and SNIPER is that this t_1 incrementation is implemented *after* the exchange mixing time. This means that the exchange cross-peak will be found at the ^{1}H and ^{15}N chemical shifts of the other form rather than at the ^{15}N chemical shift of the selected diagonal peak. If the other diagonal peak was inadvertently also partly excited by the single HaHa transfer (for instance due to its significant excitation side-bands, as shown in section 2.1), it would thus contribute to the cross-peak intensity, resulting in inaccurate intensity profiles. The F_1F_2-selective method thus provides good results only when the diagonal peaks are well-separated in both the ^{15}N and ^{1}H dimensions, *i.e.*, more than the excitation range of a single HaHa transfer (*e.g.*, for residues S66, W38 or E40, Figures S22-S24, S28 and S29).

The SNIPER *zz*-exchange method avoids all these complications, allowing selection of individual diagonal peaks even when their ^{15}N chemical shifts are very close, and maximizes the reduction in experimental time by permitting 1D measurements. The resulting intensity curves as a function of mixing time are therefore consistently more well-behaved than the standard 2D method and the F_1F_2-selective method in all cases shown below (Figures S22-S35).

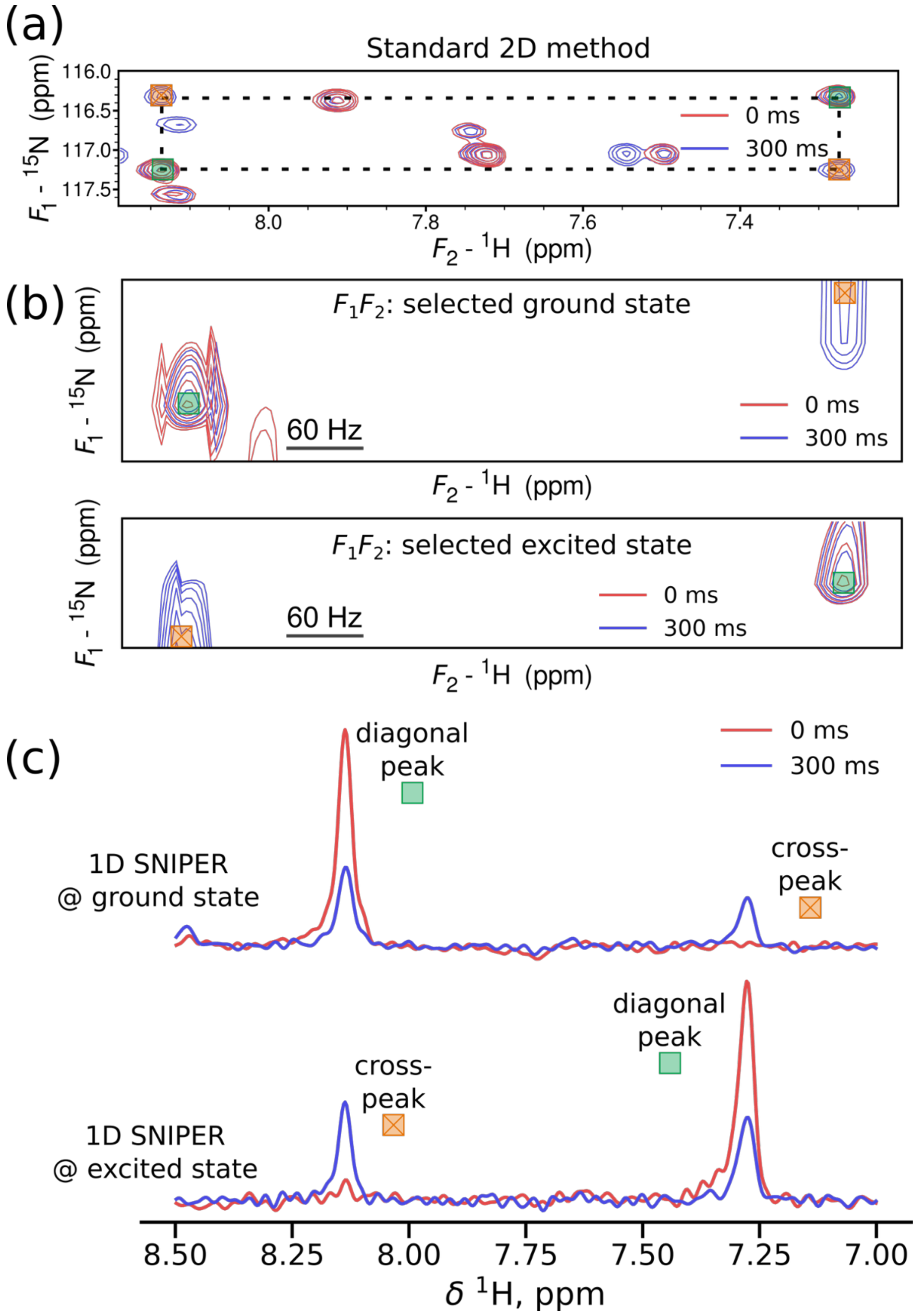

Figure S22. *zz*-exchange experiments focusing on residue S66 of SH3GL3-SH3. (a) Part of the standard *zz*-exchange 2D spectra, overlaying mixing times 0 (red) and 300 ms (blue). (b) Part of the F_1F_2-selective experiment spectra, selecting for the ^{15}N chemical shifts of each form, overlaying mixing times 0 (red) and 300 ms (blue). (c) 1D SNIPER spectra, selecting for the ^{15}N chemical shifts of each form. Red spectra are at 0 ms mixing time, blue spectra at 300 ms mixing time. Throughout, diagonal peaks are marked with green squares, cross-peaks with crossed orange squares.

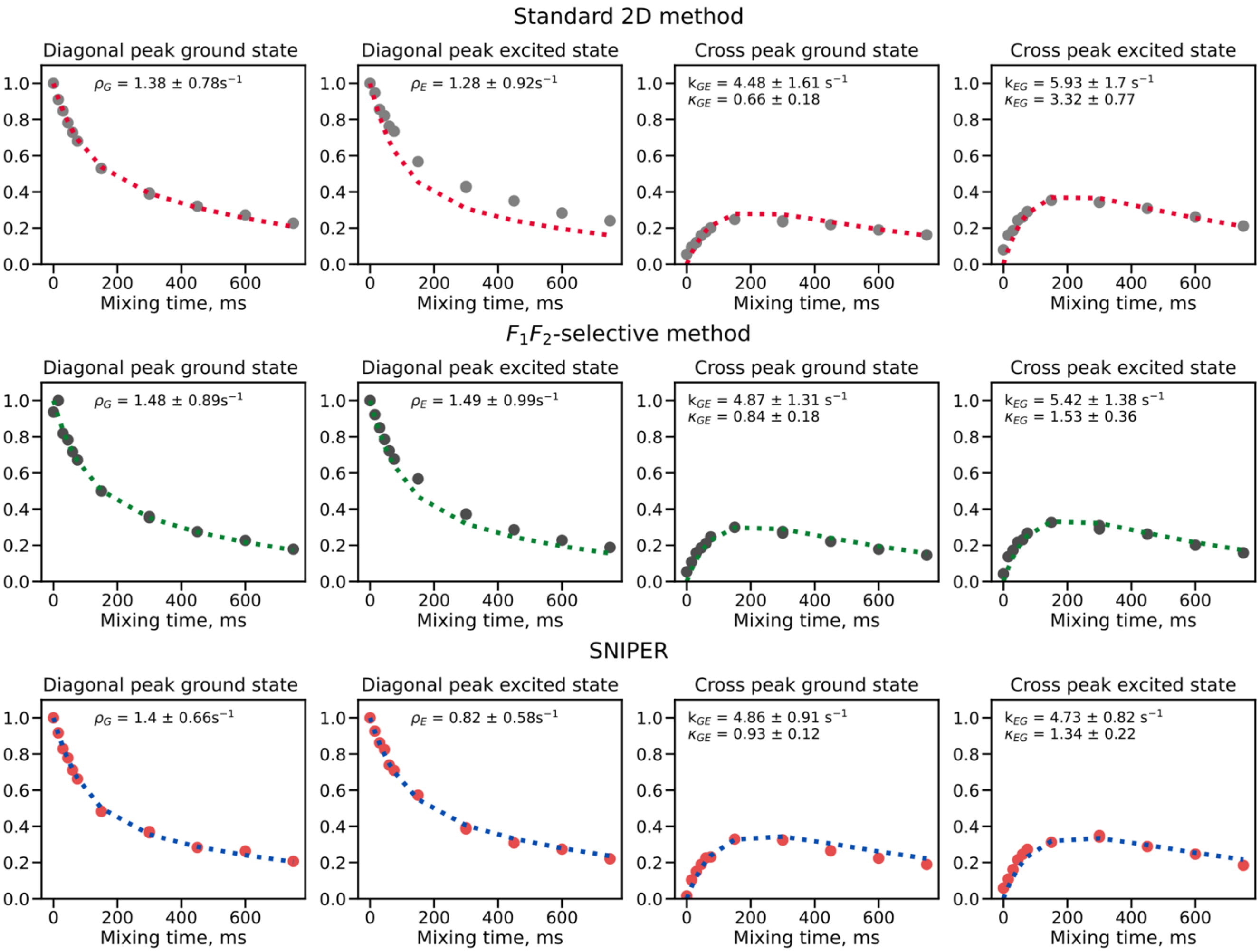

Figure S23. Intensity profiles as a function of mixing time for residue S66, obtained via (a) the standard zz-exchange 2D method, (b) the F_1F_2-selective method, or (c) the 1D SNIPER method. The numbers inside the plot refer to the ^{15}N R_1 values of each form (ρ_G and ρ_E), forward and backward exchange rates (k_{GE} and k_{EG}), as well as pre-factors that account for differential relaxation weighting of the ground and excited states (κ_{GE} and κ_{EG}).

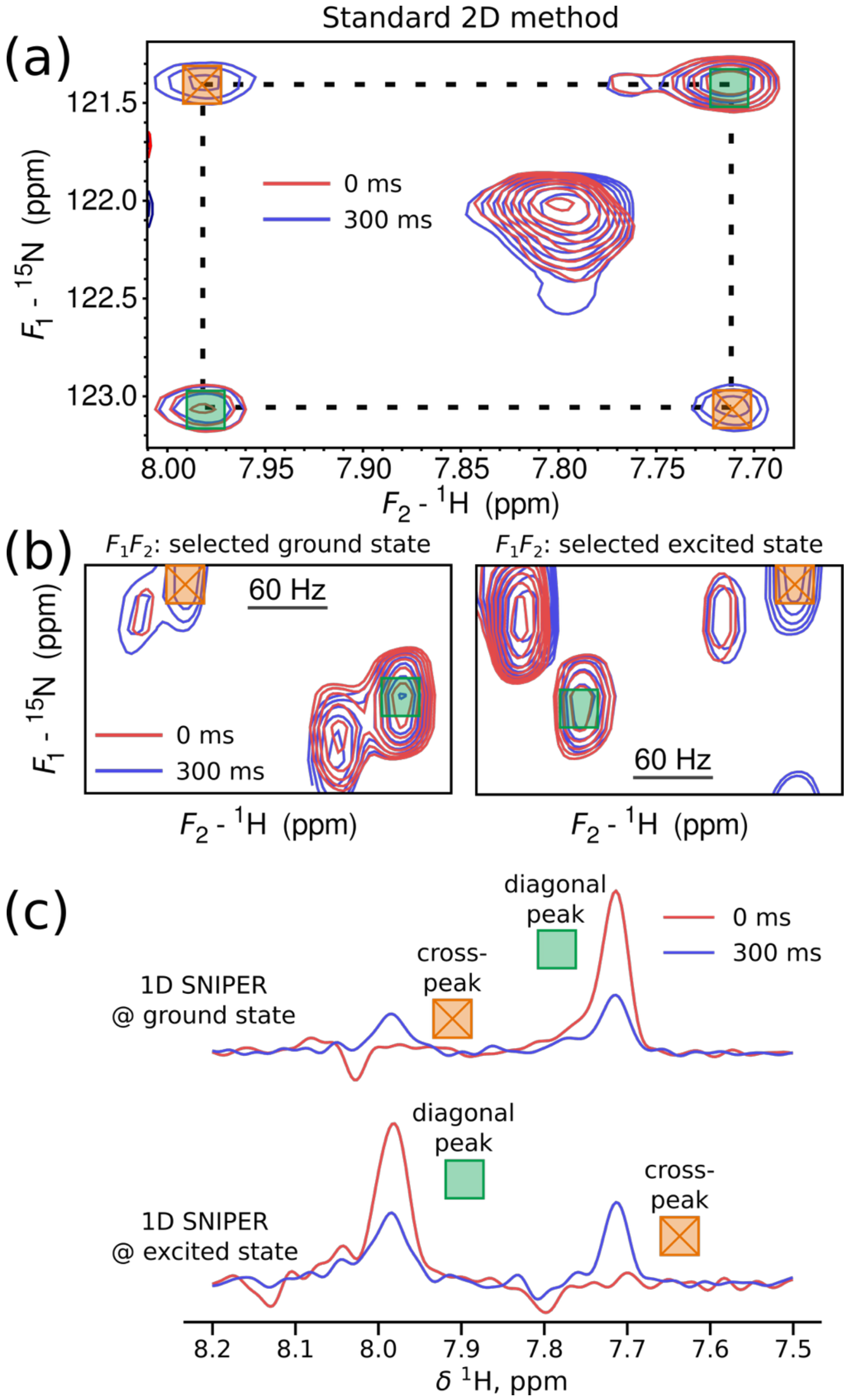

Figure S24. *zz*-exchange experiments focusing on residue W38 of SH3GL3-SH3. (a) Part of the standard *zz*-exchange 2D spectra, overlaying mixing times 0 (red) and 300 ms (blue). (b) Part of the F_1F_2-selective experiment spectra, selecting for the ^{15}N chemical shifts of each form, overlaying mixing times 0 (red) and 300 ms (blue). (c) 1D SNIPER spectra, selecting for the ^{15}N chemical shifts of each form. Red spectra are at 0 ms mixing time, blue spectra at 300 ms mixing time. Throughout, diagonal peaks are marked with green squares, cross-peaks with crossed orange squares.

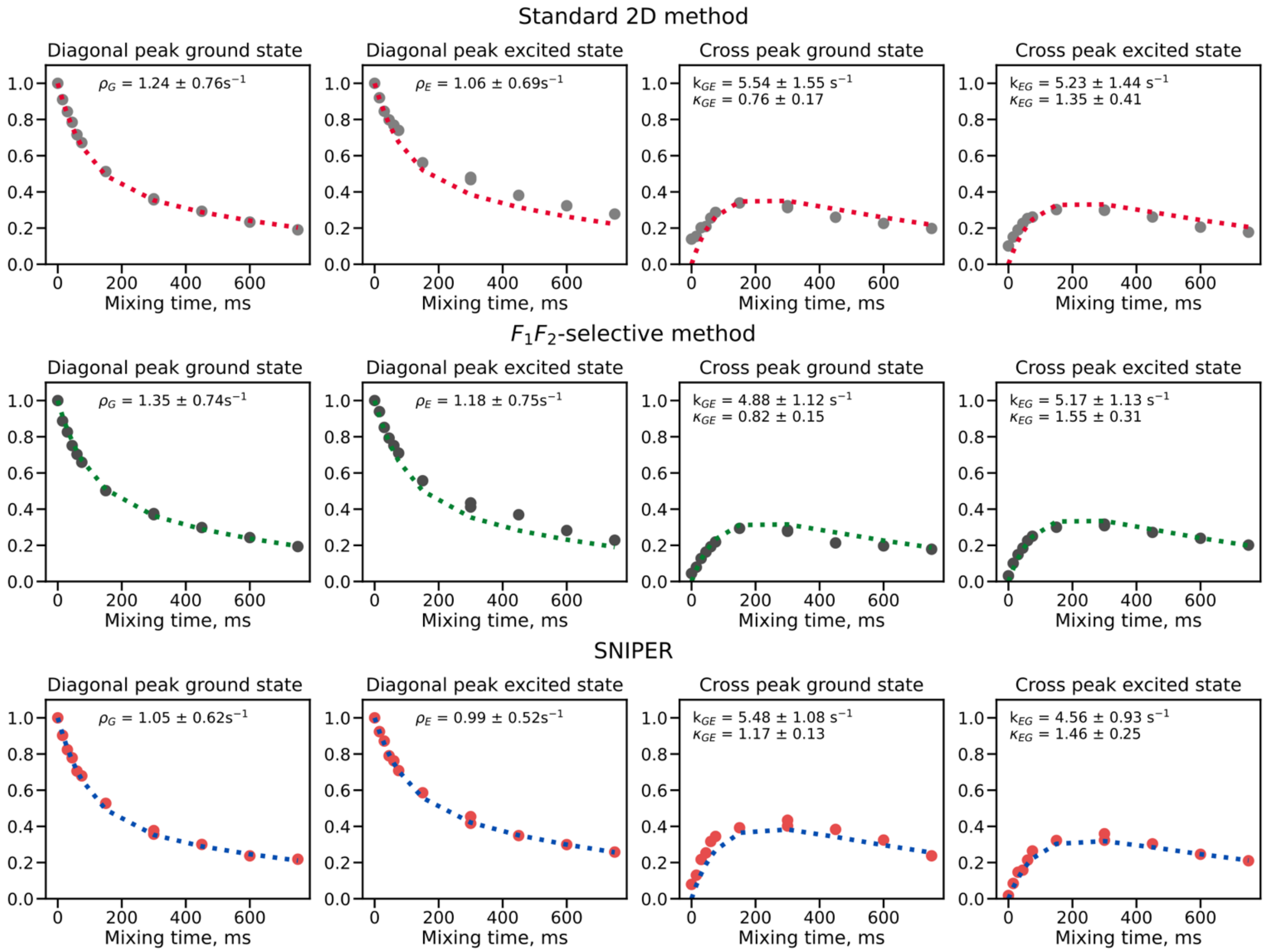

Figure S25. Intensity profiles as a function of mixing time for residue W38, obtained via (a) the standard zz-exchange 2D method, (b) the F_1F_2-selective method, or (c) the 1D SNIPER method. The numbers inside the plot refer to the ^{15}N R_1 values of each form (ρ_G and ρ_E), forward and backward exchange rates (k_{GE} and k_{EG}), as well as pre-factors that account for differential relaxation weighting of the ground and excited states (κ_{GE} and κ_{EG}).

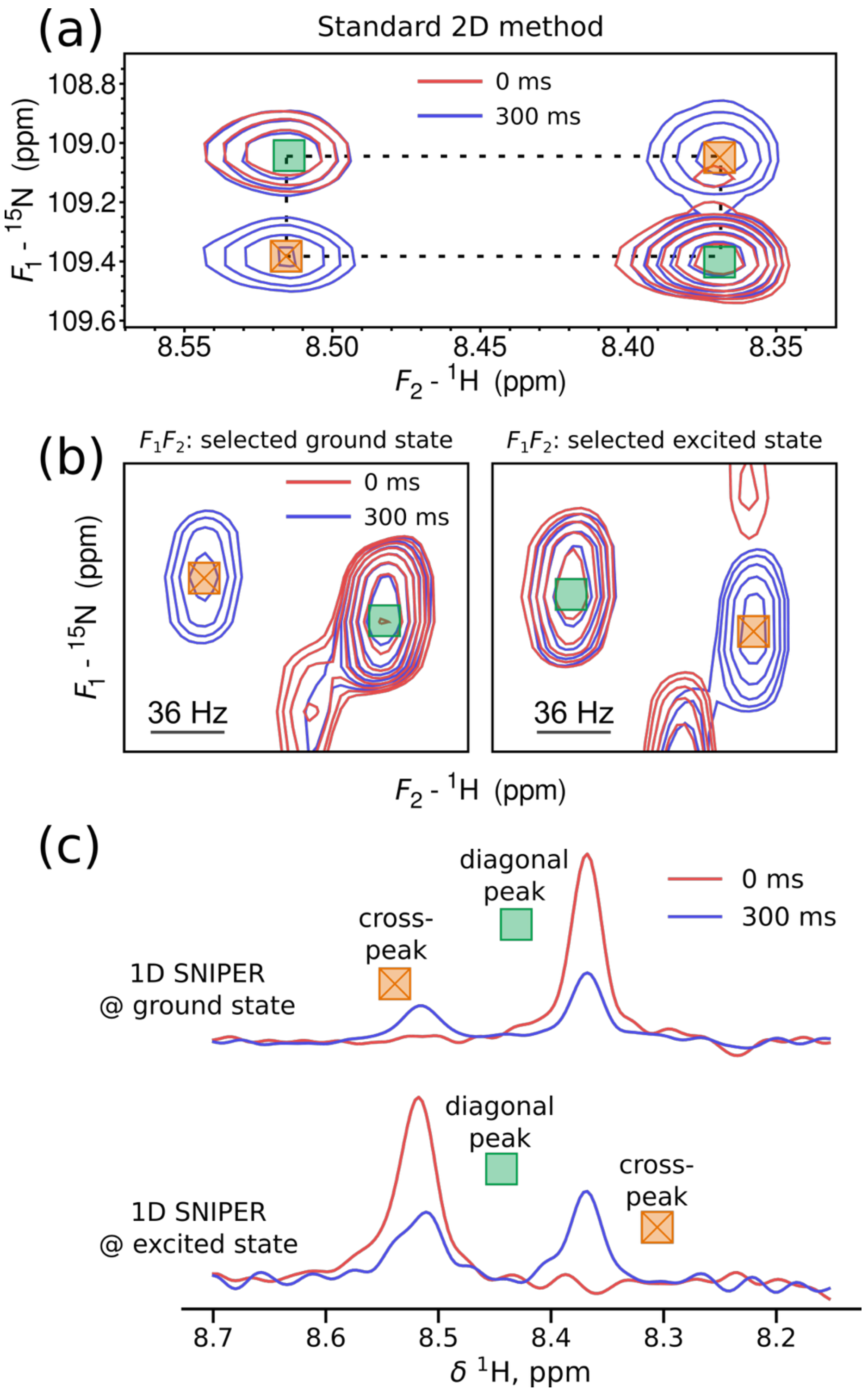

Figure S26. *zz*-exchange experiments focusing on residue G48 of SH3GL3-SH3. (a) Part of the standard *zz*-exchange 2D spectra, overlaying mixing times 0 (red) and 300 ms (blue). (b) Part of the F_1F_2-selective experiment spectra, selecting for the ^{15}N chemical shifts of each form, overlaying mixing times 0 (red) and 300 ms (blue). (c) 1D SNIPER spectra, selecting for the ^{15}N chemical shifts of each form. Red spectra are at 0 ms mixing time, blue spectra at 300 ms mixing time. Throughout, diagonal peaks are marked with green squares, cross-peaks with crossed orange squares.

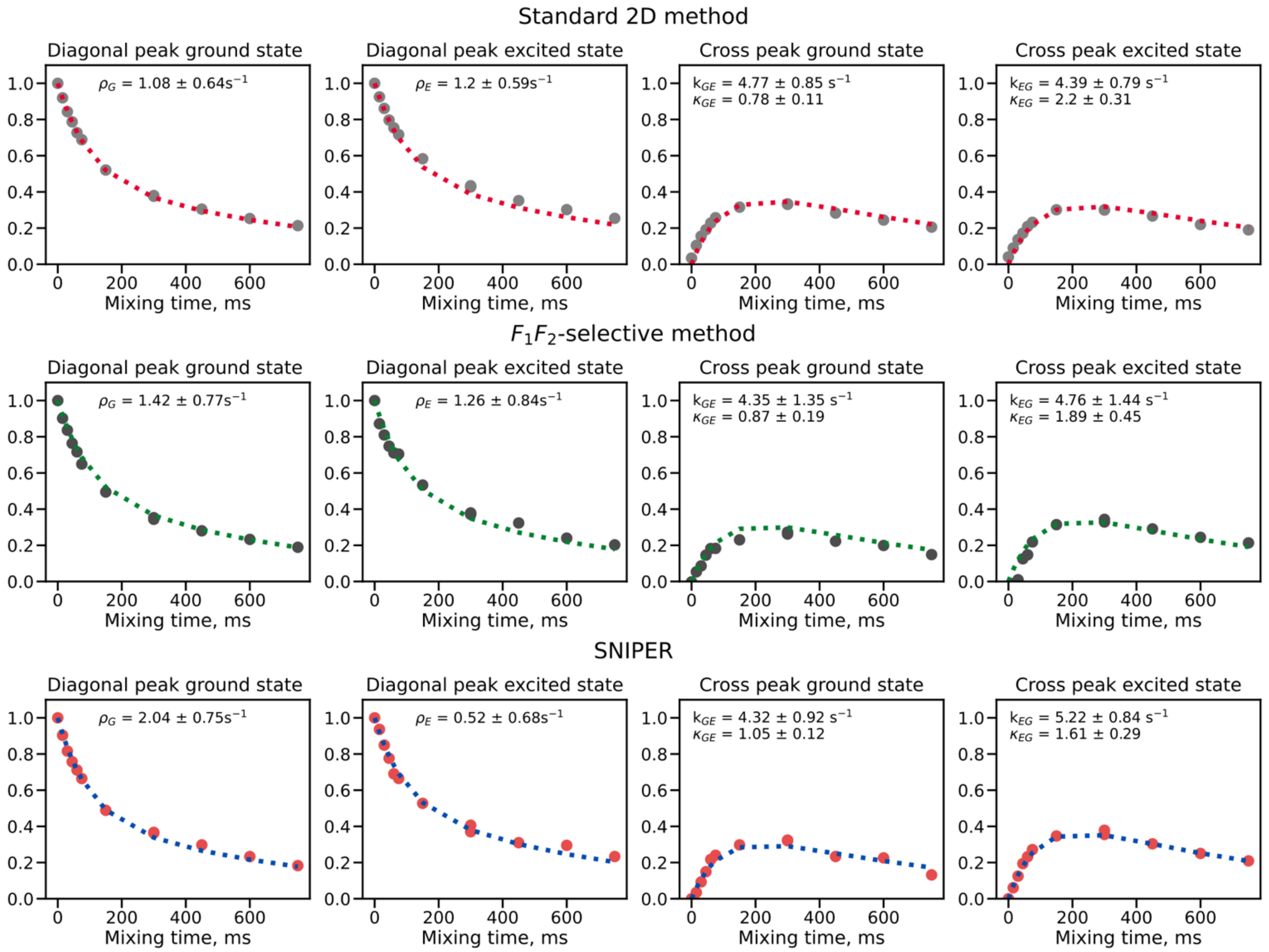

Figure S27. Intensity profiles as a function of mixing time for residue G48, obtained via (a) the standard zz-exchange 2D method, (b) the F_1F_2-selective method, or (c) the 1D SNIPER method. The numbers inside the plot refer to the ^{15}N R_1 values of each form (ρ_G and ρ_E), forward and backward exchange rates (k_{GE} and k_{EG}), as well as pre-factors that account for differential relaxation weighting of the ground and excited states (κ_{GE} and κ_{EG}).

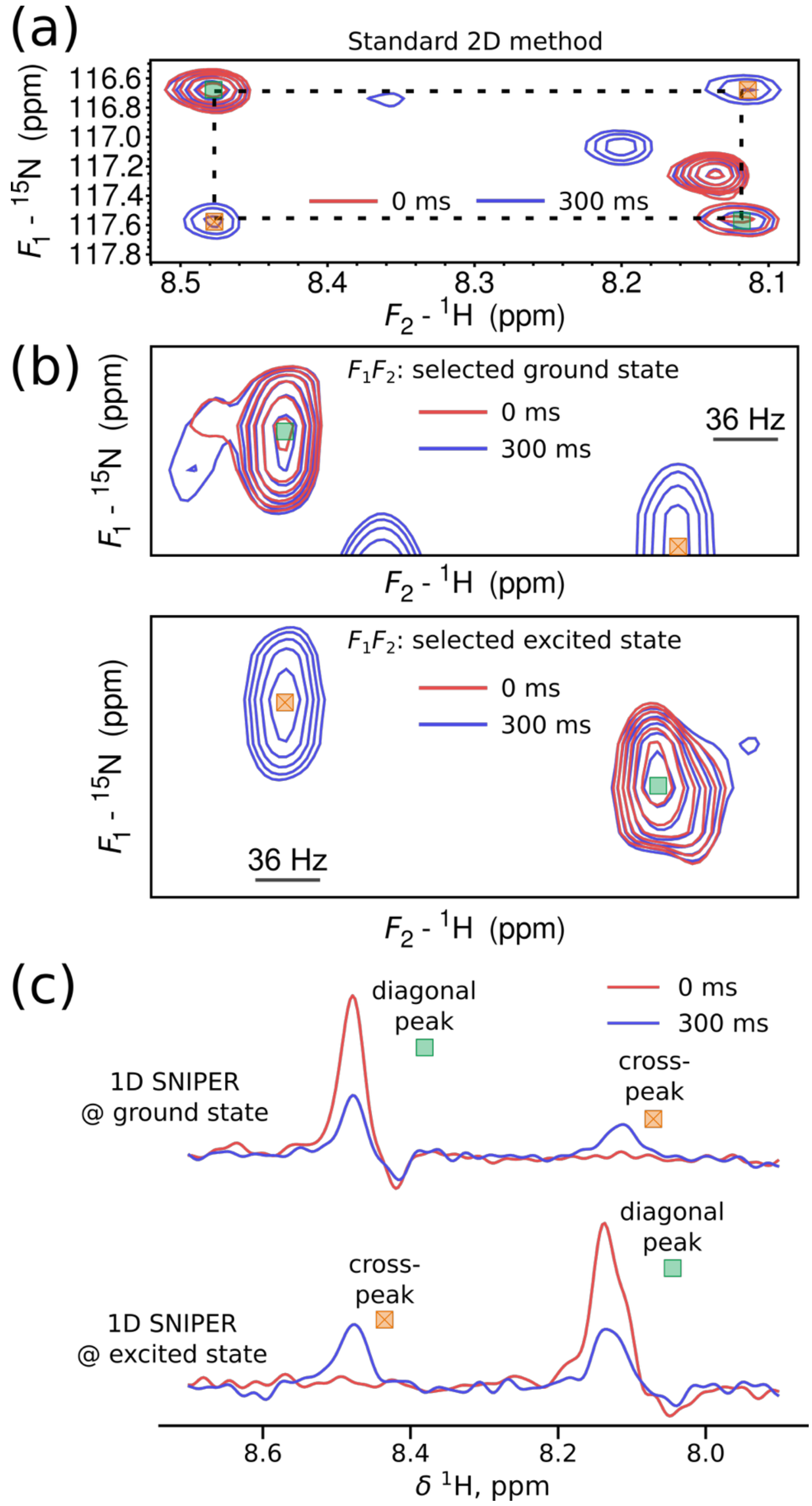

Figure S28. *zz*-exchange experiments focusing on residue E40 of SH3GL3-SH3. (a) Part of the standard *zz*-exchange 2D spectra, overlaying mixing times 0 (red) and 300 ms (blue). (b) Part of the F_1F_2-selective experiment spectra, selecting for the ^{15}N chemical shifts of each form, overlaying mixing times 0 (red) and 300 ms (blue). (c) 1D SNIPER spectra, selecting for the ^{15}N chemical shifts of each form. Red spectra are at 0 ms mixing time, blue spectra at 300 ms mixing time. Throughout, diagonal peaks are marked with green squares, cross-peaks with crossed orange squares.

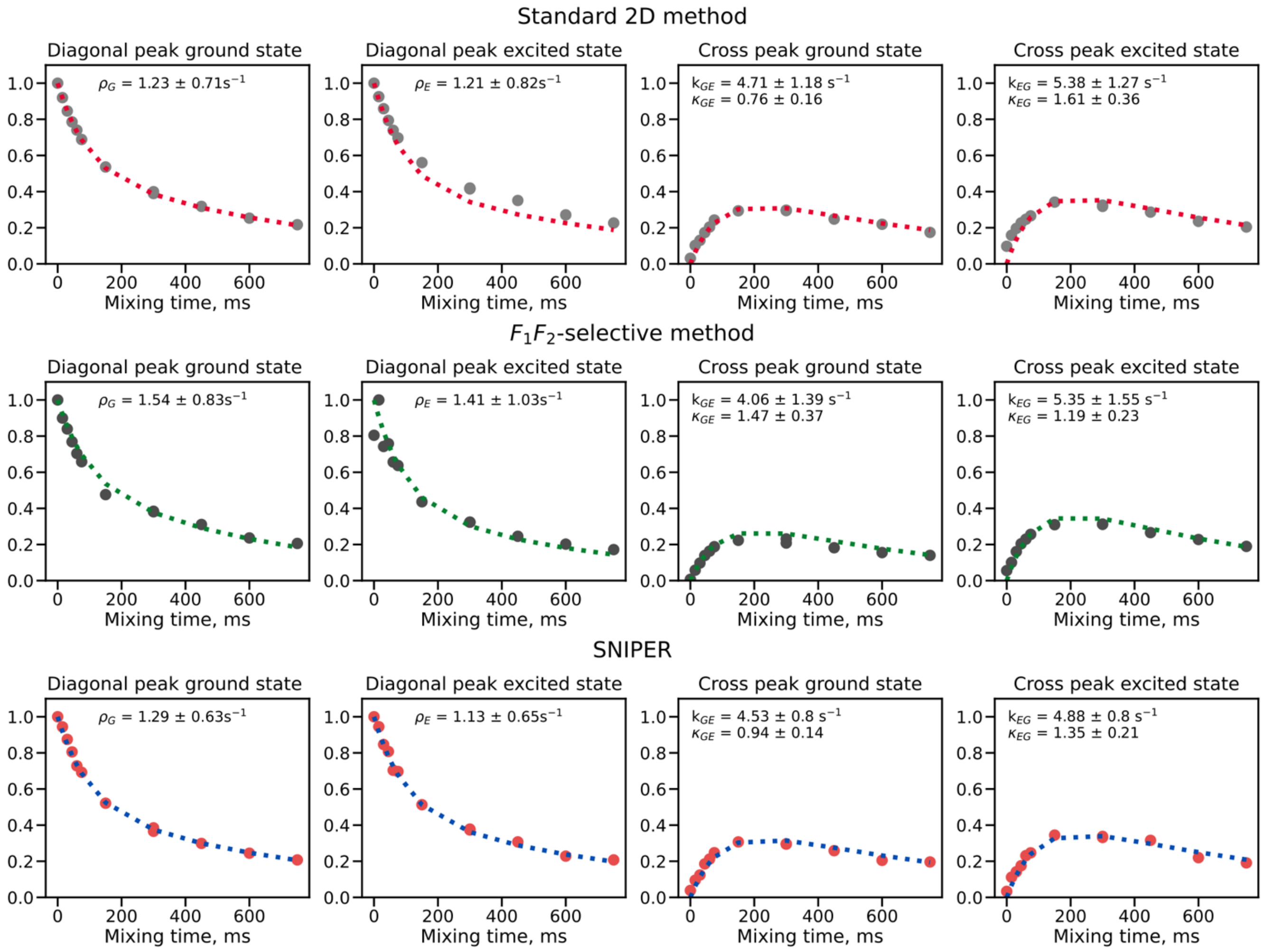

Figure S29. Intensity profiles as a function of mixing time for residue E40, obtained via (a) the standard zz-exchange 2D method, (b) the F_1F_2-selective method, or (c) the 1D SNIPER method. The numbers inside the plot refer to the ^{15}N R_1 values of each form (ρ_G and ρ_E), forward and backward exchange rates (k_{GE} and k_{EG}), as well as pre-factors that account for differential relaxation weighting of the ground and excited states (κ_{GE} and κ_{EG}).

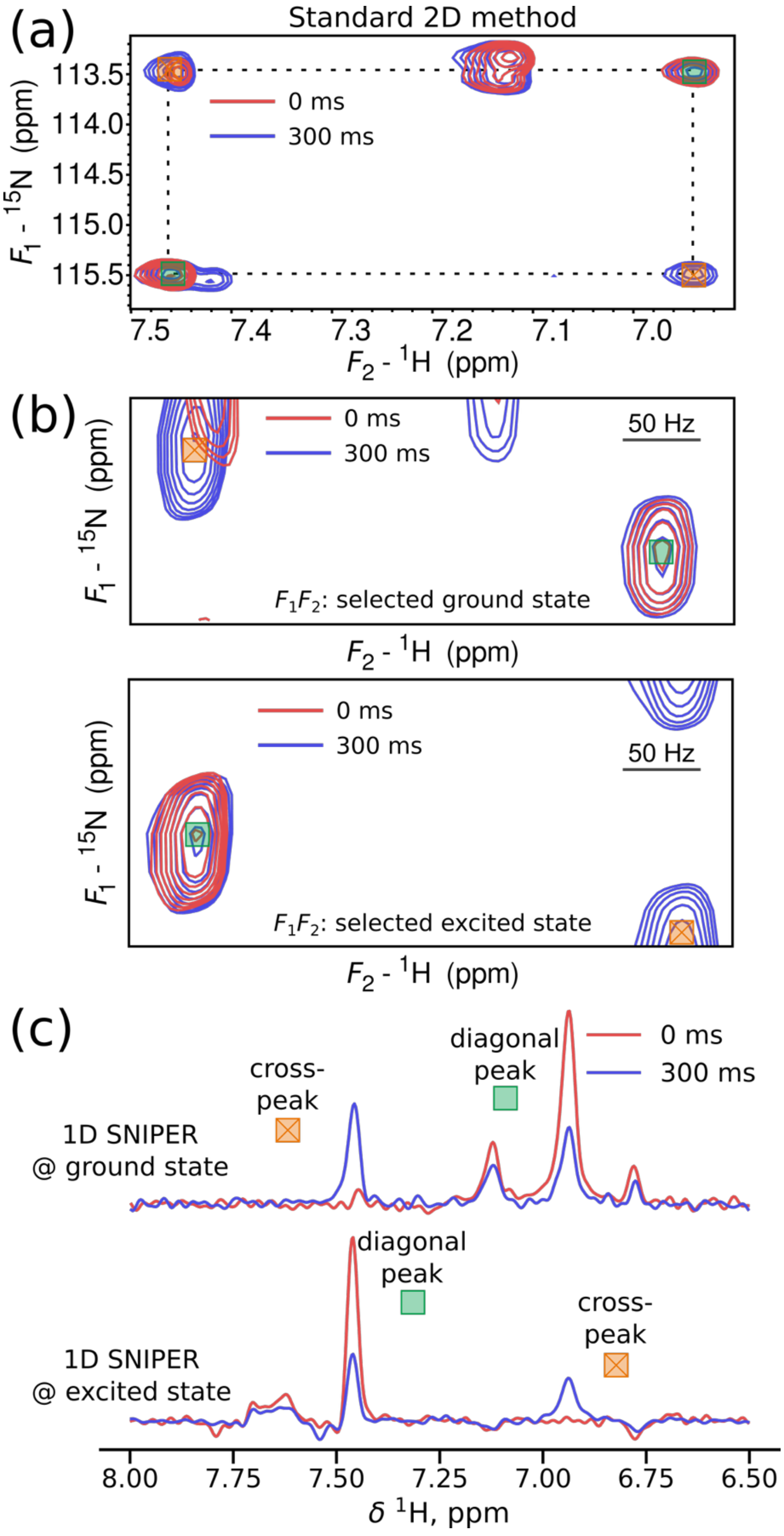

Figure S30. *zz*-exchange experiments focusing on residue N16 of SH3GL3-SH3. (a) Part of the standard *zz*-exchange 2D spectra, overlaying mixing times 0 (red) and 300 ms (blue). (b) Part of the F_1F_2-selective experiment spectra, selecting for the ^{15}N chemical shifts of each form, overlaying mixing times 0 (red) and 300 ms (blue). (c) 1D SNIPER spectra, selecting for the ^{15}N chemical shifts of each form. Red spectra are at 0 ms mixing time, blue spectra at 300 ms mixing time. Throughout, diagonal peaks are marked with green squares, cross-peaks with crossed orange squares.

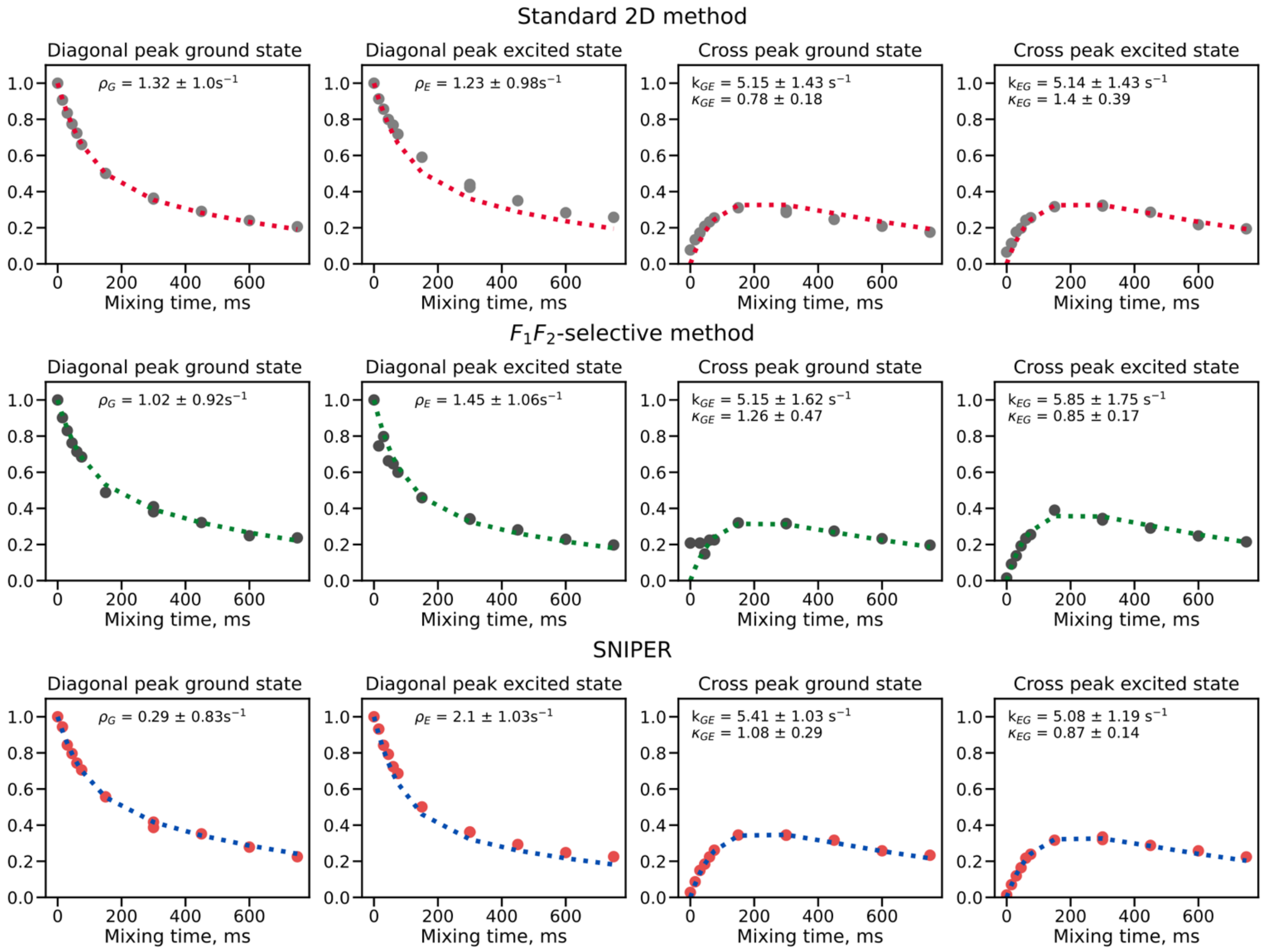

Figure S31. Intensity profiles as a function of mixing time for residue N16, obtained via (a) the standard zz-exchange 2D method, (b) the F_1F_2-selective method, or (c) the 1D SNIPER method. The numbers inside the plot refer to the ^{15}N R_1 values of each form (ρ_G and ρ_E), forward and backward exchange rates (k_{GE} and k_{EG}), as well as pre-factors that account for differential relaxation weighting of the ground and excited states (κ_{GE} and κ_{EG}).

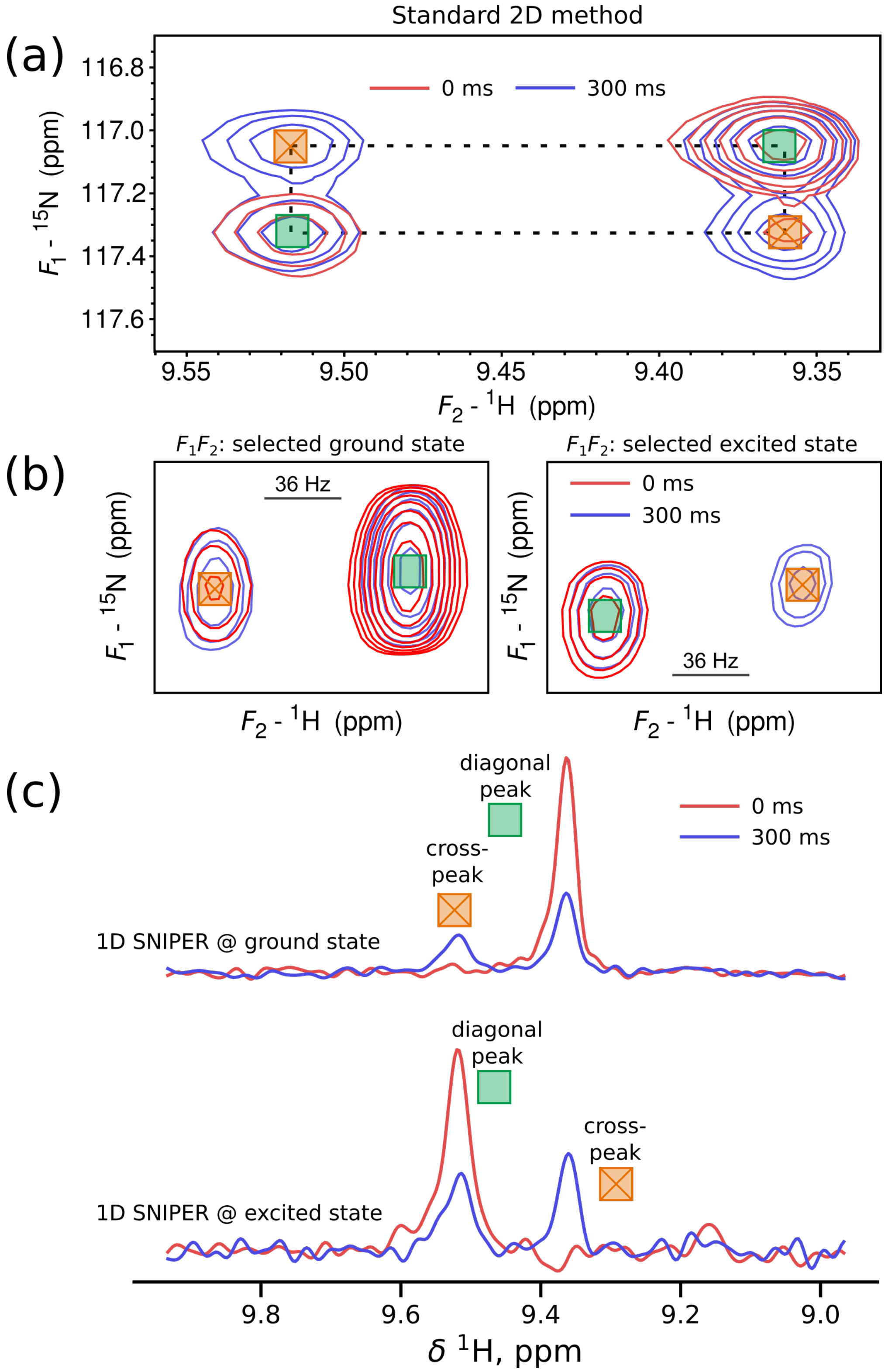

Figure S32. *zz*-exchange experiments focusing on residue C5 of SH3GL3-SH3. (a) Part of the standard *zz*-exchange 2D spectra, overlaying mixing times 0 (red) and 300 ms (blue). (b) Part of the F_1F_2-selective experiment spectra, selecting for the ^{15}N chemical shifts of each form, overlaying mixing times 0 (red) and 300 ms (blue). (c) 1D SNIPER spectra, selecting for the ^{15}N chemical shifts of each form. Red spectra are at 0 ms mixing time, blue spectra at 300 ms mixing time. Throughout, diagonal peaks are marked with green squares, cross-peaks with crossed orange squares.

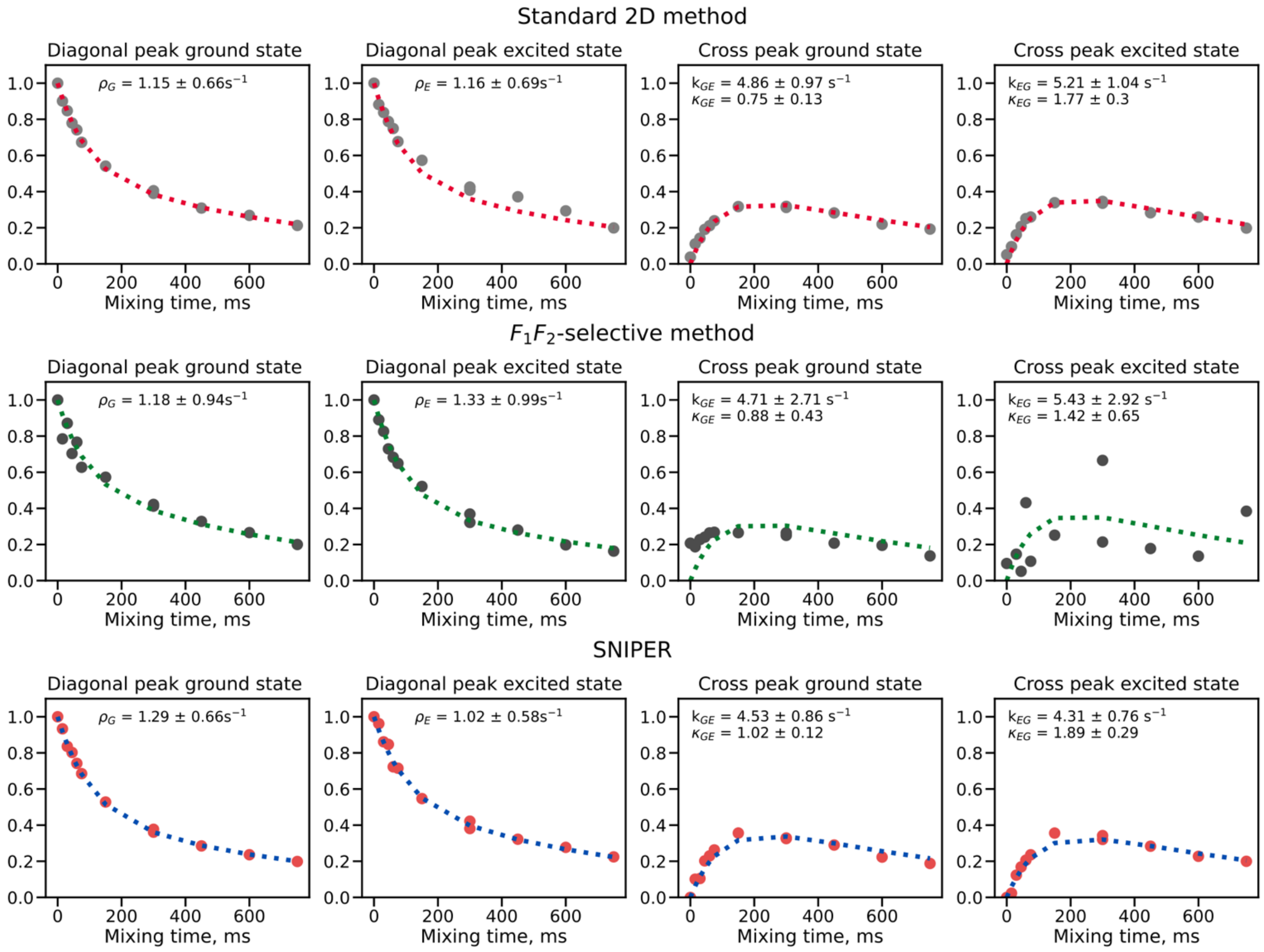

Figure S33. Intensity profiles as a function of mixing time for residue C5, obtained via (a) the standard zz-exchange 2D method, (b) the F_1F_2-selective method, or (c) the 1D SNIPER method. The numbers inside the plot refer to the ^{15}N R_1 values of each form (ρ_G and ρ_E), forward and backward exchange rates (k_{GE} and k_{EG}), as well as pre-factors that account for differential relaxation weighting of the ground and excited states (κ_{GE} and κ_{EG}).

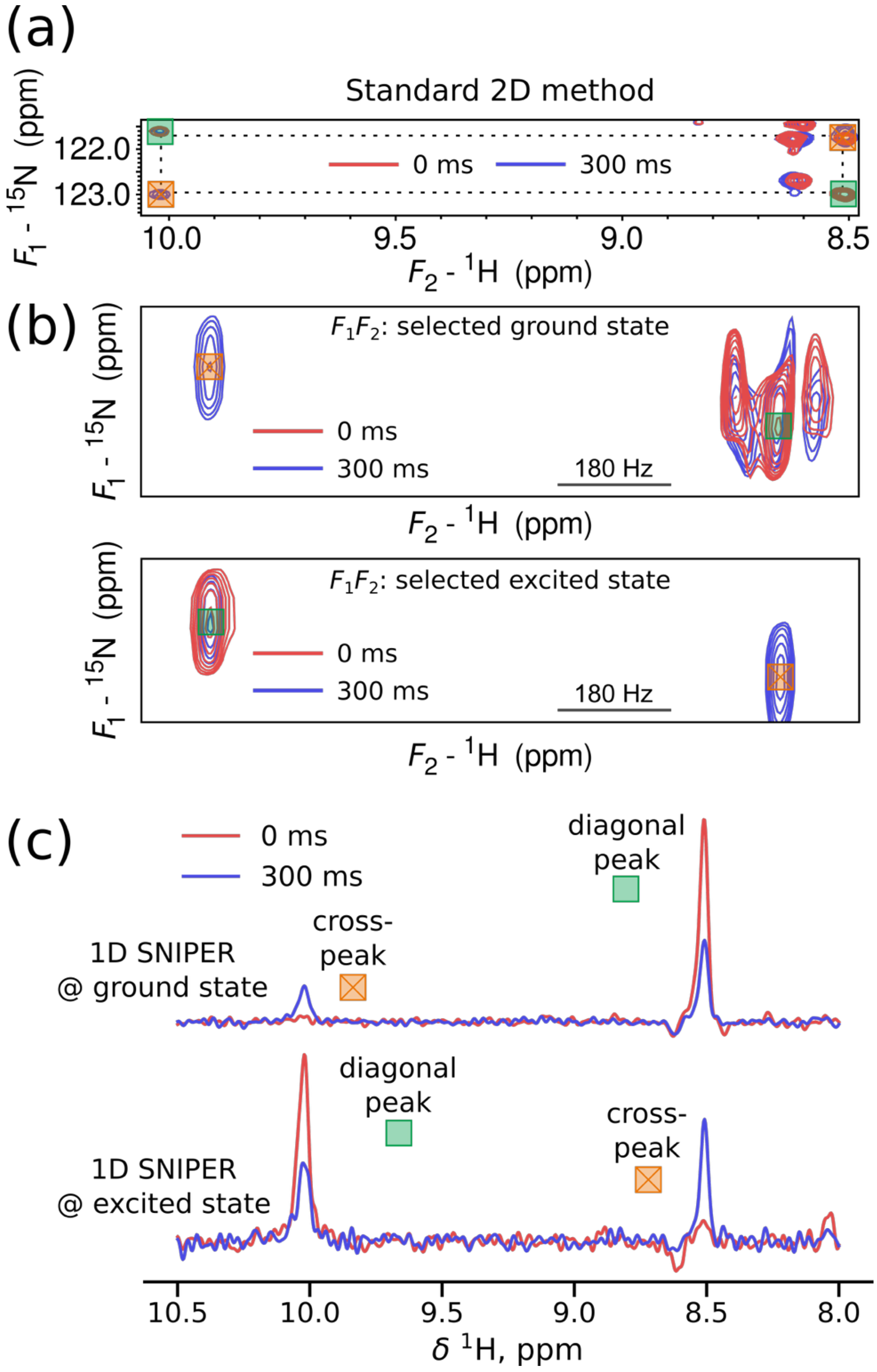

Figure S34. *zz*-exchange experiments focusing on residue Q33 of SH3GL3-SH3. (a) Part of the standard *zz*-exchange 2D spectra, overlaying mixing times 0 (red) and 300 ms (blue). (b) Part of the F_1F_2-selective experiment spectra, selecting for the ^{15}N chemical shifts of each form, overlaying mixing times 0 (red) and 300 ms (blue). (c) 1D SNIPER spectra, selecting for the ^{15}N chemical shifts of each form. Red spectra are at 0 ms mixing time, blue spectra at 300 ms mixing time. Throughout, diagonal peaks are marked with green squares, cross-peaks with crossed orange squares.

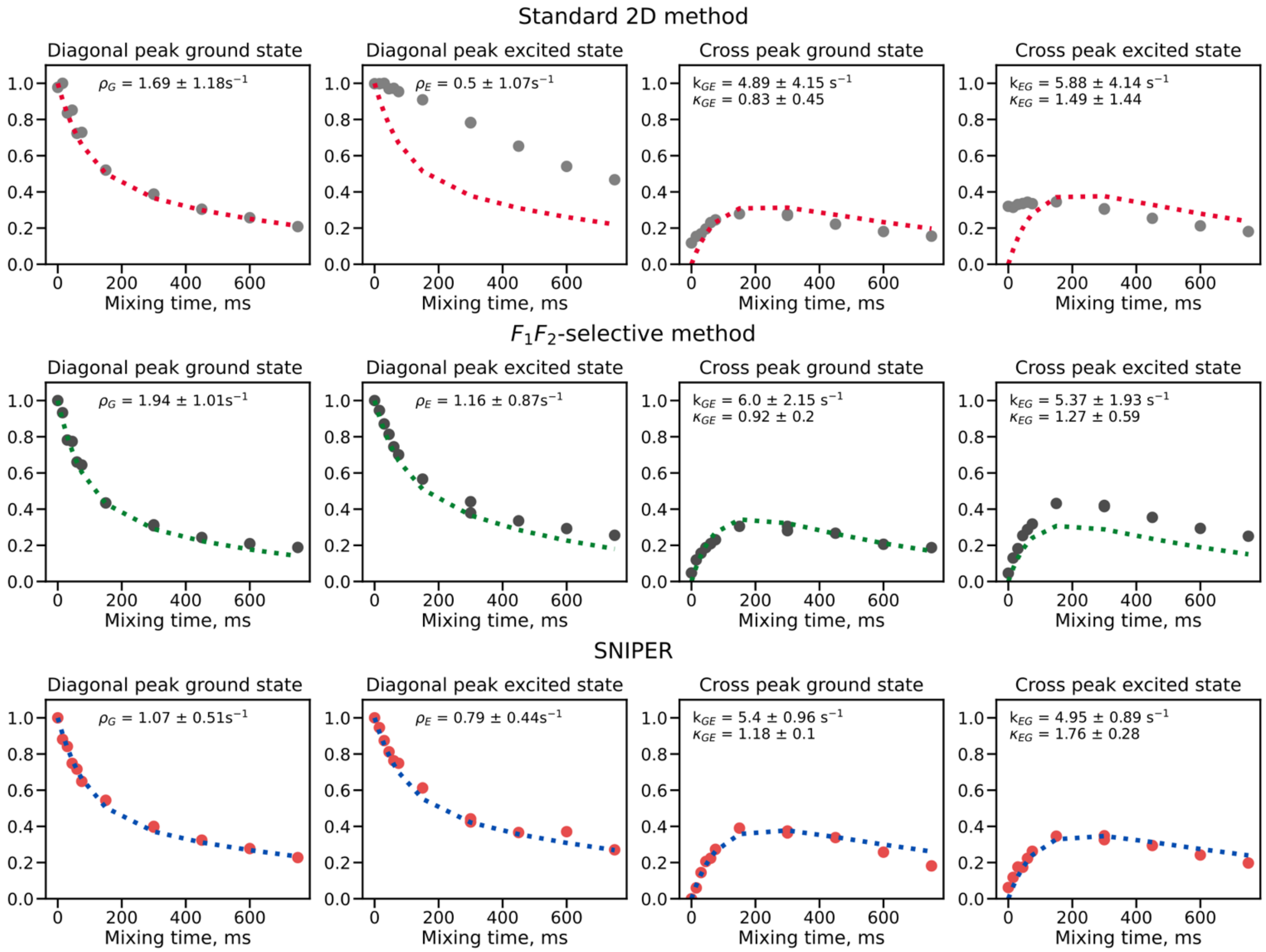

Figure S35. Intensity profiles as a function of mixing time for residue Q33, obtained via (a) the standard zz-exchange 2D method, (b) the F_1F_2-selective method, or (c) the 1D SNIPER method. The numbers inside the plot refer to the ^{15}N R_1 values of each form (ρ_G and ρ_E), forward and backward exchange rates (k_{GE} and k_{EG}), as well as pre-factors that account for differential relaxation weighting of the ground and excited states (κ_{GE} and κ_{EG}).

6. Practical SNIPER setup

6.1. Setup guidelines

A first step is to record a standard ^{1}H-^{15}N HSQC with sufficiently high resolution in F_1 to ascertain the exact frequency of each ^{1}H-^{15}N cross-peak and the proximity of the neighboring peaks.

The **SNIPER_N15sele** pulse sequence is a version of SNIPER without relaxation block and with a t1 incrementation step for 2D detection. It allows for the setup of experimental parameters and for verification of clean ^{15}N selective excitation. The sequence can be set up as a 1D or 2D experiment, which is controlled by setting the loop counter L2 to 1 or 2, respectively. For 2D detection, the States-TPPI quadrature detection is used by default.

The ^{1}H offset is set in cnst40 (in ppm), the ^{15}N offset is controlled using O3 (in Hz) or O3P (in ppm). Both should be set to the selected ^{1}H-^{15}N correlation of interest. The ^{1}H O1 offset is set to the water signal as usual. The number of complex time domain points (= half of the usual total time domain points parameter TD) is set by the loop counter L3. Assuming a ^{15}N R_2 relaxation rate constant of 8 s^{-1}, a 340 µM protein concentration and 16 complex time domain points (32 total time domain points), 128 transients are usually sufficient to reach enough signal-to-noise ratio for this experiment.

One can either choose HaHa or rSFPT for polarization transfer. Standardly, HaHa is performed in the sequence. Defining the optional pulse program flag ‘SFPT1’ replaces the $^{1}H \rightarrow ^{15}N$ polarization transfer step by rSFPT, the flag ‘SFPT2’ does so for the $^{15}N \rightarrow ^{1}H$ polarization transfer. The ^{1}H and ^{15}N power level of the HaHa elements are controlled by cnst8 (in Hz), those of rSFPT by cnst6 (in Hz). These are usually set to 45 Hz (= $0.5 \times ^{1}J_{NH}$).

The ^{15}N CSSF is by default applied with two ^{15}N 90° hard pulses and the ζ-delay. This delay is calculated from cnst3, which takes the distance in ^{15}N frequency (in Hz) to the nearest peak that is desired to be suppressed. If no ζ-delay is desired, cnst3 should be assigned a very high value (e.g. 10,000,000). If the pulse program flag ‘Nsele’ is set, the ^{15}N 90° shaped pulse will be included. This pulse’s shapefile is chosen via the parameter spnam63, and its duration with p63. The correct power level is automatically calculated based on the hard ^{15}N 90° pulse length and power level. The power level of ^{1}H CW_x decoupling during the ^{15}N CSSF is controlled using cnst10 (in Hz), and is usually set to 8000 Hz.

The ^{15}N 90° shaped pulse shape and duration should be chosen in such a way that its stop band (the off-resonance frequency where the excitation profile is below ca. 2% the maximum) is less than the distance to the nearest ^{15}N cross-peak that also interferes in the ^{1}H dimension. HalfGauss pulse shapes are advised for pulse lengths shorter than 100 ms, GOLEM for pulse lengths equal to or longer than 100 ms. Table S11 provides a good reference for the stop bands as a function of pulse length (assuming a ^{15}N R_2 on the order of 10 s^{-1}).

Besides ^{15}N selectivity, the ^{1}H selectivity can also be improved upon by using a ^{1}H CSSF (see section 2.3). This is implemented using the pulse sequence option ‘Hsele’. The ^{1}H 90° selective pulse shapefile is given by spnam61 (in this work, we used Sinc or HalfGauss). Its duration is controlled by p61. In our hands, the ^{1}H CSSF is usually not critical, with sufficient combined ^{1}H/^{15}N selectivity coming from the rSFPT/HaHa elements and the ^{15}N CSSF.

Table S11: Advised ^{15}N CSSF pulse shapes and lengths for given selectivities needed (Stop bands).

Shape	Pulse length (ms)	Stop band (Hz)
No ^{15}N CSSF	0	42*
Sinc	5	42
Sinc	10	36
Sinc	15	27
HalfGauss	50	23
HalfGauss	80	15
HalfGauss	100	13
GOLEM	100	11
GOLEM	125	9
GOLEM	150	8
GOLEM	200	6

* ^{15}N selectivity arising from the selective polarization transfer alone. ^{15}N excitation side-bands of ca. 10% will be found at 62-110 Hz offsets.

When satisfactory selectivity is observed in 2D mode for a given ^{1}H-^{15}N correlation, the relaxation-encoding experiments can be set up.

1D SNIPER R1 experiment uses the pulse program **SNIPER_N15R1**. It uses all the same optional flags and pulse sequences parameters as the SNIPER_N15sele program. The experiment is set up as a pseudo2D experiment. Loop counter L6 and TD1 should be set to the number of delay data points in the experiment. The relaxation encoding is provided as a loop counter list (vclist), which should contain randomized loop counter values. The actual relaxation encoding delay is then d24*2*vc, with d24 typically set to 20-40 ms, depending on the expected ^{15}N R_1. Ideally, the maximum relaxation encoding delay should aim for a signal intensity of ca. 37% (*i.e.*, e^{-1}) of the shortest delay (*i.e.*, compared to vc = 0). The first loop count in the vclist should always be 0 for proper pulse sequence functioning. The number of transients is set so that sufficient signal-to-noise is retained for the maximum delay.

The 1D SNIPER $R_{1\rho}$ experiment uses the pulse program **SNIPER_N15R1rho**. It uses all the same optional flags and pulse sequences parameters as the SNIPER_N15sele program. The experiment is set up as a pseudo2D experiment. Loop counter L6 and TD1 should be set to the number of delay data points in the experiment. Just as the R_1 experiment, the relaxation encoding is provided as a loop counter list (vclist), which should contain randomized loop counter values. The actual relaxation encoding delay is also for this experiment d24*2*vc, with d24 typically set to 5-9 ms, depending on the expected ^{15}N R_2. Ideally, the maximum relaxation encoding delay should aim for a signal intensity of ca. 37% (*i.e.*, e^{-1}) of the shortest delay (*i.e.*, compared to vc = 0). The first loop count in the vclist should always be 0 for proper pulse sequence functioning. For correct functioning of the constant-time $R_{1\rho}$ element, it is crucial to set cnst18 to the maximum loop number in the vclist. The spin-lock power level is set using

cnst11 (in Hz). The delay d23 is used for relaxation-dispersion experiments and provides an additional equilibration of magnetization between different forms of exchanging species flanking the relaxation element for systems featuring exchange. It can be set to ca. 5 ms for k_{ex} ~ 200/s. The number of transients is set so that sufficient signal-to-noise is retained for the maximum delay, and is usually roughly 1.5-2 times that for the SNIPER R_1 experiment. Finally, the pulse sequence flag 'orr' allows for the use of an off-resonance $R_{1\rho}$ experimental setup. The setup takes into account a list of offsets as fq1list (Hz, relative to O3) and amplitudes (provided in valist) to modulate the 90° ^{15}N pulse power level so that the magnetization is aligned with the spin-lock at each offset. The loop counter L7 defines the number of offsets. More details on how to set it up can be found in the literature.[17]

The 1D SNIPER *zz*-exchange experiment uses the pulse program **SNIPER_N15Rex**. It uses all the same optional flags and pulse sequences parameters as the SNIPER_N15sele and SNIPER_N15R1 programs, except that it does not use a ^{15}N→^{1}H in-phase polarization transfer and thus no optional flag 'SFPT2'. Instead, it uses a reversed refocused INEPT sequence with a setup similar as in the standard ^{1}H-^{15}N HSQC.

The 1D SNIPER ^{1}H-^{15}N nOe experiment uses the pulse program **SNIPER_N15nOe**. It uses all the same optional flags and pulse sequences parameters as the SNIPER_N15sele, except that it does not contain a ^{1}H→^{15}N in-phase polarization transfer and thus no optional flag 'SFPT1'. The experiment is set up as a pseudo2D experiment. The TD1 parameter should be set to 2. In the first increment of the pseudo2D, the experiment is run without ^{1}H saturation, and the second with it. The total duration of ^{1}H saturation (or recovery) time is controlled by the loop counter L8, with the total saturation (or recovery) time equal to L8*22 ms. Typically, L8 is set to 230-370 (resulting in 5-8 s in total). The interscan delay d1 should be at least 2 s. The number of transients is set so that sufficient signal-to-noise is obtained, and is typically about twice the number needed for SNIPER R_1. A way to run a quick experiment to assess this is to set cnst12 to 0 to switch off the ^{15}N 90° hard pulse before the d1 delay and set L8 to 1 to reduce experimental time. Note that the intensity of the actual experiment will be approximately 1.5-2 times greater due to full T_1-relaxation between scans.

6.2. Pulse sequence Bruker codes

On the following pages, the above-mentioned Bruker pulse programs are provided.

6.2.1. SNIPER_N15sele

```
;SNIPER_N15sele
/****************************************************************
SNIPER revealing ^{15}N and ^{1}H selectivity on per resonances basis using Hartmann-Hahn 15N-edited 1H (2D) spectra
quadrature detection using States-TPPI
with optional SFPT for selective polarization in 'out' (flag SFPT1) and 'back' (flag SFPT2) transfer
with optional 15N (flag Nsele) and 1H (flag Hsele) CSSF during spin-locked 1H and 15N mag. respectively
with pulse-delay 1H alignment with CWx during 15N CSSF
with watergate before readout
written by WA 2024/12/31
****************************************************************/
; zgoptns: SFPT1, SFPT2, Nsele, Hsele


#include <Avance.incl>
#include <Grad.incl>
#include <Delay.incl>


"d11=30m"
"d16=200u" ; long homospoil rec.
"d17=100u" ; short homospoil rec.

"d0=0"
"in0=inf1/2"

;--- calc HaHa transfer and delay for CSSF
"cnst9=90" ; J_NH for HH
"DELTA=1s/(cnst9)" ; 1/J_NH for HH

#if defined (SFPT1)||defined (SFPT2)
"p15=DELTA/sqrt(2)" ; pulse duration for SFPT
"p6=500*500/(cnst6+0.01)" ; calculate SFPT spin-lock pulse length (us)
```

```
"spw17=p1*p1*plw1/(p6*p6*integfac17*integfac17)" ; calculate 1H SFPT spin-lock power level (W)
"spw18=p7*p7*plw7/(p6*p6*integfac18*integfac18)" ; calculate 15N SFPT spin-lock power level (W)
#endif

"p18=DELTA"  ; pulse duration for HaHa
"p8=500*500/(cnst8+0.01)" ; calculate HaHa spin-lock pulse length (us)
"spw19=p1*p1*plw1/(p8*p8*integfac19*integfac19)" ; calculate 1H HaHa spin-lock power level (W)
"spw20=p7*p7*plw7/(p8*p8*integfac20*integfac20)" ; calculate 15N HaHa spin-lock power level (W)

;-- cleaning element and selective pulse calibration
"DELTA3=1/(cnst3*4+0.0001)" ;zeta delay to remove unwanted peak cnst3 (delta, Hz) away from the resonance
;-- 15N CSSF
#ifdef Nsele
"spw63=p7*p7*plw7/(p63*p63*integfac63*integfac63)"
"spoal63=1"
#endif
;-- 1H CSSF
#ifdef Hsele
"spw61=p1*p1*plw1/(p61*p61*integfac61*integfac61)"
"spoal61=1"
#endif

;--- calc 13C pulses
"p4=23.7u*600/bf1"
"plw4=p3*p3*plw2/(p4*p4)"
"spw4=plw4"
"cnst22=56" ; offset Ca
"cnst21=176" ; offset C'
"spoffs4=bf2*((cnst22-cnst21)/1000000)" ; move carrier from C' to Ca

;----- calc power levels for Spin-Lock using SL power defined by cnst10 (1H) and cnst11 (15N)
"p10=500*500/(cnst10+0.01)" ;calculate pulse length for 1H CWx during 15N zeta-purge and relax
"plw10=p1*p1*plw1/(p10*p10)";calculate spin-lock power level for 1H CWx during 15N zeta-purge and relax
"d22=(p10*2/PI)-(p1*4/PI)" ; 1st delay to align 1H along X before CWx
"d29=p1*2/PI-2u" ; 2nd delay to align 1H along X before CWx
```

```
;---- watergate + readout
"p5=1500u*600/bf1" ;shaped pulse on water (1.5m@14.2T)
"spw5=p1*p1*plw1/(p5*p5*integfac5*integfac5)"
"p31=250u" ; 1 kHz dec. on 15N
"plw31=p7*p7*plw7/(p31*p31)" ; calculate 15N decoupling power level for 1kHz dec (p31=250u)


1 ze
1m
2 2m do:f3
1m do:f2
1u fq=0:f3
d11
3 3m
4 2m do:f3
1m do:f2
10u pl1:f1
10u pl7:f3

;--------- d1 recovery delay---------
d1
1m UNBLKGRAD
(p7 ph0):f3
p22:gp1
d16 pl0:f1

;------ start selective/non-selective 1H excitation
1u fq=cnst40(bf ppm):f1
1u pl1:f1

#ifdef Hsele
1u pl7:f3
(p7 ph3):f3
1u pl31:f3
1u cw:f3
(p61:sp61 ph0):f1
```

```
1u do:f3
1u pl7:f3
(p7 ph1):f3
1u pl1:f1
(p1 ph2):f1
p22:gp11
d16
(p1 ph0):f1
#else /*Hsele*/
1u fq=0:f1
(p5:sp5 ph2):f1
1u pl1:f1
1u fq=cnst40(bf ppm):f1
(p1 ph0):f1
#endif /*Hsele*/

;--- start HaHa or SFPT 1H to 15N
#ifdef SFPT1
(p15:sp17 ph7):f1
(p15:sp18 ph8):f3
#else /*SFPT1*/
(center (p18:sp19 ph7):f1 (p18:sp20 ph8):f3)
#endif /*SFPT1*/
1u pl1:f1
1u pl7:f3

;--- purge HzNz
(p7 ph1):f3
1u
(p1 ph2):f1
p22:gp2
d16 pl1:f1

;---- align 1H on x before CWx, start purge element
  (p1 ph11):f1
  d22
```

```
  (p1 ph0):f1
  (p1*2 ph11):f1
  d29
  1u pl10:f1
  1u cw:f1
#ifdef Nsele
(p63:sp63 ph13):f3
#else
 (p7 ph13):f3
#endif /*Nsele**/
DELTA3
  1u pl7:f3
  (p7 ph2):f3
  1u do:f1
  1u pl1:f1
  d29
  (p1*2 ph12):f1
  (p1 ph0):f1
  d22
  (p1 ph12):f1
p22:gp3
d16
1u pl1:f1
1u pl4:f2
1u pl7:f3
;---- t1-encoding with optional Echo-Antiecho ('SE' flag), otherwise States-TPPI
(p7 ph18):f3
if "d0-p4*2-4u > 2u"
{
"DELTA2=d0-p4*2-4u"
1u gron0
DELTA2*0.5
1u groff
1u gron0*-1
DELTA2*0.5
1u groff
```

```
(center (p1*2 ph0):f1 (p4*2 ph0 p4*2:sp4 ph0):f2)
DELTA2
4u
}
else
{
1u gron0
d0*0.5
1u groff
1u gron0*-1
d0*0.5
1u groff
(p1*2 ph0):f1
d0
4u
}
;--- end t1-encoding, start back-transfer
#ifdef SFPT2
(p15:sp18 ph6):f3
(p15:sp17 ph5):f1
#else /*SFPT2*/
(center (p18:sp19 ph5):f1 (p18:sp20 ph6):f3)
#endif /*SFPT2*/
1u pl1:f1
1u pl7:f3
1u fq=0:f1
p25:gp5
d17 pl0:f1
(p5:sp5 ph0):f1
1u pl1:f1
(p1*2 ph2):f1
1u pl0:f1
(p5:sp5 ph0):f1
p25:gp5
"d18=d17-2u"
d18
```

```
;---- start acqu
999 1u pl31:f3
 1u BLKGRAD
go=2 ph31 cpds3:f3
500u do:f3
500u do:f2
d11 wr #0 if #0 zd
 1m ip18
 lo to 3 times l2
 500u iu0
 500u id0
 lo to 4 times l3
1m do:f3
exit

ph0=0
ph1=1
ph2=2
ph3=3

ph7=1 3
ph8=2 2 0 0
ph13=0 0 0 0 2 2 2 2
ph6=0 0 0 0 0 0 0 0 2 2 2 2 2 2 2 2
ph5=0 0 0 0 0 0 0 0 0 0 0 0 0 0 0 0
    2 2 2 2 2 2 2 2 2 2 2 2 2 2 2 2

ph18=3

ph11=1 1 3 3
ph12=3 3 1 1

ph31=0 2 2 0 2 0 0 2 2 0 0 2 0 2 2 0
     2 0 0 2 0 2 2 0 0 2 2 0 2 0 0 2
```

```
;-------------NOTES---------------------
;o1p=4.7 ppm
;o2p=176 ppm (CO)
;o3p=set to resonance of interest, ppm
;NS=32*n

; pulses and power levels
;pl0: 120 dB
;p1: 90 deg hard 1H pulse @pl1
;pl1: 1H 90 deg
;p3: 90 deg hard 13C pulse @pl2
;pl2: 13C 90 deg
;p4: 90 deg selective on 13Ca @pl4
;pl4: 13Ca selective 90 deg
;p7: 90 deg hard 15N pulse @pl7
;pl7: 15N 90 deg

;spnam4 Squa100.1000
;spoal4 0.5

;p5: water flip-back pulse duration
;spnam5 Sinc1.1000
;spoal5 0.5

;p6: rSFPT spin-lock pulse length corresponding to power level cnst6 (us)
;p8: HaHa spin-lock pulse length corresponding to power level cnst8 (us)

;p15: duration of SFPT transfer spin-lock
;p18: duration of HaHa transfer spin-lock

;p61: 90 deg 1H selective pulse (1-20ms) 1H CSSF
;p63: 90 deg 15N selective pulse (10-200ms) 15N CSSF

;p10: calculate pulse length for 1H CWx during 15N CSSF
;pl10: calculate spin-lock power level for 1H CWx during 15N CSSF
```

;sp17: rSFPT 1H spin-lock power level (W)
;spnam17: Squa100.1000
;sp18: rSFPT 15N spin-lock power level (W)
;spnam18: Squa100.1000
;sp19: HaHa 1H spin-lock power level (W)
;spnam19: Squa100.1000
;sp20: HaHa 15N spin-lock power level (W)
;spnam20: Squa100.1000
;sp61: selective Hz->Hxy for initial excitation
;spnam61 Sinc1.1000
;sp63: selective Nz->Nxy during zeta element
;spnam63 GOLEM.1000

;spoal16 0.5
;spoal17 0.5
;spoal18 0.5
;spoal19 0.5
;spoal20 0.5
;spoal61 1
;spoal63 1

;CPDPRG3: garp4 (aq 15N decoupling)
;pcpd3: 15N decoupling (250u @pl31)
;pl31: 15N decoupling power

;--- cnsts
;cnst3: 15N offset (Hz) of unwanted peak to be removed during zeta delay
;cnst6: for calculation of power level for rSFPT transfer (45 Hz)
;cnst8: for calculation of power level for HaHa transfer (45 Hz)
;cnst9: for calculation of delay for HaHa transfer (90 Hz)
;cnst10: 1H SL power during 15N CSSF or relax (Hz)
;cnst40: 1H offset of selected resonance (ppm)

;d22: 1st delay to align CWx with spin-lock
;d29: 2nd delay to align CWx align with spin-lock

;loops and lists
;l6: number of delays in vclist

; gradients
;p22: purge Nz (1m)
;p25: watergate (500u)

;for z-only gradients
;gpz1: -66%
;gpz2: 45%
;gpz3: -73%
;gpz4: 57%
;gpz5: 83%
;gpz11: 51%

6.2.2. SNIPER_N15R1

```
;SNIPER_N15R1
/*****************************************************************
SNIPER ^{15}N R1 on per resonances basis using Hartmann-Hahn 15N-edited 1H (1D) spectra
with optional rSFPT for selective polarization in 'out' (flag SFPT1) and 'back' (flag SFPT2) transfer
with optional 15N (flag Nsele) and 1H (flag Hsele) CSSF during spin-locked 1H and 15N mag.
with pulse-delay 1H alignment with CWx during 15N CSSF and relaxation elements
with swapped order of zeta element (moved in front of relax block according to Sugase and co-workers, DOI 10.1007/s10858-017-0097-6)
with watergate before readout
tau_eq is performed during CWx before and after relax
written by WA 2024/12/31
*****************************************************************/
; zgoptns: SFPT1, SFPT2, Nsele, Hsele

#include <Avance.incl>
#include <Grad.incl>
#include <Delay.incl>

define list<loopcounter> vc    = <$VCLIST>  ;loopcounter list for relax delays


"d11=30m"
"d16=200u" ; long homospoil rec.
"d17=100u" ; short homospoil rec.
"d18=d17-2u"


;--- calc HaHa transfer and delay for zeta-purge
"cnst9=90" ; J_NH for HH
"DELTA=1s/(cnst9)" ; 1/J_NH for HH

#if defined (SFPT1)||defined (SFPT2)
"p15=DELTA/sqrt(2)" ; pulse duration for SFPT
"p6=500*500/(cnst6+0.01)" ; calculate SFPT spin-lock pulse length (us)
```

```
"spw17=p1*p1*plw1/(p6*p6*integfac17*integfac17)" ; calculate 1H SFPT spin-lock power level (W)
"spw18=p7*p7*plw7/(p6*p6*integfac18*integfac18)" ; calculate 15N SFPT spin-lock power level (W)
#endif


"p18=DELTA"  ; pulse duration for HaHa
"p8=500*500/(cnst8+0.01)" ; calculate HaHa spin-lock pulse length (us)
"spw19=p1*p1*plw1/(p8*p8*integfac19*integfac19)" ; calculate 1H HaHa spin-lock power level (W)
"spw20=p7*p7*plw7/(p8*p8*integfac20*integfac20)" ; calculate 15N HaHa spin-lock power level (W)

;-- cleaning element and selective pulse calibration
"DELTA3=1/(cnst3*4+0.0001)" ;zeta delay to remove unwanted peak cnst3 (delta, Hz) away from the resonance
#ifdef Nsele
"spw63=p7*p7*plw7/(p63*p63*integfac63*integfac63)"
"spoal63=1"
#endif

#ifdef Hsele
"spw61=p1*p1*plw1/(p61*p61*integfac61*integfac61)"
"spoal61=1"
#endif

;--- calc 13C pulses
"p4=23.7u*600/bf1"
"plw4=p3*p3*plw2/(p4*p4)"
"spw4=plw4"
"cnst22=56" ; offset Ca
"cnst21=176" ; offset C'
"spoffs4=bf2*((cnst22-cnst21)/1000000)" ; move carrier from C' to Ca

;----- calc power levels for Spin-Lock using SL power defined by cnst10 (1H)
"p10=500*500/(cnst10+0.01)" ;calculate pulse length for 1H CWx during 15N zeta-purge and relax
"plw10=p1*p1*plw1/(p10*p10)";calculate spin-lock power level for 1H CWx during 15N zeta-purge and relax
"d22=(p10*2/PI)-(p1*4/PI)" ; 1st delay to align 1H along X before CWx
"d29=p1*2/PI-2u" ; 2nd delay to align 1H along X before CWx
```

```
;----- calc 1HN inversion during N15 R1 relax element (applied every d25: 5-40ms)
"p13=1600u*600/bf1"
"spw13=4*p1*p1*plw1/(p13*p13*integfac13*integfac13)"
"spoffs13=(2340*bf1/600)/1000000"
"d25=d24-p13*0.5-p4*4"

;------ watergate + detection
"p5=1500u*600/bf1" ;shaped pulse on water (1.5m@14.2T)
"spw5=p1*p1*plw1/(p5*p5*integfac5*integfac5)"
"p31=250u" ; 1 kHz dec. on 15N
"plw31=p7*p7*plw7/(p31*p31)" ; calculate 15N decoupling power level for 1kHz dec (p31=250u)


1 ze
1m
2 2m do:f3
1m do:f2
1u fq=0:f3
d11
3 3m
4 2m do:f3
1m do:f2
10u pl1:f1
10u pl7:f3

;--------- d1 recovery delay---------
d1
1m UNBLKGRAD
(p7 ph0):f3
p22:gp1
d16 pl0:f1

;------ start selective/non-selective 1H excitation
1u fq=cnst40(bf ppm):f1
1u pl1:f1
```

```
#ifdef Hsele
1u pl7:f3
(p7 ph3):f3
1u pl31:f3
1u cw:f3
(p61:sp61 ph0):f1
1u do:f3
1u pl7:f3
(p7 ph1):f3
1u pl1:f1
(p1 ph2):f1
p22:gp11
d16
(p1 ph0):f1
#else /*Hsele*/
1u fq=0:f1
(p5:sp5 ph2):f1
1u pl1:f1
1u fq=cnst40(bf ppm):f1
(p1 ph0):f1
#endif /*Hsele*/

;--- start HaHa or SFPT 1H to 15N
#ifdef SFPT1
(p15:sp17 ph7):f1
(p15:sp18 ph8):f3
#else /*SFPT1*/
(center (p18:sp19 ph7):f1 (p18:sp20 ph8):f3)
#endif /*SFPT1*/
1u pl1:f1
1u pl7:f3

;--- purge HzNz
(p7 ph1):f3
1u
(p1 ph2):f1
```

```
p22:gp2
d16 pl1:f1

;---- align 1H on x before CWx, start purge element
  (p1 ph11):f1
  d22
  (p1 ph0):f1
  (p1*2 ph11):f1
  d29
  1u pl10:f1
  1u cw:f1
#ifdef Nsele
(p63:sp63 ph13):f3
#else
 (p7 ph13):f3
#endif /*Nsele**/
DELTA3
  1u pl7:f3
  (p7 ph2):f3
  1u do:f1
  1u pl1:f1
  d29
  (p1*2 ph12):f1
  (p1 ph0):f1
  d22
  (p1 ph12):f1
p22:gp3
d16
1u pl1:f1
1u pl4:f2
1u pl7:f3
;---- Nz relax
if "vc==0" goto 601
1u fq=0:f1

600 d25*0.5
```

```
1u pl4:f2
(p4*2 ph0 p4*2:sp4 ph0):f2
d25*0.5
(p13:sp13 ph0):f1
d25*0.5
1u pl4:f2
(p4*2 ph0 p4*2:sp4 ph0):f2
d25*0.5
lo to 600 times vc

601 1u pl7:f3
1u pl1:f1
p22:gp4
d16

;--- back-transfer
1u fq=cnst40(bf ppm):f1
(p7 ph1):f3
#ifdef SFPT2
(p15:sp18 ph6):f3
(p15:sp17 ph5):f1
#else /*SFPT2*/
(center (p18:sp19 ph5):f1 (p18:sp20 ph6):f3)
#endif /*SFPT2*/
1u pl1:f1
1u pl7:f3
1u fq=0:f1
p25:gp5
d17 pl0:f1
(p5:sp5 ph0):f1
1u pl1:f1
(p1*2 ph2):f1
1u pl0:f1
(p5:sp5 ph0):f1
p25:gp5
"d18=d17-2u"
```

```
d18


;---- start acqu
999 1u pl31:f3
 1u BLKGRAD
go=2 ph31 cpds3:f3
500u do:f3
500u do:f2
d11 wr #0 if #0 zd
 500u vc.inc
 lo to 3 times l6
 500u vc.res
1m do:f3
exit

ph0=0
ph1=1
ph2=2
ph3=3

ph7=1 3
ph8=2 2 0 0
ph13=0 0 0 0 2 2 2 2
ph6=0 0 0 0 0 0 0 0 2 2 2 2 2 2 2 2
ph5=0 0 0 0 0 0 0 0 0 0 0 0 0 0 0 0
    2 2 2 2 2 2 2 2 2 2 2 2 2 2 2 2

ph11=1 1 3 3
ph12=3 3 1 1

ph31=0 2 2 0 2 0 0 2 2 0 0 2 0 2 2 0
     2 0 0 2 0 2 2 0 0 2 2 0 2 0 0 2

;-------------NOTES----------------------
;o1p=4.7 ppm
```

;o2p=176 ppm (CO)
;o3p=set to resonance of interest, ppm
;NS=32*n

; pulses and power levels
;pl0: 120 dB
;p1: 90 deg hard 1H pulse @pl1
;pl1: 1H 90 deg
;p3: 90 deg hard 13C pulse @pl2
;pl2: 13C 90 deg
;p4: 90 deg selective on 13Ca @pl4
;pl4: 13Ca selective 90 deg
;p7: 90 deg hard 15N pulse @pl7
;pl7: 15N 90 deg

;spnam4 Squa100.1000
;spoal4 0.5

;p5: water flip-back pulse duration
;spnam5 Sinc1.1000
;spoal5 0.5

;p6: rSFPT spin-lock pulse length corresponding to power level cnst6 (us)
;p8: HaHa spin-lock pulse length corresponding to power level cnst8 (us)

;p15: duration of SFPT transfer spin-lock
;p18: duration of HaHa transfer spin-lock

;p61: 90 deg 1H selective pulse (1-20ms) 1H CSSF
;p63: 90 deg 15N selective pulse (10-200ms) 15N CSSF

;p10: calculate pulse length for 1H CWx during 15N CSSF
;pl10: calculate spin-lock power level for 1H CWx during 15N CSSF

;sp17: rSFPT 1H spin-lock power level (W)
;spnam17: Squa100.1000

```
;sp18: rSFPT 15N spin-lock power level (W)
;spnam18: Squa100.1000
;sp19: HaHa 1H spin-lock power level (W)
;spnam19: Squa100.1000
;sp20: HaHa 15N spin-lock power level (W)
;spnam20: Squa100.1000
;sp61: selective Hz->Hxy for initial excitation
;spnam61 Sinc1.1000
;sp63: selective Nz->Nxy during zeta element
;spnam63 GOLEM.1000

;spoal16 0.5
;spoal17 0.5
;spoal18 0.5
;spoal19 0.5
;spoal20 0.5
;spoal61 1
;spoal63 1

;CPDPRG3: garp4 (aq 15N decoupling)
;pcpd3: 15N decoupling (250u @pl31)
;pl31: 15N decoupling power

;--- cnsts
;cnst3: 15N offset (Hz) of unwanted peak to be removed during zeta delay
;cnst6: for calculation of power level for rSFPT transfer (45 Hz)
;cnst8: for calculation of power level for HaHa transfer (45 Hz)
;cnst9: for calculation of delay for HaHa transfer (90 Hz)
;cnst10: 1H SL power during 15N CSSF or relax (Hz)
;cnst40: 1H offset of selected resonance (ppm)

;d24: relaxation delay *2*vc
;d22: 1st delay to align CWx with spin-lock
;d29: 2nd delay to align CWx align with spin-lock

;loops and lists
```

;l6: number of delays in vclist

; gradients
;p22: purge Nz (1m)
;p25: watergate (500u)

;for z-only gradients
;gpz1: -66%
;gpz2: 45%
;gpz3: -73%
;gpz4: 57%
;gpz5: 83%
;gpz11: 51%

6.2.3. SNIPER_N15R1rho

```
;SNIPER_N15R1rho
/***************************************************************
SNIPER ^{15}N R1rho on per resonances basis using Hartmann-Hahn 15N-edited 1H (1D) spectra
with optional SFPT for selective polarization in 'out' (flag SFPT1) and 'back' (flag SFPT2) transfer
with optional 15N (flag Nsele) and 1H (flag Hsele) selective excitation during spin-locked 1H and 15N mag.
with pulse-delay 1H alignment with CWx during 15N selective excitation and relaxation elements
with swapped order of zeta element (moved in front of relax block according to Sugase and co-workers, DOI 10.1007/s10858-017-0097-6)
with 15N constant time temperature compensation with CWx 1H (8kHz)
with optional off-resonance offsets using FQ1LISt and hard pulse with matched tilt-angle for 15N magnetization alignment using VALIST
(flag orr)
with watergate before readout
tau_eq is performed during CWx before and after relax
written by WA 2024/12/31
***************************************************************/
; zgoptns: SFPT1, SFPT2, Nsele, Hsele, orr

#include <Avance.incl>
#include <Grad.incl>
#include <Delay.incl>

define list<loopcounter> vc    = <$VCLIST>  ;loopcounter list for relax delays
define list<frequency> offTemp = {-48000}   ;in Hz, 15N very far off-resonance for Tcomp.

#ifdef orr
define list<amplitude> va3    = <$VALIST>  ;variable amplitude power list (in %) to attenuate plw7:f3 before and after off-resonant spin-lock
define list<frequency> offRes  = <$FQ1LIST> ;15N offset list for off-resonance dispersion (Hz)
#else /*orr*/
define list<amplitude> va3     = {100}      ;variable amplitude power list (in %) to attenuate plw7:f3 before and after off-resonant spin-lock
define list<frequency> offRes  = {0}        ;15N offset for off-resonance dispersion (Hz) (0 Hz, on-resonance)
#endif /*orr*/

"d11=30m"
"d16=200u" ; long homospoil rec.
```

```
"d17=100u" ; short homospoil rec.
"d18=d17-2u"
"l5=0"


;--- calc HaHa transfer and delay for zeta-purge
"cnst9=90" ; J_NH for HH
"DELTA=1s/(cnst9)" ; 1/J_NH for HH

#if defined (SFPT1)||defined (SFPT2)
"p15=DELTA/sqrt(2)" ; pulse duration for SFPT
"p6=500*500/(cnst6+0.01)" ; calculate SFPT spin-lock pulse length (us)
"spw17=p1*p1*plw1/(p6*p6*integfac17*integfac17)" ; calculate 1H SFPT spin-lock power level (W)
"spw18=p7*p7*plw7/(p6*p6*integfac18*integfac18)" ; calculate 15N SFPT spin-lock power level (W)
#endif

"p18=DELTA"  ; pulse duration for HaHa
"p8=500*500/(cnst8+0.01)" ; calculate HaHa spin-lock pulse length (us)
"spw19=p1*p1*plw1/(p8*p8*integfac19*integfac19)" ; calculate 1H HaHa spin-lock power level (W)
"spw20=p7*p7*plw7/(p8*p8*integfac20*integfac20)" ; calculate 15N HaHa spin-lock power level (W)

;-- cleaning element and selective pulse calibration
"DELTA3=1/(cnst3*4+0.0001)" ;zeta delay to remove unwanted peak cnst3 (delta, Hz) away from the resonance
#ifdef Nsele
"spw63=p7*p7*plw7/(p63*p63*integfac63*integfac63)"
"spoal63=1"
#endif

#ifdef Hsele
"spw61=p1*p1*plw1/(p61*p61*integfac61*integfac61)"
"spoal61=1"
#endif

;--- calc 13C pulses
"p4=23.7u*600/bf1"
"plw4=p3*p3*plw2/(p4*p4)"
```

```
"spw4=plw4"
"cnst22=56" ; offset Ca
"cnst21=176" ; offset C'
"spoffs4=bf2*((cnst22-cnst21)/1000000)" ; move carrier from C' to Ca

;----- calc power levels for Spin-Lock using SL power defined by cnst10 (1H) and cnst11 (15N)
"p10=500*500/(cnst10+0.01)" ;calculate pulse length for 1H CWx during 15N zeta-purge and relax
"plw10=p1*p1*plw1/(p10*p10)";calculate spin-lock power level for 1H CWx during 15N zeta-purge and relax
"d22=(p10*2/PI)-(p1*4/PI)" ; 1st delay to align 1H along X before CWx
"d29=p1*2/PI-2u" ; 2nd delay to align 1H along X before CWx
"p11=500*500/(cnst11+0.01)" ;calculate spin-lock pulse length for 15N relax
"plw11=p7*p7*plw7/(p11*p11)"
"p34=d24"
"spw34=plw11";calculate spin-lock power level for 15N relax
"d7=p7+2u"

;------ watergate + detection
"p5=1500u*600/bf1" ;shaped pulse on water (1.5m@14.2T)
"spw5=p1*p1*plw1/(p5*p5*integfac5*integfac5)"
"p31=250u" ; 1 kHz dec. on 15N
"plw31=p7*p7*plw7/(p31*p31)" ; calculate 15N decoupling power level for 1kHz dec (p31=250u)


1 ze
1m
2 2m do:f3
1m do:f2
1u fq=0:f3
d11
3 3m
4 2m do:f3
1m do:f2
10u pl1:f1
10u pl7:f3

;--------- d1 recovery delay---------
```

```
d1
1m UNBLKGRAD
(p7 ph0):f3
p22:gp1
d16 pl0:f1

;------ start selective/non-selective 1H excitation
1u fq=cnst40(bf ppm):f1
1u pl1:f1

#ifdef Hsele
1u pl7:f3
(p7 ph3):f3
1u pl31:f3
1u cw:f3
(p61:sp61 ph0):f1
1u do:f3
1u pl7:f3
(p7 ph1):f3
1u pl1:f1
(p1 ph2):f1
p22:gp11
d16
(p1 ph0):f1
#else /*Hsele*/
1u fq=0:f1
(p5:sp5 ph2):f1
1u pl1:f1
1u fq=cnst40(bf ppm):f1
(p1 ph0):f1
#endif /*Hsele*/

;--- start HaHa or SFPT 1H to 15N
#ifdef SFPT1
(p15:sp17 ph7):f1
(p15:sp18 ph8):f3
```

```
#else /*SFPT1*/
(center (p18:sp19 ph7):f1 (p18:sp20 ph8):f3)
#endif /*SFPT1*/
1u pl1:f1
1u pl7:f3

;--- purge HzNz
(p7 ph1):f3
1u
(p1 ph2):f1
p22:gp2
d16 pl1:f1

;---- align 1H on x before CWx, start purge element
  (p1 ph11):f1
  d22
  (p1 ph0):f1
  (p1*2 ph11):f1
  d29
  1u pl10:f1
  1u cw:f1
#ifdef Nsele
(p63:sp63 ph13):f3
#else
 (p7 ph13):f3
#endif /*Nsele**/
DELTA3
  1u pl7:f3
  (p7 ph2):f3
  1u do:f1
  1u pl1:f1
  d29
  (p1*2 ph12):f1
  (p1 ph0):f1
  d22
  (p1 ph12):f1
```

```
p22:gp3
d16
1u pl1:f1
1u pl4:f2
1u pl7:f3

;---- align 1H on x before CWx, end purge element, start relax
  (p1 ph11):f1
  d22
  (p1 ph0):f1
  (p1*2 ph11):f1
  d29
  1u pl10:f1
  1u cw:f1
  d23
if "cnst0==0" goto 110 ;skip relax block during testing

;--- align 15N and relax
if "l5%l6==0" goto 100

;---- start 1st Tcomp
if "vc==cnst18"
{
  2u
}
else
{
"l11=cnst18-vc"
  1u fq=0:f3
  1u offTemp:f3
  901 (p34:sp34 ph0):f3
  lo to 901 times l11
}

;---- end 1st Tcomp., start align 15N with SLx using hard pulse with matched tilt-angle using va3
1u fq=0:f3
```

```
          1u pl7:f3
  1u va3:f3
  if "offRes>0"
  {
(p7 ph16):f3
          }
  else
  {
(p7 ph15):f3
          }
  1u offRes:f3

;--- end align 15N with SLx, actual relaxation element
902 (p34:sp34 ph0):f3
    (p34:sp34 ph0):f3
  lo to 902 times vc

;--- end of actual relaxation element, start align 15N with Z using hard pulse with matched tilt-angle using va3
          1u fq=0:f3
  1u pl7:f3
  1u va3:f3
  if "offRes>0"
  {
(p7 ph15):f3
          }
 else
 {
(p7 ph16):f3
          }

;---- end align 15N with Z, start 2nd Tcomp
if "vc==cnst18"
{
  2u
}
else
```

```
{
"l11=cnst18-vc"
  1u offTemp:f3
  903 (p34:sp34 ph0):f3
  lo to 903 times l11
  1u fq=0:f3
}
goto 110

;---- end vc!=0, start vc=0
100 1u fq=0:f3
1u offTemp:f3
"l12=cnst18"
d7
905 (p34:sp34 ph0):f3
    (p34:sp34 ph0):f3
  lo to 905 times l12
d7

;--- end vc=0, end CWx align Hz, tau_eq
110 1u pl7:f3
1u fq=0:f3
111 1u
  d23
  1u do:f1
  1u pl1:f1
  d29
  (p1*2 ph12):f1
  (p1 ph0):f1
  d22
  (p1 ph12):f1
p22:gp4
d16

;--- back-transfer SE+PT or pure PT
1u fq=cnst40(bf ppm):f1
```

```
(p7 ph1):f3
#ifdef SFPT2
(p15:sp18 ph6):f3
(p15:sp17 ph5):f1
#else /*SFPT2*/
(center (p18:sp19 ph5):f1 (p18:sp20 ph6):f3)
#endif /*SFPT2*/
1u pl1:f1
1u pl7:f3
1u fq=0:f1
p25:gp5
d17 pl0:f1
(p5:sp5 ph0):f1
1u pl1:f1
(p1*2 ph2):f1
1u pl0:f1
(p5:sp5 ph0):f1
p25:gp5
"d18=d17-2u"
d18

;---- start acqu
999 1u pl31:f3
 1u BLKGRAD
go=2 ph31 cpds3:f3
500u do:f3
500u do:f2
d11 wr #0 if #0 zd
 1m offRes.inc
 1m va3.inc
if "l5%l6==0" goto 888
 lo to 3 times l7
888 1m iu5  ;; controls skipping of offset incrementation for reference plane (l5=0 if vc=0)
 250u offRes.res
 250u va3.res
 500u vc.inc
```

```
 lo to 4 times l6
 500u ru5
 500u vc.res
1m do:f3
exit

ph0=0
ph1=1
ph2=2
ph3=3

ph7=1 3
ph8=2 2 0 0
ph13=0 0 0 0 2 2 2 2
ph6=0 0 0 0 0 0 0 0 2 2 2 2 2 2 2 2
ph5=0 0 0 0 0 0 0 0 0 0 0 0 0 0 0 0
    2 2 2 2 2 2 2 2 2 2 2 2 2 2 2 2

ph11=1 1 3 3
ph12=3 3 1 1

ph15=3
ph16=1

ph31=0 2 2 0 2 0 0 2 2 0 0 2 0 2 2 0
     2 0 0 2 0 2 2 0 0 2 2 0 2 0 0 2

;-------------NOTES----------------------
;o1p=4.7 ppm
;o2p=176 ppm (CO)
;o3p=set to resonance of interest, ppm
;NS=32*n

; pulses and power levels
;pl0: 120 dB
;p1: 90 deg hard 1H pulse @pl1
```

;pl1: 1H 90 deg
;p3: 90 deg hard 13C pulse @pl2
;pl2: 13C 90 deg
;p4: 90 deg selective on 13Ca @pl4
;pl4: 13Ca selective 90 deg
;p7: 90 deg hard 15N pulse @pl7
;pl7: 15N 90 deg

;spnam4 Squa100.1000
;spoal4 0.5

;p5: water flip-back pulse duration
;spnam5 Sinc1.1000
;spoal5 0.5

;p6: rSFPT spin-lock pulse length corresponding to power level cnst6 (us)
;p8: HaHa spin-lock pulse length corresponding to power level cnst8 (us)

;p15: duration of SFPT transfer spin-lock
;p18: duration of HaHa transfer spin-lock

;p61: 90 deg 1H selective pulse (1-20ms) 1H CSSF
;p63: 90 deg 15N selective pulse (10-200ms) 15N CSSF

;p10: calculate pulse length for 1H CWx during 15N CSSF
;pl10: calculate spin-lock power level for 1H CWx during 15N CSSF
;p11: calculate pulse length for 15N Spin-Lock during relax
;pl11: calculate 15N spin-lock power level during relax

;sp17: rSFPT 1H spin-lock power level (W)
;spnam17: Squa100.1000
;sp18: rSFPT 15N spin-lock power level (W)
;spnam18: Squa100.1000
;sp19: HaHa 1H spin-lock power level (W)
;spnam19: Squa100.1000
;sp20: HaHa 15N spin-lock power level (W)

;spnam20: Squa100.1000
;sp61: selective Hz->Hxy for initial excitation
;spnam61 Sinc1.1000
;sp63: selective Nz->Nxy during zeta element
;spnam63 GOLEM.1000
;sp34: 15N spin-lock pulse power level
;spnam34: Squa100.1000

;spoal16 0.5
;spoal17 0.5
;spoal18 0.5
;spoal19 0.5
;spoal20 0.5
;spoal34 0.5
;spoal61 1
;spoal63 1

;CPDPRG3: garp4 (aq 15N decoupling)
;pcpd3: 15N decoupling (250u @pl31)
;pl31: 15N decoupling power

;--- cnsts
;cnst0: is !=0 for acqu, is 0 without relax block for testing
;cnst3: 15N offset (Hz) of unwanted peak to be removed during zeta delay
;cnst6: for calculation of power level for rSFPT transfer (45 Hz)
;cnst8: for calculation of power level for HaHa transfer (45 Hz)
;cnst9: for calculation of delay for HaHa transfer (90 Hz)
;cnst10: 1H SL power during 15N CSSF or relax (Hz)
;cnst11: 15N SL during relax (Hz)
;cnst18: maximum number of loops in vclist
;cnst40: 1H offset of selected resonance (ppm)

;d23: equilibration period ~1/k_ex (5 ms for k_ex = 200/s)
;d24: relaxation delay *2*vc
;d22: 1st delay to align CWx with spin-lock
;d29: 2nd delay to align CWx align with spin-lock

;loops and lists
;l6: number of delays in vclist
;l7: number of offsets for off-resonance fq1list (offRes:f3)
;valist: variable amplitude (va3:f3) to align 15N mag. before and after spin-lock with matched tilt angle to offset in fq1list (offRes:f3)

; gradients
;p22: purge Nz (1m)
;p25: watergate (500u)

;for z-only gradients
;gpz1: -66%
;gpz2: 45%
;gpz3: -73%
;gpz4: 57%
;gpz5: 83%
;gpz11: 51%

6.2.4. SNIPER_N15Rex

```
;SNIPER_N15Rex
/****************************************************************
SNIPER ^{15}N Rex zz-exchange on per resonances basis using Hartmann-Hahn 15N-edited 1H (1D) spectra
with optional rSFPT for selective polarization in 'out' transfer (flag SFPT1)
with optional 15N (flag Nsele) and 1H (flag Hsele) selective excitation during spin-locked 1H and 15N mag.
with pulse-delay 1H alignment with CWx during 15N selective excitation and relaxation elements
with watergate before readout
tau_eq is performed during CWx before and after relax
written by WA 2024/12/31
****************************************************************/
; zgoptns: SFPT1, Nsele, Hsele

#include <Avance.incl>
#include <Grad.incl>
#include <Delay.incl>

define list<loopcounter> zzc    = <$VCLIST>  ;loopcounter list for relax delays


"d11=30m"
"d16=200u" ; long homospoil rec.
"d17=100u" ; short homospoil rec.
"d18=d17-2u"


;--- calc HaHa transfer and delay for zeta-purge
"cnst9=90" ; J_NH for HH
"DELTA=1s/(cnst9)" ; 1/J_NH for HH
"DELTA1=1s/(4*94)" ; 0.25/J_NH for RRI


#ifdef SFPT1
"p15=DELTA/sqrt(2)" ; pulse duration for SFPT
```

```
"p6=500*500/(cnst6+0.01)" ; calculate SFPT spin-lock pulse length (us)
"spw17=p1*p1*plw1/(p6*p6*integfac17*integfac17)" ; calculate 1H SFPT spin-lock power level (W)
"spw18=p7*p7*plw7/(p6*p6*integfac18*integfac18)" ; calculate 15N SFPT spin-lock power level (W)
#endif

"p18=DELTA"  ; pulse duration for HaHa
"p8=500*500/(cnst8+0.01)" ; calculate HaHa spin-lock pulse length (us)
"spw19=p1*p1*plw1/(p8*p8*integfac19*integfac19)" ; calculate 1H HaHa spin-lock power level (W)
"spw20=p7*p7*plw7/(p8*p8*integfac20*integfac20)" ; calculate 15N HaHa spin-lock power level (W)

;-- cleaning element and selective pulse calibration
"DELTA3=1/(cnst3*4+0.0001)" ;zeta delay to remove unwanted peak cnst3 (delta, Hz) away from the resonance
#ifdef Nsele
"spw63=p7*p7*plw7/(p63*p63*integfac63*integfac63)"
"spoal63=1"
#endif

#ifdef Hsele
"spw61=p1*p1*plw1/(p61*p61*integfac61*integfac61)"
"spoal61=1"
#endif

;--- calc 13C pulses
"p4=23.7u*600/bf1"
"plw4=p3*p3*plw2/(p4*p4)"
"spw4=plw4"
"cnst22=56" ; offset Ca
"cnst21=176" ; offset C'
"spoffs4=bf2*((cnst22-cnst21)/1000000)" ; move carrier from C' to Ca

;----- calc power levels for Spin-Lock using SL power defined by cnst10 (1H)
"p10=500*500/(cnst10+0.01)" ;calculate pulse length for 1H CWx during 15N zeta-purge and relax
"plw10=p1*p1*plw1/(p10*p10)";calculate spin-lock power level for 1H CWx during 15N zeta-purge and relax
"d22=(p10*2/PI)-(p1*4/PI)" ; 1st delay to align 1H along X before CWx
"d29=p1*2/PI-2u" ; 2nd delay to align 1H along X before CWx
```

```
;----- ZZ relaxation element delays
"p13=1600u*600/bf1"
"spw13=4*p1*p1*plw1/(p13*p13*integfac13*integfac13)"
"spoffs13=(2340*bf1/600)/1000000"
"d24=20m"
"d25=0.5*(d24-p13)"
"p19=p1-0.3u"

;------ watergate + detection
"p5=1500u*600/bf1" ;shaped pulse on water (1.5m@14.2T)
"spw5=p1*p1*plw1/(p5*p5*integfac5*integfac5)"
"p31=250u" ; 1 kHz dec. on 15N
"plw31=p7*p7*plw7/(p31*p31)" ; calculate 15N decoupling power level for 1kHz dec (p31=250u)


1 ze
1m
2 2m do:f3
1m do:f2
1u fq=0:f3
d11
3 3m
4 2m do:f3
1m do:f2
10u pl1:f1
10u pl7:f3

;--------- d1 recovery delay---------
d1
1m UNBLKGRAD
(p7 ph0):f3
p22:gp1
d16 pl0:f1

;------ start selective/non-selective 1H excitation
1u fq=cnst40(bf ppm):f1
```

```
1u pl1:f1

#ifdef Hsele
1u pl7:f3
(p7 ph3):f3
1u pl31:f3
1u cw:f3
(p61:sp61 ph0):f1
1u do:f3
1u pl7:f3
(p7 ph1):f3
1u pl1:f1
(p1 ph2):f1
p22:gp11
d16
(p1 ph0):f1
#else /*Hsele*/
1u fq=0:f1
(p5:sp5 ph2):f1
1u pl1:f1
1u fq=cnst40(bf ppm):f1
(p1 ph0):f1
#endif /*Hsele*/

;--- start HaHa or SFPT 1H to 15N
#ifdef SFPT1
(p15:sp17 ph7):f1
(p15:sp18 ph8):f3
#else /*SFPT1*/
(center (p18:sp19 ph7):f1 (p18:sp20 ph8):f3)
#endif /*SFPT1*/
1u pl1:f1
1u pl7:f3

;--- purge HzNz
(p7 ph1):f3
```

```
1u
(p1 ph2):f1
p22:gp2
d16 pl1:f1

;---- align 1H on x before CWx, start purge element
  (p1 ph11):f1
  d22
  (p1 ph0):f1
  (p1*2 ph11):f1
  d29
  1u pl10:f1
  1u cw:f1
#ifdef Nsele
(p63:sp63 ph13):f3
#else
 (p7 ph13):f3
#endif /*Nsele**/
DELTA3
  1u pl7:f3
  (p7 ph2):f3
  1u do:f1
  1u pl1:f1
  d29
  (p1*2 ph12):f1
  (p1 ph0):f1
  d22
  (p1 ph12):f1
p22:gp3
d16
1u pl1:f1
1u pl4:f2
1u pl7:f3

;---- ZZ-exchange
1u fq=0:f1
```

```
if "zzc==0" goto 82

81 d25 pl4:f2
   (center (p13:sp13 ph2):f1 (p4*2 ph0 p4*2:sp4 ph0):f2)
   d25 pl4:f2
 lo to 81 times zzc

82 1u
p22:gp4
d16
1u pl1:f1
1u pl7:f3

;-------- back INEPT with watergate or gradient-aided sensitivity-enhanced schme (flag 'SE') ---------------------------
1u fq=cnst40(bf ppm):f1
 (p7 ph0):f3
 "DELTA2=DELTA1-200u"
 DELTA2 gron0
 200u groff
 (center(p1*2 ph0):f1 (p7*2 ph18):f3)
 DELTA2 gron0
 200u groff
 (p7 ph1):f3
 1u fq=0:f1
 (p5:sp5 ph16):f1
 1u pl1:f1
 (p1 ph16):f1
;----- water-gate ----------------------------
 p25:gp5
 d17 pl0:f1
 "DELTA5=2.25m-p25-d17-p5-p7-1u"
 DELTA5
 (p5:sp5 ph0):f1
 1u pl1:f1
 (center (p1*2 ph2):f1 (p7*2 ph0):f3)
 1u pl0:f1
```

```
 (p5:sp5 ph0):f1
 DELTA5
 p25:gp5
 d18

;---- start acqu
999 1u pl31:f3
 1u BLKGRAD
go=2 ph31 cpds3:f3
500u do:f3
500u do:f2
d11 wr #0 if #0 zd
 500u zzc.inc
 lo to 3 times l6
 500u zzc.res
1m do:f3
exit

ph0=0
ph1=1
ph2=2
ph3=3

ph7=1 3
ph8=2 2 0 0
ph13=0 0 0 0 2 2 2 2
ph16=0 0 0 0 0 0 0 0
     2 2 2 2 2 2 2 2

ph18=0 2

ph11=1 1 3 3
ph12=3 3 1 1

ph31=0 2 2 0 2 0 0 2 2 0 0 2 0 2 2 0
```

```
;-------------NOTES----------------------
;o1p=4.7 ppm
;o2p=176 ppm (CO)
;o3p=set to resonance of interest, ppm
;NS=16*n

; pulses and power levels
;pl0: 120 dB
;p1: 90 deg hard 1H pulse @pl1
;pl1: 1H 90 deg
;p3: 90 deg hard 13C pulse @pl2
;pl2: 13C 90 deg
;p4: 90 deg selective on 13Ca @pl4
;pl4: 13Ca selective 90 deg
;p7: 90 deg hard 15N pulse @pl7
;pl7: 15N 90 deg

;p5: water flip-back pulse duration
;spnam5 Sinc1.1000
;spoal5 0.5

;p6: rSFPT spin-lock pulse corresponding to power level cnst6 (us)
;p8: HaHa spin-lock pulse corresponding to power level cnst8 (us)

;p15: duration of rSFPT transfer spin-lock
;p18: duration of HaHa transfer spin-lock

;p61: pi/2 1H selective pulse (1-200ms)
;p63: pi/2 15N selective pulse (1-200ms)

;spnam4 Squa100.1000
;spnam5 Sinc1.1000
;spnam13 Ibrup2.1000
;spnam17 Squa100.1000
;spnam18 Squa100.1000
```

;spnam19 Squa100.1000
;spnam20 Squa100.1000
;spnam61: Sinc1.1000
;spnam63: GOLEM.1000
;spoal4 0.5
;spoal5: 0.5
;spoal13: 0.5
;spoal17: 0.5
;spoal18: 0.5
;spoal19: 0.5
;spoal20: 0.5
;spoal61: 1
;spoal63: 1

;p15: duration of rSFPT transfer spin-lock
;p18: duration of HaHa transfer spin-lock
;p13: pulse length for 1HN inversion
;sp13: calculate power for 1HN inversion
;sp17: rSFPT 1H spin-lock power level (W)
;sp18: rSFPT 15N spin-lock power level (W)
;sp19: HaHa 1H spin-lock power level (W)
;sp20: HaHa 15N spin-lock power level (W)

;p10: calculate pulse length for CWx during zeta-purge
;pl10: calculate spin-lock power level for CWx during zeta-purge

;CPDPRG3: garp4 (aq 15N decoupling)
;pcpd3: 15N decoupling (250u @pl31)
;pl31: 15N decoupling power

;--- cnsts
;cnst3: 15N offset (Hz) of unwanted peak to be removed during zeta delay
;cnst6: for calculation of power level for SFPT transfer (45 Hz)
;cnst8: for calculation of power level for HaHa transfer (45 Hz)
;cnst9: for calculation of delay for HaHa transfer (90 Hz)
;cnst10: 1H SL power during CWx relax

;cnst40: 1H offset of selected resonance

;d24: relaxation delay *vc
;d25: half relaxation delay corrected for p13 duration
;d22: 1st delay to align 15N with spin-lock
;d29: 2nd delay to align 15N align with spin-lock

;loops and lists
;l6: number of delays in vclist

; gradients
;p22: purge Nz (1m)
;p25: watergate (500u)

;for z-only gradients
;gpz0: 0.3%
;gpz1: 66%
;gpz2: 87%
;gpz3: -73%
;gpz4: 57%
;gpz5: 83%
;gpz11: 51%
;gpz12: 0.37%
;gpz13: 0.23%

6.2.5. SNIPER_N15nOe

```
;SNIPER_N15nOe
/****************************************************************
SNIPER ^{15}N het-nOe on per resonances basis using Hartmann-Hahn 15N-edited 1H (1D) spectra
with optional rSFPT for selective polarization in 'back' (flag SFPT2) transfer
with optional 15N (flag Nsele) and 1H (flag Hsele) selective excitation during spin-locked 1H and 15N mag.
with pulse-delay 1H alignment with CWx during 15N selective excitation and relaxation elements
with watergate before readout
written by WA 2024/12/31
****************************************************************/
; zgoptns: SFPT2, Nsele, Hsele
#include <Avance.incl>
#include <Grad.incl>
#include <Delay.incl>

"d11=30m"
"d16=200u" ; long homospoil rec.
"d17=100u" ; short homospoil rec.

"p5=1500u*600/bf1" ;shaped pulse on water (1.5m@14.2T)
"spw5=p1*p1*plw1/(p5*p5*integfac5*integfac5)"
"d8=p1"
"l2=1"

;--- calc HaHa transfer and delay for zeta-purge
"cnst9=90" ; J_NH for HH
"DELTA=1s/(cnst9)" ; 1/J_NH for HH

"p18=DELTA"
"p8=500*500/(cnst8+0.01)"
"spw19=p1*p1*plw1/(p8*p8*integfac19*integfac19)" ; calculate 1H HH spin-lock power level (W)
"spw20=p7*p7*plw7/(p8*p8*integfac20*integfac20)" ; calculate 15N HH spin-lock power level (W)

#ifdef SFPT2
```

```
"p15=DELTA/sqrt(2)" ; pulse length for HH
"p6=500*500/(cnst6+0.01)" ; calculate HH spin-lock pulse length (us)
"spw17=p1*p1*plw1/(p6*p6*integfac17*integfac17)" ; calculate 1H HH spin-lock power level (W)
"spw18=p7*p7*plw7/(p6*p6*integfac18*integfac18)" ; calculate 15N HH spin-lock power level (W)
#endif

"DELTA3=1/(cnst3*4+0.0001)" ;zeta delay to remove unwanted peak cnst3 (delta, Hz) away from the resonance
;----- calc power levels for Spin-Lock using SL power defined by cnst10 (1H) and cnst11 (15N)
"p10=500*500/(cnst10+0.01)" ;calculate pulse length for 1H CWx during 15N relax
"plw10=p1*p1*plw1/(p10*p10)";calculate spin-lock power level for 1H CWx during 15N relax
"d22=(p10*2/PI)-(p1*4/PI)" ; 1st delay to align 1H along X before CWx
"d29=p1*2/PI-2u" ; 2nd delay to align 1H along X before CWx

#ifdef Nsele
"spw63=p7*p7*plw7/(p63*p63*integfac63*integfac63)"
"spoal63=1"
#endif

#ifdef Hsele
"spw61=p1*p1*plw1/(p61*p61*integfac61*integfac61)"
"spoal61=1"
#endif

"p31=250u"
"plw31=p7*p7*plw7/(p31*p31)"


1 ze
1m
2 3m do:f3
d11
3 3m
4 1m
5 1m do:f3
10u pl1:f1
10u pl4:f2
```

```
10u pl7:f3
if "cnst12==0" goto 12
(p7 ph0):f3 ; 90N pulse before d1
12 1u
d1
10u UNBLKGRAD
;--------------- 1H saturation period-----------------------
1u fq=cnst40(bf ppm):f1
if "l2 == 1" goto 9
8 11m
(p1*2 ph0):f1
11m
lo to 8 times l8
goto 10
9 11m
d8*2
11m
lo to 9 times l8
10 10u
;---- align 1H on x before CWx, start purge element
  (p1 ph11):f1
  d22
  (p1 ph0):f1
  (p1*2 ph11):f1
  d29
  1u pl10:f1
  1u cw:f1
#ifdef Nsele
(p63:sp63 ph13):f3
#else
 (p7 ph13):f3
#endif /*Nsele**/
DELTA3
  1u pl7:f3
  (p7 ph2):f3
  1u do:f1
```

```
  1u pl1:f1
  d29
  (p1*2 ph12):f1
  (p1 ph0):f1
  d22
  (p1 ph12):f1
p22:gp2
d16 pl1:f1
;--- back-transfer
1u fq=cnst40(bf ppm):f1
(p7 ph1):f3
#ifdef SFPT2
(p15:sp18 ph6):f3
(p15:sp17 ph5):f1
#else /*SFPT2*/
(center (p18:sp19 ph5):f1 (p18:sp20 ph6):f3)
#endif /*SFPT2*/
1u pl1:f1
1u pl7:f3
#ifdef Hsele
1u pl7:f3
(p7 ph3):f3
1u pl31:f3
1u cw:f3
1u pl1:f1
(p1 ph3):f1
(p61:sp61 ph1):f1
1u pl1:f1
1u do:f3
1u pl7:f3
(p7 ph1):f3
(p1 ph3):f1
p22:gp4
d16
(p1 ph1):f1
#endif /*Hsele*/
```

```
;---- end HH back-transfer, start watergate
1u fq=0:f1
p25:gp3
d17 pl0:f1
(p5:sp5 ph0):f1
1u pl1:f1
(p1*2 ph2):f1
1u pl0:f1
(p5:sp5 ph0):f1
p25:gp3
"d18=d17-2u"
d18
;---- start acqu
999 1u pl31:f3
 1u BLKGRAD
go=2 ph31 cpds3:f3
1m do:f3
d11 wr #0 if #0 zd
2m iu2
lo to 3 times 2
1m do:f3
exit

ph0=0
ph1=1
ph2=2
ph3=3

ph13=0 2
ph5=0 0 2 2
ph6=0 0 0 0 2 2 2 2

ph11=1 1 3 3
ph12=3 3 1 1
```

ph31=0 2 2 0 2 0 0 2

;-------------NOTES----------------------
;o1p=4.7 ppm
;o2p=176 ppm (CO)
;o3p=119 ppm
;NS=8*n
;in0=inf/2
;SW=1/(2*in0)

; 1H pulses
;p1: 90 deg hard 1H pulse @pl1
;pl1: 1H 90 deg
;pl0: 120 dB
;15N pulses
;p7 : 90 deg hard 15N pulse @pl7
;pl7 :15N 90 deg
;CPDPRG3: garp (aq 15N decoupling)
;pcpd3: 15N decoupling (200u @pl31)
;pl31: 15N decoupling power

;p5: shaped pulse on water during watergate (1.5 ms on 14.2T)
;p6: rSFPT spin-lock pulse corresponding to power level cnst6 (us) [ca. 50 Hz]
;p8: HaHa spin-lock pulse corresponding to power level cnst8 (us) [ca. 90 Hz]
;p15: duration of rSFPT transfer spin-lock
;p18: duration of HaHa transfer spin-lock
;sp17: rSFPT 1H spin-lock power level (W)
;sp18: rSFPT 15N spin-lock power level (W)
;sp19: HaHa 1H spin-lock power level (W)
;sp20: HaHa 15N spin-lock power level (W)

;p10: calculate pulse length for CWx during 15N relax
;pl10: calculate spin-lock power level for CWx during 15N relax

;spnam5 Sinc1.1000
;spnam17 Squa100.1000

;spnam18 Squa100.1000
;spnam19 Squa100.1000
;spnam20 Squa100.1000
;spnam61: Sinc1.1000
;spnam63: GOLEM.1000

;spoal5: 0.5
;spoal17: 0.5
;spoal18: 0.5
;spoal19: 0.5
;spoal20: 0.5
;spoal61: 1
;spoal63: 1

;--- cnsts
;cnst3: 15N offset (Hz) of unwanted peak to be removed during zeta delay
;cnst6: cnst for rSFPT spin-lock power level [ca. 50 Hz]
;cnst8: cnst for HaHa spin-lock power level [ca. 90 Hz]
;cnst9: J_NH for calculation of delay for PT transfer
;cnst10: 1H SL power during 15N relax
;cnst12: is 0 to skip 90deg on 15N before d1, is 1 for acqu
;cnst40: selected resonance 1H offset

; gradients
;p22: purge Nz 1000u
;p25: watergate (500u)

;for z-only gradients
;gpz2: 67%
;gpz3: 55%
;gpz4: -38%

7. References

[1] N. A. Farrow, O. W. Zhang, J. D. Forman-Kay, L. E. Kay, “A Heteronuclear Correlation Experiment for Simultaneous Determination of ^{15}N Longitudinal Decay and Chemical-Exchange Rates of Systems in Slow Equilibrium” *J. Biomol. NMR* **1994**, *4*, 727-734.

[2] M. Nishizawa, E. Walinda, D. Morimoto, K. Sugase, “Pinpoint analysis of a protein in slow exchange using F_1F_2-selective ZZ-exchange spectroscopy: assignment and kinetic analysis” *J. Biomol. NMR* **2020**, *74*, 205-211.

[3] M. Piotto, V. Saudek, V. Sklenar, “Gradient-Tailored Excitation for Single-Quantum NMR-Spectroscopy of Aqueous-Solutions” *J. Biomol. NMR* **1992**, *2*, 661-665.

[4] F. Delaglio, S. Grzesiek, G. W. Vuister, G. Zhu, J. Pfeifer, A. Bax, “NMRPipe - a Multidimensional Spectral Processing System Based on UNIX Pipes” *J. Biomol. NMR* **1995**, *6*, 277-293.

[5] M. W. Maciejewski, A. D. Schuyler, M. R. Gryk, I. I. Moraru, P. R. Romero, E. L. Ulrich, H. R. Eghbalnia, M. Livny, F. Delaglio, J. C. Hoch, “NMRbox: A Resource for Biomolecular NMR Computation” *Biophys. J.* **2017**, *112*, 1529-1534.

[6] W. Lee, M. Tonelli, J. L. Markley, “NMRFAM-SPARKY: enhanced software for biomolecular NMR spectroscopy” *Bioinformatics* **2015**, *31*, 1325-1327.

[7] J. J. Helmus, C. P. Jaroniec, “Nmrglue: an open source Python package for the analysis of multidimensional NMR data” *J. Biomol. NMR* **2013**, *55*, 355-367.

[8] M. Baias, P. E. S. Smith, K. N. Shen, L. A. Joachimiak, S. Zerko, W. Kozminski, J. Frydman, L. Frydman, “Structure and Dynamics of the Huntingtin Exon-1 N-Terminus: A Solution NMR Perspective” *J. Am. Chem. Soc.* **2017**, *139*, 1168-1176.

[9] G. R. Levy, K. N. Shen, Y. Gavrilov, P. E. S. Smith, Y. Levy, R. Chan, J. Frydman, L. Frydman, “Huntingtin's N-Terminus Rearrangements in the Presence of Membranes: A Joint Spectroscopic and Computational Perspective” *Acs Chemical Neuroscience* **2019**, *10*, 472-481.

[10] A. Urbanek, M. Popovic, A. Morato, A. Estana, C. A. Elena-Real, P. Mier, A. Fournet, F. Allemand, S. Delbecq, M. A. Andrade-Navarro, J. Cortes, N. Sibille, P. Bernado, “Flanking Regions Determine the Structure of the Poly-Glutamine in Huntingtin through Mechanisms Common among Glutamine-Rich Human Proteins” *Structure* **2020**, *28*, 733-746.

[11] P. Allard, M. Helgstrand, T. Härd, “The complete homogeneous master equation for a heteronuclear two-spin system in the basis of Cartesian product operators” *J. Magn. Reson.* **1998**, *134*, 7-16.

[12] M. S. Silver, R. I. Joseph, D. I. Hoult, “Highly Selective Pi/2 and Pi-Pulse Generation” *J. Magn. Reson.* **1984**, *59*, 347-351.

[13] E. Kupce, J. Boyd, I. D. Campbell, “Short Selective Pulses for Biochemical Applications” *J. Magn. Reson. Ser. B* **1995**, *106*, 300-303.

[14] H. Kessler, U. Anders, G. Gemmecker, S. Steuernagel, “Improvement of NMR Experiments by Employing Semiselective Half-Gaussian-Shaped Pulses” *J. Magn. Reson.* **1989**, *85*, 1-14.

[15] T. E. Skinner, T. O. Reiss, B. Luy, N. Khaneja, S. J. Glaser, “Application of optimal control theory to the design of broadband excitation pulses for high-resolution NMR” *J. Magn. Reson.* **2003**, *163*, 8-15.

[16] Y. X. Cui, Y. Z. Y. Jin, Y. Hou, X. X. Han, H. Y. Cao, L. E. Kay, T. R. Yuwen, “Optimization of TROSY- and anti-TROSY-based 15 N CPMG relaxation dispersion experiments through phase cycling” *J. Magn. Reson.* **2024**, *361*, 107629.

[17] D. M. Korzhnev, V. Y. Orekhov, L. E. Kay, “Off-resonance R1ρ NMR studies of exchange dynamics in proteins with low spin-lock fields: An application to a fyn SH3 domain” *J. Am. Chem. Soc.* **2005**, *127*, 713-721.

[18] P. Pelupessy, E. Chiarparin, “Hartmann-Hahn polarization transfer in liquids: An ideal tool for selective experiments” *Concept. Magn. Res.* **2000**, *12*, 103-124.

[19] P. Pelupessy, E. Chiarparin, G. Bodenhausen, “Excitation of selected proton signals in NMR of isotopically labeled macromolecules” *J. Magn. Reson.* **1999**, *138*, 178-181.

[20] E. R. R. Castellanos, D. P. Frueh, J. Wist, “Selective polarization transfer using a single rf field” *J. Chem. Phys.* **2008**, *129*, 014504.

[21] Y. Toyama, I. Shimada, “Frequency selective coherence transfer NMR spectroscopy to study the structural dynamics of high molecular weight proteins” *J. Magn. Reson.* **2019**, *304*, 62-77.

[22] D. M. Lesovoy, P. S. Georgoulia, T. Diercks, I. Matecko-Burmann, B. M. Burmann, E. V. Bocharov, W. Bermel, V. Y. Orekhov, “Unambiguous Tracking of Protein Phosphorylation by Fast High-Resolution FOSY NMR” *Angew. Chem. Int. Ed.* **2021**, *60*, 23540-23544.

[23] D. F. Hansen, L. E. Kay, “Improved magnetization alignment schemes for spin-lock relaxation experiments” *J. Biomol. NMR* **2007**, *37*, 245-255.

[24] T. R. Yuwen, J. P. Brady, L. E. Kay, “Probing Conformational Exchange in Weakly Interacting, Slowly Exchanging Protein Systems via Off-Resonance R1rho Experiments: Application to Studies of Protein Phase Separation” *J. Am. Chem. Soc.* **2018**, *140*, 2115-2126.

[25] E. Walinda, D. Morimoto, M. Shirakawa, K. Sugase, “F_1F_2-selective NMR spectroscopy” *J. Biomol. NMR* **2017**, *68*, 41-52.

[26] R. Freeman, “Selective Excitation in High-Resolution NMR” *Chem. Rev.* **1991**, *91*, 1397-1412.

[27] J. M. Nuzillard, R. Freeman, “Band-Selective Pulses Designed to Accommodate Relaxation” *J. Magn. Reson. Ser. A* **1994**, *107*, 113-118.

[28] N. A. Lakomek, J. F. Ying, A. Bax, “Measurement of ^{15}N relaxation rates in perdeuterated proteins by TROSY-based methods” *J. Biomol. NMR* **2012**, *53*, 209-221.

[29] J. A. Jones, P. Hodgkinson, A. L. Barker, P. J. Hore, “Optimal sampling strategies for the measurement of spin-spin relaxation times” *J. Magn. Reson. Ser. B* **1996**, *113*, 25-34.

[30] J. S. Alper, R. I. Gelb, “Standard Errors and Confidence-Intervals in Nonlinear-Regression - Comparison of Monte-Carlo and Parametric Statistics” *Journal of Physical Chemistry* **1990**, *94*, 4747-4751.
